# Oxazoline scaffold in synthesis of benzosiloxaboroles and related ring-expanded heterocycles: diverse reactivity, structural peculiarities and antimicrobial activity†

**DOI:** 10.1039/d2ra03910a

**Published:** 2022-08-16

**Authors:** Joanna Krajewska, Krzysztof Nowicki, Krzysztof Durka, Paulina H. Marek-Urban, Patrycja Wińska, Tomasz Stępniewski, Krzysztof Woźniak, Agnieszka E. Laudy, Sergiusz Luliński

**Affiliations:** Department of Pharmaceutical Microbiology, Medical University of Warsaw Banacha 1 b 02-097 Warsaw Poland alaudy@wp.pl; Warsaw University of Technology, Faculty of Chemistry Noakowskiego 3 00-664 Warsaw Poland sergiusz.lulinski@pw.edu.pl; GPCR Drug Discovery Lab, Research Programme on Biomedical Informatics (GRIB), Hospital del Mar Medical Research Institute (IMIM) – Department of Experimental and Health Sciences of Pompeu Fabra University (UPF) Carrer del Dr Aiguader, 88 08003 Barcelona Spain; University of Warsaw, Faculty of Chemistry Pasteura 1 02-093 Warsaw Poland

## Abstract

Two isomeric benzosiloxaborole derivatives 3a and 5a bearing fluorine and 4,4-dimethyl-2-oxazolin-2-yl substituents attached to the aromatic rings were obtained. Both compounds were prone to hydrolytic cleavage of the oxazoline ring after initial protonation or methylation of the nitrogen atom. The derivative 3c featuring *N*-methylammoniumalkyl ester functionality was successfully subjected to *N*-sulfonylation and *N*-acylation reactions to give respective derivatives which demonstrates its potential for modular synthesis of structurally extended benzosiloxaboroles. Compound 5c bearing *N*-ammoniumalkyl ester underwent conversion to a unique macrocyclic dimer due to siloxaborole ring opening. Furthermore, an unexpected 4-electron reduction of the oxazoline ring occurred during an attempted synthesis of 5a. The reaction gave rise to an unprecedented 7-membered heterocyclic system 4a comprising a relatively stable B–O–B–O–Si linkage and stabilized by an intramolecular N–B coordination. It could be cleaved to derivative 4c bearing BOH and SiMe_2_OH groups which acts as a pseudo-diol as demonstrated by formation of an adduct with Tavaborole. Apart from the multinuclear NMR spectroscopy characterization, crystal structures of the obtained products were determined in many cases by X-ray diffraction. Investigation of biological activity of the obtained compounds revealed that derivatives 3e and 3f with pendant *N*-methyl arylsulfonamide groups exhibit high activity against Gram-positive cocci such as methicillin-sensitive *Staphylococcus aureus* ATCC 6538P, methicillin-resistant *S. aureus* (MRSA) ATCC 43300 as well as the MRSA clinical strains, with MIC values in the range of 3.12–6.25 mg L^−1^. These two compounds also showed activity against *Enterococcus faecalis* ATCC 29212 and *Enterococcus faecium* ATCC 6057 (with MICs of 25–50 mg L^−1^). The results of the antimicrobial activity and cytotoxicity studies indicate that 3e and 3f can be considered as potential antibacterial agents, especially against *S. aureus* MRSA.

## Abstract 2

Transformations of oxazoline–benzosiloxaborole conjugates gave rise to novel boracyclic systems as well as functionalized derivatives featuring antibacterial activity.

## Graphical abstract

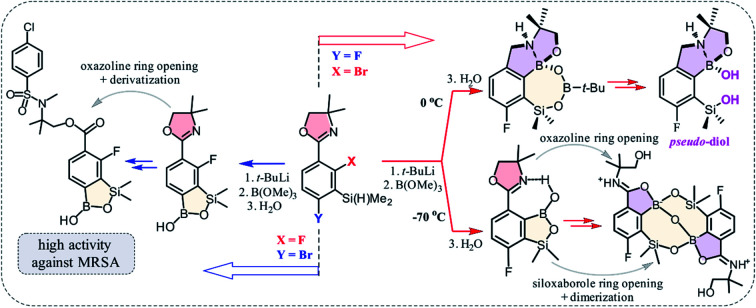

## Introduction

Benzoxaboroles constitute one of the most extensively studied groups of boron heterocycles which contain an endocyclic B–O linkage providing high thermodynamic stabilization.^1–3^ Recently, various heterocyclic organoboron compounds have attracted strong interest due to their promising properties associated with improved stability and Lewis acidity compared to analogous acyclic organoboranes.^4^ Thus, they are predestined for applications in medicine as they are hydrolytically stable and resistant to oxidation in air. Benzosiloxaboroles can be regarded as silicon bioisosteres of benzoxaboroles and show analogous physicochemical and biological properties. Indeed, available data indicate that selected benzosiloxaboroles are potent antimicrobial agents. For example, simple halogenated derivatives show strong antifungal activity whilst more extended systems, especially compounds with pendant arylsulfonate ArSO_3_ substituents were recognized as effective antibacterials, especially towards Gram-positive cocci such as *Staphylococcus aureus* (including methicillin-resistant *S. aureus* strains, MRSA), *Staphylococcus epidermidis*, and *Enterococcus faecalis*.^5^ In addition, a few benzosiloxaboroles were identified as effective inhibitors of KPC-2 β-lactamase responsible for resistance among clinical strains of Gram-negative rods *e.g. Klebsiella pneumoniae* towards β-lactam antibiotics, including carbapenems.^6^ Other applications of benzosiloxaboroles involved chemometric differential sensing of selected sugars.^7^ On the other hand, oxazolines and closely related oxazole heterocycles are useful synthons in medicinal chemistry.^8–12^ Examples of boronated aryl oxazolines are rare. The synthesis of *ortho*-boronated 2-phenyl-4,4-dimethyloxazoline was reported in 1986 but it was only reported as an intermediate prone to rapid hydrolysis resulting in a 2-carboxamidophenyl boronic acid derivative.^13^ However, three isomeric boronated 2-phenyl-4,4-dimethyloxazolines were successfully isolated in 2009 and used with varying success for Suzuki cross-coupling reactions.^14^ Since it is known that the biological properties of benzosiloxaboroles (and benzoxaboroles) are strongly tuned by pendant structurally diverse functional groups, we embarked on the preparation and characterization of compounds comprising 4,4-dimethyl-2-oxazoline rings attached to the benzosiloxaborole scaffold. It is worth noting here that the chemistry of arylboronic derivatives bearing nitrogen-based functionalities is diverse and offers many synthetic possibilities; various systems were also tested as potential antimicrobial agents.^15–18^ Since oxazolines have potential for subsequent transformations *via* ring cleavage, we also exploited this possibility in our work which gave rise to novel heterocyclic systems including derivative 4a featuring an unprecedented 7-membered ring comprising a BOBOSi linkage. The obtained new derivatives were comprehensively characterized including by single crystal X-ray diffraction analyses. In addition, comprehensive screening of antimicrobial activity was performed in order to complement the assessment of the structure–activity relationships of benzosiloxaboroles.

## Results and discussion

### Synthesis and structural characterization

**Scheme 1:**
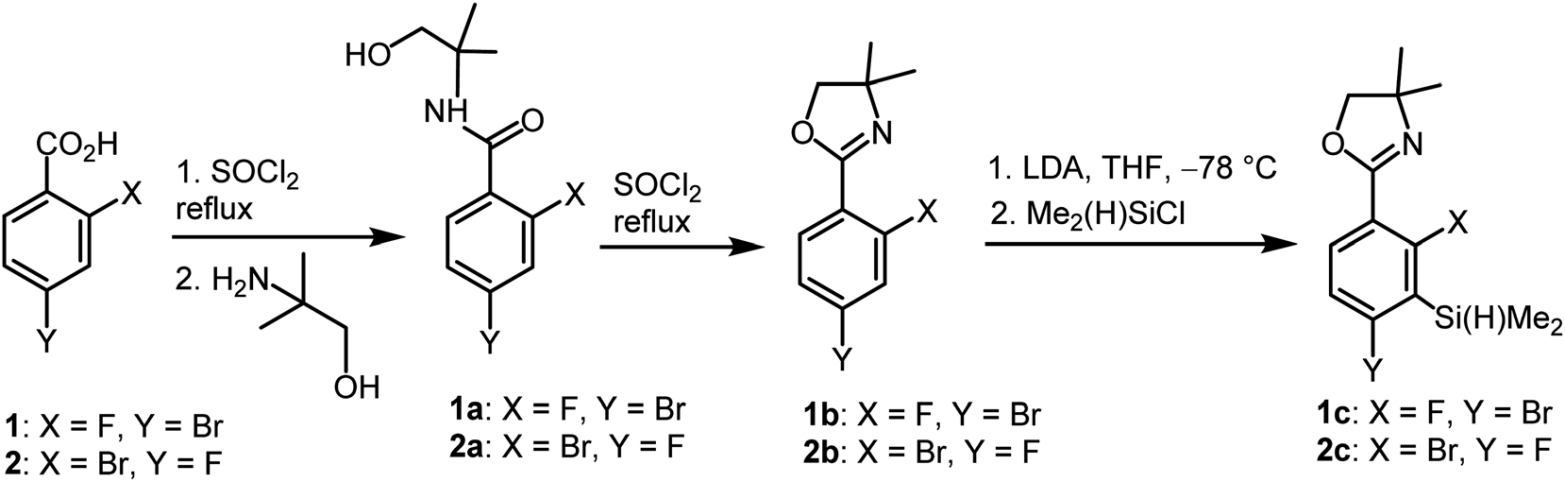
Synthesis of benzosiloxaborole precursors 1c and 2c.

The synthesis of oxazoline precursors 1b and 2b was performed using one of the general methods (Scheme 1).^19–21^ It involved conversion of 4-bromo-2-fluorobenzoic acids 1 and 2 to corresponding hydroxyamides 1a and 2a which involved treatment with SOCl_2_ to give benzoyl chlorides followed by addition of 2 equiv. of 2-amino-2-methylpropan-1-ol. The intermediate amides were subjected to dehydrative cyclization using SOCl_2_ in excess resulting in 2-aryl-4,4-dimethyl-2-oxazolines 1b and 2b in good yields (*ca.* 70%). In the next step, the lithiation of 1b and 2b occurred regioselectively at the position between fluorine and bromine using LDA/THF at −78 °C. It should be noted that the oxazoline substituent is generally recognized to act as strong *ortho*-directing group in aromatic lithiation which also provides significant thermodynamic stabilization for resulting aryllithiums.^22^ However, we were pleased to find that in both studied cases the cumulated *ortho*-acidifying effect of halogens seems to prevail strongly as subsequent trapping with Me_2_Si(H)Cl afforded respective products 1c and 2c. However, it is important to control the stoichiometry as in the case of 2b where the formation of a disilylated byproduct was observed.

**Scheme 2:**
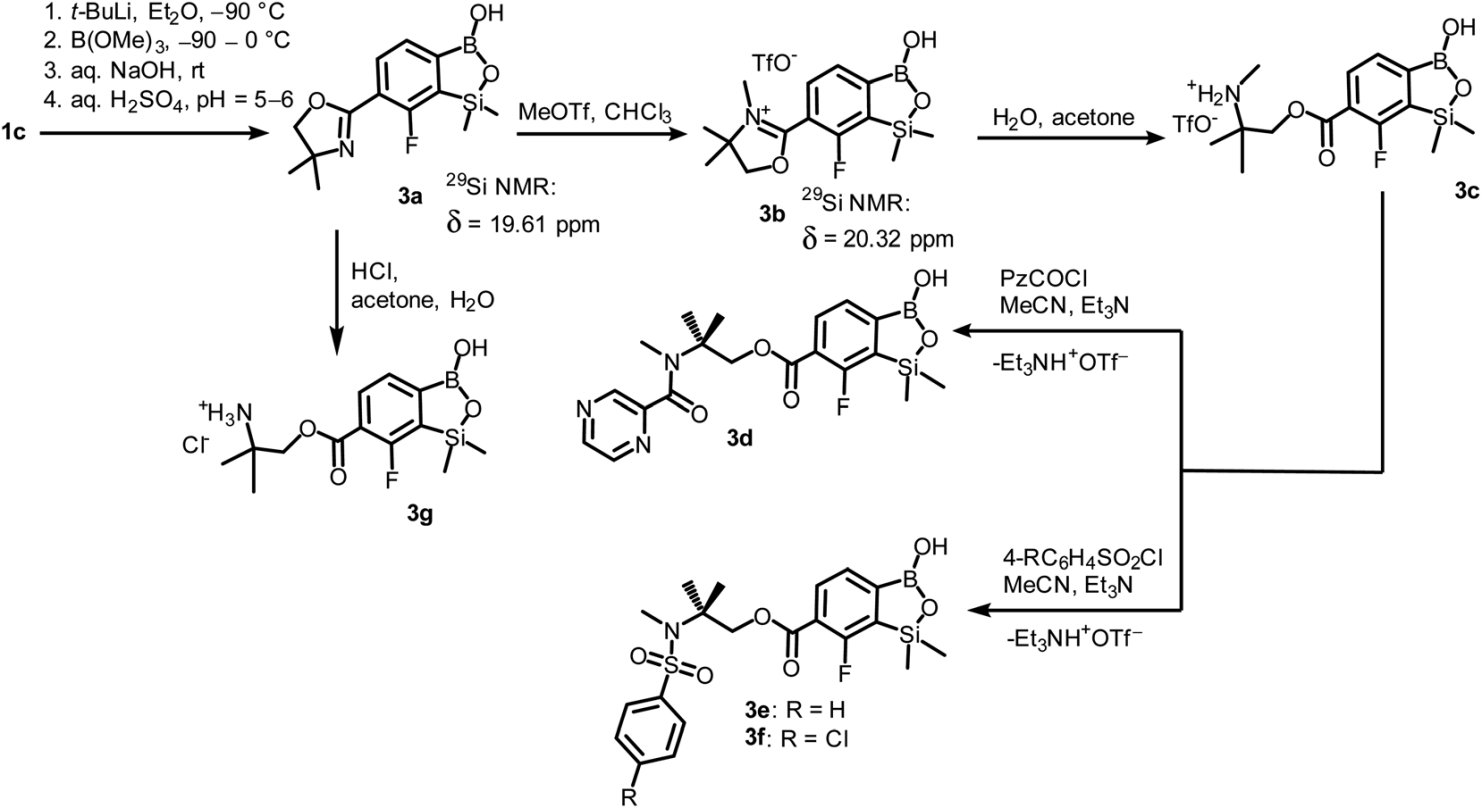
Synthesis of benzosiloxaborole 3a and its transformation to derivatives 3b–3g.

**Fig. 1:**
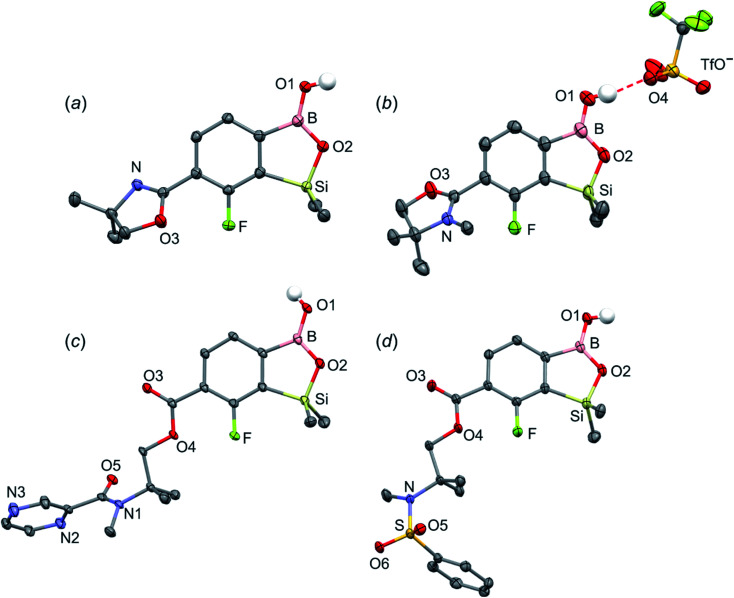
The molecular structures of (a) 3a, (b) 3b, (c) 3d, (d) 3e. Thermal motions given as ADPs at the 50% probability level. C–H hydrogen atoms were omitted for clarity. HB interaction between 3b^+^ and TfO^−^ is marked as red dashed line.

The conversion of 1c to oxazoline-substituted benzosiloxaborole 3a was performed using a general protocol described by us previously^5^ which involves the use of *t*-BuLi/Et_2_O at low temperature (<−90 °C) followed by trapping of aryllithium intermediates with B(OMe)_3_ (Scheme 2). After warming the mixture to *ca.* 0 °C, hydrolysis was initially carried out by addition of aq. NaOH which facilitates a cleavage of Si–H bonds. Subsequent neutralization with dilute aq. HCl resulted in precipitation of 3a which was isolated as a white solid; the process should be performed under precise pH control in order to avoid oxazoline protonation. The structure of compound 3a was confirmed by multinuclear (^1^H, ^13^C, ^11^B, ^19^F and ^29^Si) NMR spectroscopy. ^11^B NMR spectrum of 3a shows a resonance at 30.0 ppm typical of boronic acid derivatives with trigonal planar boron atom which means that there is no tendency to self-aggregation resulting from plausible formation of N–B dative bonds.^23^^29^Si NMR chemical shift of 19.61 ppm is in agreement with the values reported previously for other benzosiloxaboroles.^24,25^ In addition, single crystal X-ray diffraction revealed that geometric parameters of benzosiloxaborole core in 3a are very similar to those found previously in other derivatives whilst the oxazoline ring is twisted with respect to aromatic ring by 47.3(2)° (Fig. 1). It is worth noting that discrete molecules do not produce centrosymmetric dimeric motifs due to H-bonding interactions of boronic groups which are characteristic for most boronic acids^26^ and their cyclic analogues including benzoxa- and benzosiloxaboroles.^27^ In contrast, the molecules are assembled by means of OH⋯N hydrogen bonds (*d*_O⋯N_ = 2.752(2) Å, *d*_H⋯N_ = 1.89(2) Å, *α*_O–H⋯N_ = 169(2)°) to form infinite chains as a primary supramolecular motif (Fig. S1, ESI†).

Compound 3a was subjected to a subsequent functionalization involving hydrolytic opening of the oxazoline ring. The process is typically initiated by alkylation or protonation of the nitrogen atom which facilitates the nucleophilic attack owing to generation of positive charge on the oxazoline ring. Thus, we treated 3a with excess of MeOTf in chloroform which resulted in rapid precipitation of the ionic species 3b in the form of white relatively non-hygroscopic solid which can be handled under ambient conditions. This enabled full characterization by multinuclear NMR spectroscopy and X-ray diffraction (molecular structure is shown in Fig. 1b). The comparison of ^11^B and ^29^Si NMR data of 3a*vs.*3b shows that they are essentially unaffected by introduction of charge to the pendant oxazoline framework. The geometric parameters of the benzosiloxaborole cation of 3b and its precursor 3a are also very similar, although the oxazoline ring in crystal structure 3b is inversely twisted around C–C bond with respect to 3a molecule. The formation of hydrogen-bonded chain motif is hampered as B–OH group is arranged in HB interaction with TfO^−^ anion (*d*_O⋯N_ = 2.827(3) Å, *d*_H⋯N_ = 2.03(2) Å, *α*_O–H⋯N_ = 160(4)°). Upon addition of water to the acetone solution of 3b, the cleavage of the imine bond in oxazoline ring occurs readily at room temperature affording cleanly derivative 3c which comprises ester functionality decorated with *N*-methylammonium end group. It should be noted that oxazoline ring opening occurs frequently with the formation of a respective amide functionality.^28,29^ However, seminal mechanistic studies by Deslongchamps *et al.*^30,31^ confirmed by further examples^32,33^ showed that hydrolysis of oxazoline ring results in a respective aminoester. However, it is often prone to rapid intramolecular *N*-acylation yielding a final hydroxyamide product.^34,35^ The latter reaction can be suppressed by the protonation of the nitrogen atom in the aminoester intermediate. Compound 3c was subjected to subsequent derivatizations. Inspired by the potential of pyrazineamide motif in medicinal chemistry,^36–40^ we have obtained compound 3d by *N*-acylation of 3c with pyrazinoyl chloride. Furthermore we have also considered the importance of sulfonamides and converted 3c to benzosiloxaboroles 3e and 3f. The molecular structures of 3d and 3e were additionally confirmed by single crystal X-ray diffraction (Fig. 1c and d). In addition, we performed direct hydrolysis of 3a in a mixture of acetone and 2 M aq. HCl. This resulted in the ionic product 3g which is a close analogue of 3c. However, subsequent acylation and sulfonylation of 3g gave unsatisfactory results due to formation of mixtures of products which may be ascribed to a higher reactivity of NH_2_*versus* NHMe group.

**Scheme 3:**
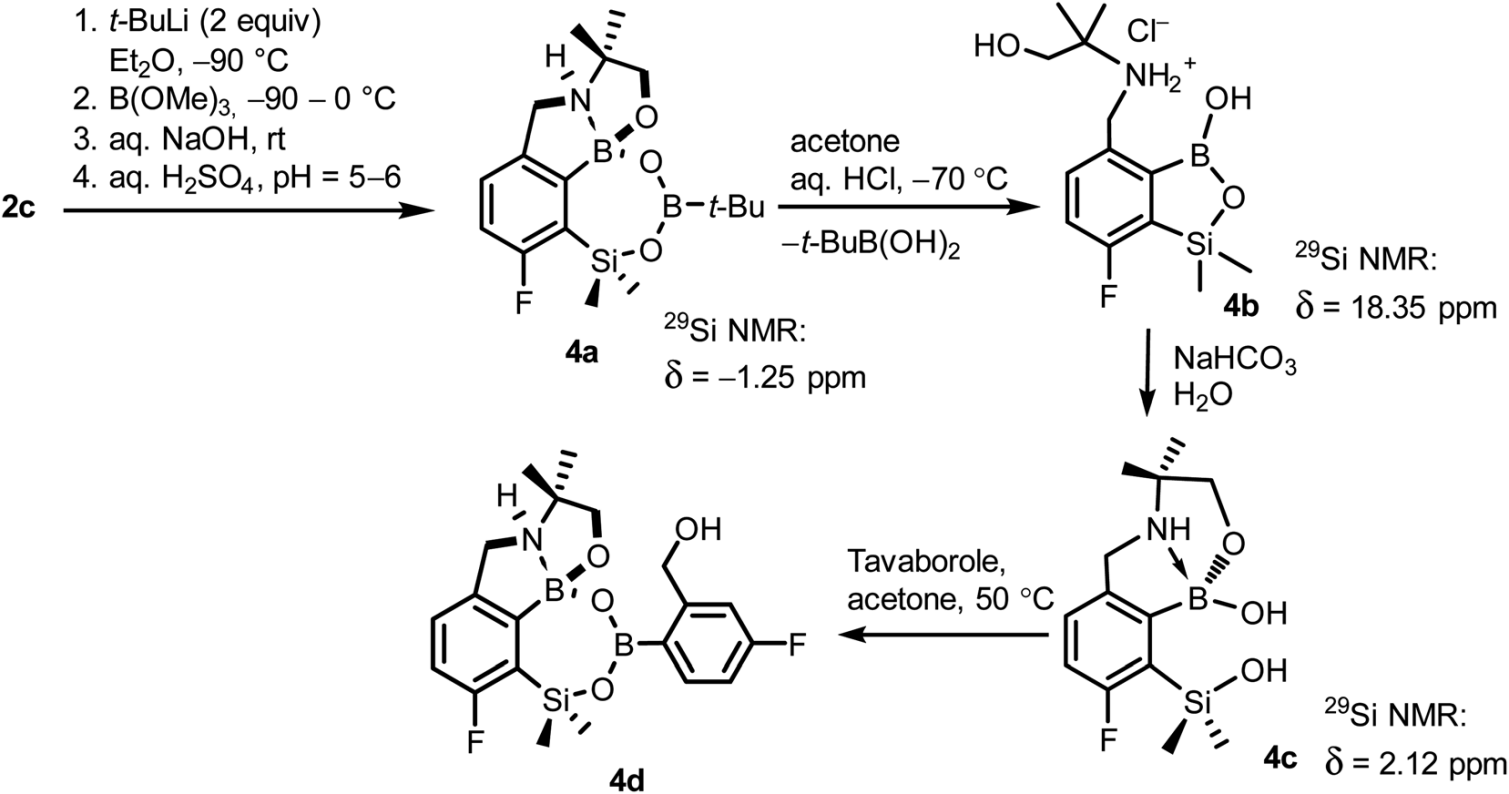
Synthesis of 4a–4d including general reaction conditions.

**Fig. 2:**
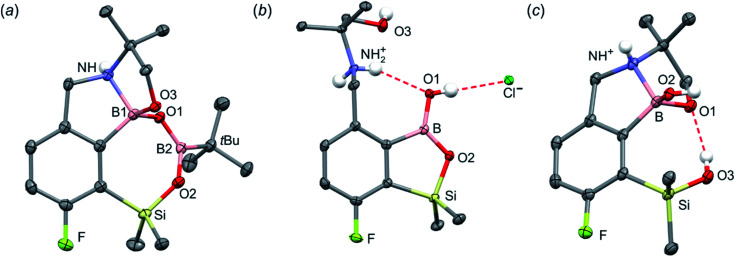
The molecular structures of (a) 4a, (b) 4b and (c) 4c. Thermal motions given as ADPs at the 50% probability level. C–H hydrogen atoms were omitted for clarity. HB in 4b (*d*_N⋯O1_ = 2.961(2) Å, *d*_H⋯O1_ = 2.26(2) Å, *α*_N–H⋯O1_ = 140(2)°; *d*_O1⋯Cl_ = 3.074(1) Å, *d*_H⋯Cl_ = 2.24(2) Å, *α*_O1–H⋯Cl_ = 172(2)°) and 4c (*d*_O3⋯O1_ = 2.826(1) Å, *d*_H⋯O1_ = 2.00(2) Å, *α*_O3–H⋯O1_ = 165(2)°) are marked as red dashed line.

**Fig. 3:**
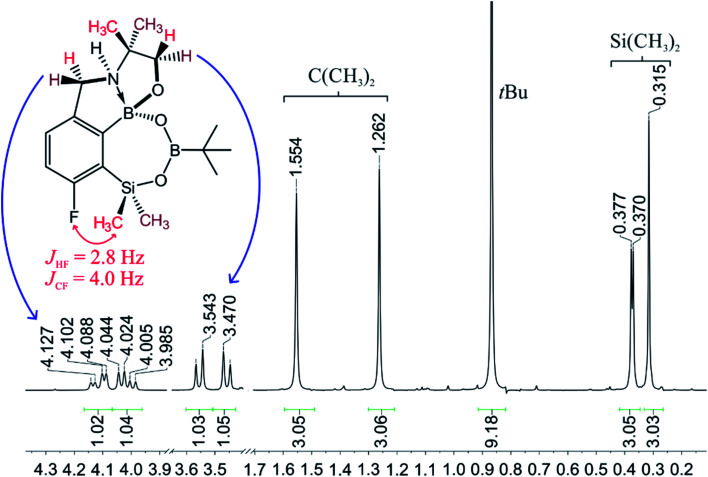
The fragment of ^1^H NMR spectrum of 4a showing the signals of diastereotopic protons.

When the protocol elaborated for synthesis 3a was used for preparation of its regioisomer 5a from 2c, a mixture of products was obtained. Subsequent workup resulted in the isolation of an unexpected product 4a (Scheme 3). The molecular structure of 4a was unambiguously determined by single crystal X-ray diffraction (Fig. 2a) and represents the first example of a seven-membered ring system comprising B–O–B–O–Si linkage. It crystallizes as a racemic mixture in centrosymmetric *P*2_1_/*c* space group. The geometric parameters of 4a including the B–O and Si–O bond distances are similar to those found in related systems such as boroxines and borosiloxane derivatives. The rather wide B–O–Si bond angle of 137.9(1)° is also characteristic for borosiloxane derivatives.^25^ The ring features a quasi-boat conformation which implies steric non-equivalence of Si-bound methyl groups manifested by the shortest H⋯F contact of 2.691(2) Å for one of them *vs.* 2.907(2) Å for the second one. One of boron atoms covalently bound to aromatic carbon and two oxygen atoms is four-coordinate due to existence of another dative bond with amine nitrogen which is relatively short (1.650(2) Å) considering a family of related arylboronic azaesters.^41^ The presence of B–N coordination bond is consistent with the presence of two boron-centered five-membered chelate rings. The ^1^H NMR spectrum of 4a is rather complicated which reflects the lack of symmetry owing to presence of the centre of chirality at the tetrahedral boron atom (Fig. 3a). As a result, multiplets of diastereotopic protons of both methylene groups as well as two separate singlets of methyl groups attached to the oxazoline ring are observed. Interestingly, one of Si-bound methyl group resonances is not a singlet but a doublet which can be explained by effective through-space coupling (*J*_HF_ = 2.8 Hz) with adjacent fluorine atom. A similar situation is observed in the ^13^C NMR spectrum which shows a doublet at 2.0 ppm (*J*_CF_ = 4.0 Hz) and a singlet at 1.8 ppm. This is in line with a closer contact between one of those methyl groups and F atom observed in the molecular structure determined by X-ray crystallography. ^11^B NMR spectrum of 4 shows two signals at 32.6 and 12.7 ppm (Fig. 3b); the latter value confirms the tetrahedral coordination of one of the boron atoms and is in the range characteristic for related arylboronic azaesters. ^29^Si NMR chemical shift of 4a is −1.2 ppm consistent with significantly higher shielding of silicon atom (by *ca.* 20 ppm) relative to benzosiloxaboroles. This can be explained by the release of strain at the silicon atom which is generated in five-membered heterocyclic ring of benzosiloxaboroles due to compression of Si–O–B bond angle by *ca.* 20° compared to the value observed in 4a. In addition, it is worth noting that addition of a drop of CF_3_SO_3_D/D_2_O to the solution of 4a in DMSO-*d*_6_ resulted in a strong simplification of the ^1^H NMR spectrum which can be generally explained by the collapse of chirality caused by protonation of NH group with concomitant formation of the trigonal planar boron centre. However, neutralization of the solution results in regeneration of 4a which suggests that the covalently bound scaffold of the molecule is relatively stable.

**Scheme 4:**
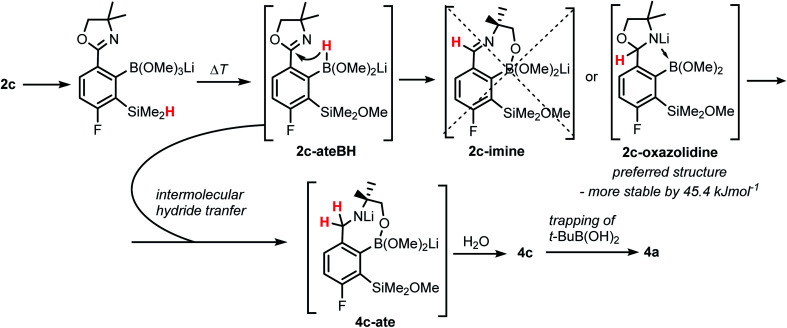
Proposed mechanism of the formation of 4a showing consecutive boron mediated hydride transfer steps from silicon to carbon atom.

The formation of 4a must involve 4-electron reduction of oxazoline ring. It is plausible that the process is preceded by hydride/methoxide interconversion between silicon and boron atoms in the intermediate ate complex which occurs during heating to ambient temperature. Such a process was observed previously and seems to be responsible for activation of Si–H bond in aryldimethylsilanes with anionic trialkoxyborate group at the *ortho* position.^42^ The resulting borohydride species 2c-ateBH undergoes an intramolecular hydride transfer to the oxazoline C2 atom. The process is somewhat related to the intramolecular hydrosilylation of cyano group to imine which was effected with Si–H bond activated by adjacent B(OMe)_3_^−^ group.^25^ We considered two structures of a plausible intermediate: the first one 2c-imine would result from expansion of the oxazoline ring initiated by the cleavage of the C–O bond. However, DFT calculations (with Me_2_O molecules added to fill the coordination sphere of lithium) indicate that the reduction of the C=N bond in 2c-ateBH leading to 2c-oxazolidine is strongly favourable (Scheme 4). However, the efficient formation of 4a implies that the subsequent intermolecular reduction of an intermediate (presumably 2c-oxazolidine) with the unreacted 2c-ateBH is strongly favourable. In other words, it seems that the initial two-electron reduction of oxazoline is the rate-limiting step which is followed by rapid conversion of an intermediate product to the benzylamine species 4c-ate. It should be stressed that the hydride transfer to oxazoline is effective owing to its intramolecular character as an analogous process was not observed during synthesis of 3a. The subsequent formation of 4a comprising unprecedented 7-membered ring with B–O–B–O–Si linkage presumably occurs through a trapping of an intermediate 4c possessing two hydroxyl groups attached to boron and silicon atoms. Such a diol-like species would combine with the molecule of *tert*-butylboronic acid which is generated from the reaction of excess of *t*-BuLi with B(OMe)_3_. Thus, the proposed explanation is based on the known tendency of boronic acids to form cyclic esters with various dihydroxyl compounds. In addition, the structure seems to be stabilized owing to the presence of dative N–B bond and also, a hydrophobic effect of bulky *tert*-butyl group. As a result, the reverse hydrolysis process seems to be disfavored. For some analogy, cyclic boronic esters with more lipophilic diols such as pinacol are much more stable towards hydrolysis than esters with ethylene glycol. However, heating of acidic solution of 4a in acetone/H_2_O resulted in degradation 7-membered heterocycle and formation of a benzosiloxaborole framework 4b with pendant *N*-(1-hydroxy-2-methylprop-2-yl)aminomethyl arm isolated as a hydrochloride salt. The structure of this product was confirmed by multinuclear NMR spectroscopy and single-crystal X-ray diffraction studies (Fig. 2b). Structural analysis shows that the combination of intermolecular hydrogen bond interactions involving BOH, NH_2_^+^ and Cl^−^ gives rise to infinite molecular chains (Fig. S5, ESI†) further assembled through CH_2_OH⋯Cl^−^ HB interactions. Subsequent neutralization of 4b with aq. NaHCO_3_ led to the cleavage of siloxaborole ring which clearly results from preferred formation of 4c featuring an 8-membered heterocyclic ring stabilized by an intramolecular N–B coordination (*d*_B–N_ = 1.667(2) Å), while Si–OH group acts as a donor of intramolecular hydrogen bond interaction (*d*_O3⋯O1_ = 2.826(1) Å, *d*_H⋯O1_ = 2.00(2) Å, *α*_O3–H⋯O1_ = 165(2)°, Fig. 2c). Overall, the process is analogous to chelation of boronic derivatives with various ethanolamine and diethanolamine derivatives which is well documented and used for protection of boron atom against nucleophilic attack.^43,44^ The DFT calculations confirm the higher stability of 4c over other hypothetical tautomeric structure comprising siloxaborole ring and pendant *N*-(1-hydroxy-2-methylprop-2-yl)aminomethyl arm (Δ*G*° = 34.2 kJ mol^−1^), 4b′, *i.e.*, the neutral form of 4b (Fig. S10, ESI†). In addition, the dehydration of the Si–OH and B–OH group is thermodynamically disfavored (Δ*G*° = 47.0 kJ mol^−1^) as it would generate considerable strain between both boracyclic rings. Notably, compound 4c does not undergo dehydrative condensation of BOH and SiMe_2_OH which would give rise to a dimeric species as observed for a related derivative.^25^ In contrast, it acts as a diol-like species and effectively traps Tavaborole to give a system 4d comprising again the 7-membered boracylic ring (Scheme 3). Theoretical calculations show that 4c is able to bind both aliphatic and aromatic boronic acids as well as catechol (see Scheme S1, ESI†).

**Scheme 5:**
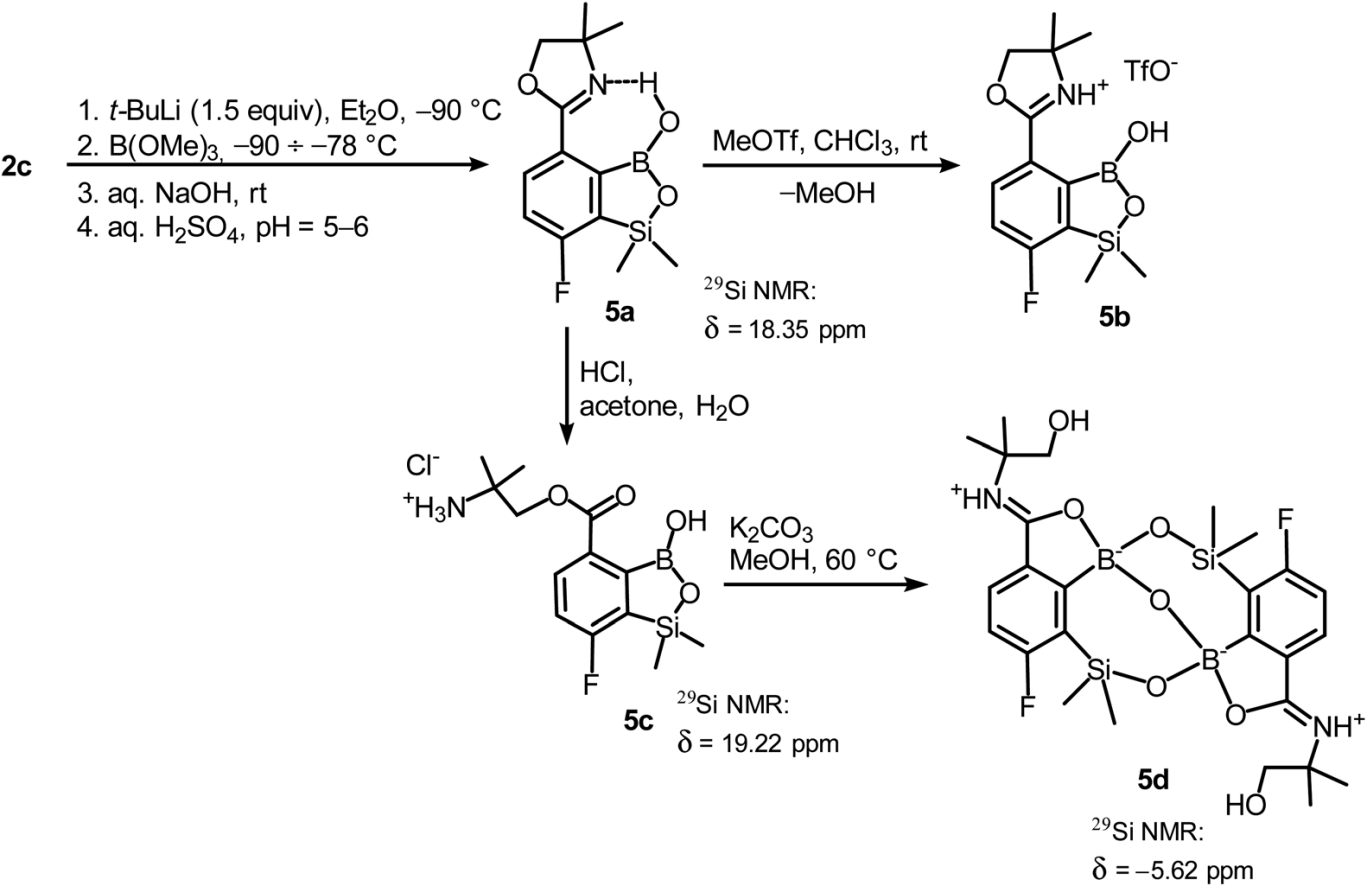
Synthesis of benzosiloxaborole 5a and its conversion to derivatives 5b–5d.

In order to obtain compound 5a, the synthetic protocol elaborated for 3a was modified. Most importantly, the hydrolysis of the intermediate ate complex was carried out at the low temperature (−70 °C) which prevented its transformations initiated by activation of the Si–H bond. Moreover, the amount of *t*-BuLi was reduced from 2 to 1.5 equiv. which was still sufficient to perform Br–Li exchange quantitatively. Overall, the introduced changes allowed to finally obtain 5a in satisfactory yield (*ca.* 60%) (Scheme 5). Notably, in the ^1^H NMR spectrum of 5a the broad signal of BOH proton is strongly deshielded (13.5 ppm) which points to the formation of a strong intramolecular H-bond with the nitrogen atom of the oxazoline ring. 5a is a much weaker acid (p*K*_a_ = 9.5 in H_2_O/MeOH, 1 : 1) compared to other benzosiloxaboroles.^6^ It should be noted that the acidity of the isomeric compound 3a is relatively high; the p*K*_a_ of 5.8 falls within the range characteristic for various benzosiloxaboroles bearing the formyl group instead of 4,4-dimethyloxazolin-2-yl substituent which would indicate that both substituents exert a similar acidifying effect on the boron atom. The low acidity of 5a can be ascribed to the destabilization of the anionic B(OH)_2_O^−^ group by the adjacent bulky oxazoline ring. Moreover, the neutral form of 5a gains additional stabilization owing to the strong intramolecular hydrogen bond. Accordingly, the theoretical calculations performed at M062X/6-311++G(d,p) level of theory confirms the higher stability of 5a with respect to its 3a isomer (Δ*G*° = 35.3 kJ mol^−1^). This trend is also preserved for the pair of corresponding anions 5a-OH^−^ and 3a-OH^−^, although the free enthalpy difference is smaller (Δ*G*° = 15.3 kJ mol^−1^). Finally, the DFT calculations of the standard free enthalpy of OH^−^ binding to boron centres Δ*G*_OH_° are in line with acidity levels of both isomers as the Δ*G*_OH_° value for 3a is more negative than for 5a (−115.4 *vs.* −99.0 kJ mol^−1^).

**Fig. 4:**
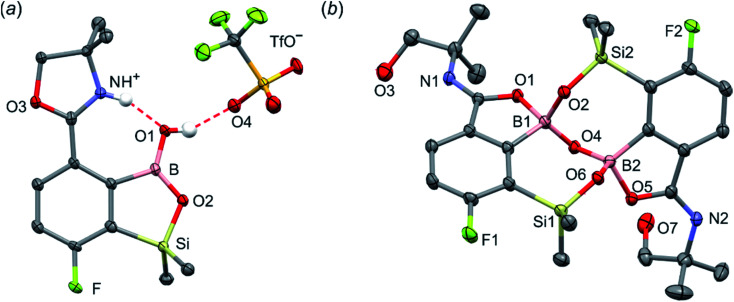
The molecular structures of 5b (a) and 5d (b). Thermal motions given as ADPs at the 50% probability level. C–H hydrogen atoms were omitted for clarity. HBs are marked as red dashed lines.

Unlike the case of 3a, the methylation of oxazoline ring in 5a with MeOTf failed resulting in the isolation of the stable salt 5b with protonated nitrogen atom. Presumably, methylation of oxygen atom of B–OH group is preferred in this case as its nucleophilic character is enhanced due to participation in the aforementioned OH⋯N hydrogen bond. The BOMe moiety in a resulting cationic species is readily hydrolyzed to regenerate BOH group. The structure 5b was confirmed by single crystal X-ray diffraction showing the formation of ion-pair through B–OH⋯^−^OTf hydrogen bond (*d*_O1⋯O4_ = 2.661(3) Å, *d*_H⋯O4_ = 1.87(2) Å, *α*_O1–H⋯O4_ = 155(2)°) as it was observed in case of 3b, and intramolecular hydrogen bond interaction between NH proton and B(OH) group acting as donor and acceptor of HB (Fig. 4a), respectively (*d*_N⋯O1_ = 2.638(5) Å, *d*_H⋯O1_ = 1.81(2) Å, *α*_N–H⋯O1_ = 155(2)°). The facile hydrolytic cleavage of the oxazoline ring in 5a was accomplished by the addition of aq. HCl in acetone giving rise to the ammonium salt 5c, *i.e.*, the analogue of 3g. Unfortunately, subsequent derivatizations with sulfonamide or carboxamide end groups were not effective due to low selectivity. In contrast, 5c underwent a clean intramolecular aminolysis of ester group to give the hydroxyamide derivative 5d. Single crystal X-ray diffraction revealed a unique dimeric structure formed by means of two covalent B–O–Si linkages (the bond angles of 131.3(1)° and 134.2(1)°, Fig. 4b). Thus, the central part of the molecule comprises two seven-membered rings fused through the B–O–B linkage with bond angle of 113.5(1)°. The structure is additionally stabilized by strong dative O–B bonds (*d*_O–B_ = 1.597–1.602 Å) owing to the intramolecular coordination with amide groups. The tetrahedral character of the boron atoms is retained in solution as confirmed by ^10^B NMR chemical shift of 9.9 ppm. It is also worth noting that the signal in the ^29^Si NMR spectrum of 5d is strongly shifted upfield (*δ* = −5.62 ppm) relative to that recorded for 5c (*δ* = 19.22 ppm) which clearly reflects the release of ring strain characteristic for the latter compound.

### Antimicrobial activity

As a part of our ongoing studies on the antimicrobial potential of benzosiloxaboroles and related heterocycles we performed comprehensive screening of activity of obtained compounds towards selected bacteria and fungi strains. The obtained results (Tables S5–S7, ESI†) indicate that the most of tested compounds were moderately active against Gram-positive bacteria including *Staphylococcus aureus* and *Staphylococcus epidermidis*. The only exception were the compounds 3e and 3f that showed high activity against Gram-positive cocci such as methicillin-sensitive *S. aureus* (MSSA) ATCC 6538P, methicillin-resistant *S. aureus* (MRSA) ATCC 43300 as well as the MRSA clinical strains, with the minimal inhibitory concentration (MIC) values in the range of 3.12–6.25 mg L^−1^ (Tables 1 and 2). Moreover, it has been observed that both compounds were potentially active also against *E. faecalis* ATCC 29212 and *Enterococcus faecium* ATCC 6057 (MICs range 25–50 mg L^−1^) (Table 1). Structure–activity relationships for 3d–3f shows that the sulfonamide moiety is mainly responsible for biological activity whilst the additional substitution of the pendant aryl ring with chlorine (3f) did not significantly increase the antimicrobial activity. The MIC values of 3e and 3f were from 16- to 32-fold lower than the MICs of compound 3d for all *S. aureus* strains. Recently, we demonstrated high activity of arylsulfonate derivatives of benzosiloxaboroles against Gram-positive cocci including MRSA ATCC 43300 (MICs in the range of 0.39–6.25 mg L^−1^) and *Enterococcus* spp. (MICs range 6.25–50 mg L^−1^).^5^ As the reference substance for the determination of the activity against Gram-positive cocci we used linezolid (LIN) which belongs to the relatively new group of antibacterial drugs – oxazolidinones. It is used for treatment of severe infections caused by multi-drug resistant cocci including MRSA strains.^45^ The most promising compounds 3e and 3f, showed 3- to 6-fold less activity than LIN against MRSA. It should be emphasized that, according to the CLSI recommendation from 2022, the cut-off points for the MIC values determining the susceptibility of clinical *S. aureus* strains to LIN are ≤4 mg L^−1^ for susceptible strains and ≥8 mg L^−1^ for resistant ones.^46^ Thus, the new compounds 3e and 3f show promising activity against *S. aureus* strains, including MRSA.

**Table 1:** The antibacterial activity of agents 3a and 3d–3f against standard Gram-positive strains^a^

Agent tested	MIC^b^ [MBC], mg L^−1^ (diameter of inhibition zone in mm)					
	*S. aureus* ATCC 6538P MSSA	*S. aureus* ATCC 43300 MRSA	*S. epidermidis* ATCC 12228	*E. faecalis* ATCC 29212	*E. faecium* ATCC 6057	*B. subtilis* ATCC 6633^c^
3a	50 [100] (21)	50 (21)	50 [200] (18)	>400 (—)	>400 (—)	NT (20)
3d	200 [400] (15)	200 [400] (14)	400 (—)	>400 (—)	200 (—)	NT (13)
3e	**6.25** (28)	**6.25** (26)	12.5 (19)	50 (—)	25 (—)	NT (20)
3f	**3.12** [400] (25)	**6.25** (22)	12.5 (16)	25 (13)	25 (15)	NT (22)
LIN^d^	1 [>128] (25)	2 [>128] (25)	1 [>128] (26)	2 [>128] (15)	2 [>128] (14)	NT (30)

a The highest activity against Gram-positive bacteria indicated by the low MIC values (≤6.25 mg L^−1^) is shown in boldface. (—): the inhibition zone was not observed in the disc-diffusion method. The diameter of paper discs was 9 mm.

b Only the MBC values ≤400 mg L^−1^ are presented.

c The growth type of *B. subtilis* in the MHB medium prevented reading the MIC values of tested substances.

d Linezolid (LIN) was used as a reference agent active against Gram-positive bacteria. The diameter of commercial disc containing 0.03 mg of LIN was 6 mm; the MIC of linezolid was determined according to the CLSI recommendations.^47^

**Table 2:** The antibacterial activity of agents 3a and 3d–3f against methicillin-resistant *S. aureus* clinical strains^a^

	MIC^b^ [MBC], mg L^−1^				
	NMI 664K	NMI 1576K	NMI 1712K	NMI 1991K	NMI 2541K
3a	100	100	100	100	100
3d	200	100 [200]	200 [200]	200	100 [400]
3e	**6.25** [*6.25/>400*]^c^	**6.25** [*6.25/>400*]	**6.25** [*25/>400*]	**6.25**	**6.25** [*25/>400*]
3f	**6.25**	**3.12** [*6.25/400*]	**6.25** [*12.5/400*]	**6.25**	**6.25**
LIN^d^	1 [>128]	1 [>128]	1 [>128]	1 [>128]	1 [>128]

a The highest activity against Gram-positive bacteria indicated by the low MIC values (≤6.25 mg L^−1^) is shown in boldface. (—): The inhibition zone was not observed in the disc-diffusion method. The diameter of paper discs was 9 mm.

b Only the MBC values ≤400 mg L^−1^ are presented.

c The Eagle effect was observed during the determination of the MBC value of same tested agents against *S. aureus* strains.^48^ The Eagle effect is shown in italic face.

d Linezolid (LIN) was used as a reference agent active against Gram-positive bacteria. The diameter of commercial disc containing 0.03 mg of LIN was 6 mm; the MIC of LIN was determined according to the CLSI recommendations.^47^

Furthermore, examining the antibacterial activity of new compounds also the minimal bactericidal concentration (MBC) was determined. In the case of 3e and 3f, a paradoxical growth effect, *i.e.*, the two MBC values, was observed for *S. aureus* clinical strains (Table 2). This paradoxical re-increase in the number of observed colonies during the determination of bactericidal activity is called Eagle effect and has been previously reported for several antibiotics.^48^ Some previously reported arylsulfonate-substituted benzosiloxaboroles also showed exhibit this Eagle effect.^5^ The first lowest MBC values of 3e and 3f compounds determined according to CLSI recommendations were 6.25–25 mg L^−1^. However, on the plates with samples taken from the wells containing progressively increasing the agent concentrations (from 2- to 4-fold over the first MBC values), a significant increase in the number of growing colonies, as a paradoxical growth effect, was observed. Finally the second MBC value (≥400 mg L^−1^) was revealed. So far, the causes of the Eagle effect have not yet been fully elucidated in *in vivo* studies in an animal models.^48^ However, there have been reports of therapeutic cases in which a reduction in antibiotic doses resulted in a reduction of bacteria in bloodstream and, consequently, curing patients.^48^

In general, no activity was observed against the Gram-negative rods and yeasts, the MIC values were above the solubility limit of tested compounds (≥400 mg L^−1^, Tables S6 and S7, ESI†). Exceptionally, compound 4d demonstrated the activity against yeasts *Candida* spp. (the MIC value ranges were 3.12–12.5 mg L^−1^) and against Gram-negative bacteria from *Enterobacterales* as well as from Gram-negative non-fermentative rods (MICs 25–100 mg L^−1^). Compound 4d can be regarded as a conjugate of the pseudo-diol 4c and the well-known Tavaborole. Food and Drug Administration (FDA) approved in 2014 Tavaborole (trade name *Kerydin*) for the treatment of onychomycosis – a fungal infection of the nail and nail bed.^2^ In this study, we also observed the activity of Tavaborole against Gram-positive cocci (MICs 12.5–200 mg L^−1^) and non-fermentative Gram-negative rods (MICs 6.25–400 mg L^−1^) and from the order of *Enterobacterales* (MICs 6.25–100 mg L^−1^). Data concerning the following strains: *S. aureus* MSSA, MRSA, *S. epidermidis*, *E. faecalis*, *E. faecium*, *Escherichia coli* and *Pseudomonas aeruginosa*, are in agreement with data presented for Tavaborole in FDA document.^49^ Unfortunately, inspection of antimicrobial activity data for 4c, 4d and Tavaborole (Table S8, ESI†) points to a decrease in activity of 4d against all tested strains of bacteria and yeast compared to Tavaborole which is consistent with the lack of any synergistic effect which might potentially result from combination of two organoboron building blocks.

In the case of Gram-negative rods, one of the most likely causes of resistance to new compounds as well as to the active substances of the drugs used is their active removal through membrane pump systems.^5,6,24,50^ The multidrug (MDR) efflux pumps are widespread in Gram-negative rods. The biggest problem is the resistance–nodulation–division (RND) efflux systems which have a wide substrate range.^51^ To assess the role of efflux systems in resistance of Gram-negative rods to new compounds the RND efflux pump inhibitor, phenylalanine–arginine β-naphthylamide (PAβN), was used.^52^ No significant (at least 4-fold) decrease in the MIC values of the new benzosiloxaborole derivatives tested in the presence of the inhibitor PAβN was observed (Table S6, ESI†). The obtained results firmly confirm the lack of activity of the new compounds against Gram-negative rods.

In addition, cytotoxicity studies were conducted for compounds 3d–3f as well as their precursor 3b. We tested viability of MRC-5 pd30 human fibroblasts after 72 h treatment with the compounds used in the concentration range of 0.78–50 mg L^−1^. The most cytotoxic compound, *i.e.*, 3f reduced viability of cells more than 80%, whereas the least cytotoxic compounds, *i.e.*, 3b and 3d decreased cell viability by about 30% at the highest concentration (for details, see Table S9, ESI†). Moreover, the compounds 3e and 3f were not cytotoxic at the concentration close to their MICs for *S. aureus*, *i.e.*, 3.12–6.25 mg L^−1^.

### Structural insights into the antibacterial activity of benzosiloxaboroles

**Scheme 6:**
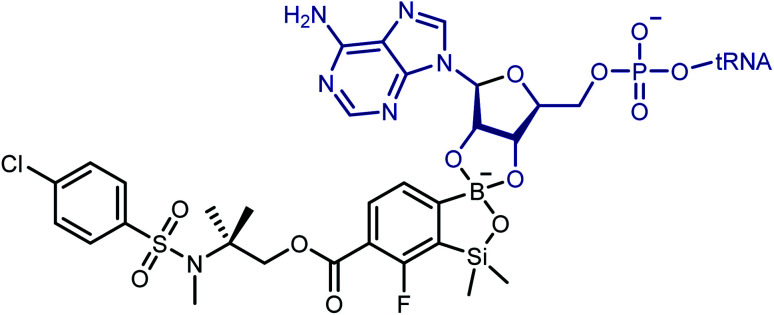
Chemical structure of the covalent adduct between oxaborole and the terminal AMP of tRNA on the example of benzosiloxaborole 3f.

Although promising antibacterial activity results have been obtained for compounds 3e and 3f, the mechanism of their action on the bacteria remains unclear. However, there are more and more reports indicating that molecular target for the benzoxaboroles is leucyl-tRNA synthetase (LeuRS) and the inhibition of this enzyme is based on the oxaborole tRNA-trapping mechanism so-called OBORT mechanism.^53–57^ This mechanism presupposes the formation of a stable tRNA^Leu^–oxaborole adduct in which the boron atom forms two covalent bonds with the 2′- and 3′-oxygen atoms of the ribose of the terminal 3′ tRNA adenosine in the LeuRS editing site (Scheme 6). It leads to trapping of the enzyme-bound tRNA^Leu^ and thus to the prevention of catalytic turnover of leucine and consequently inhibition of protein synthesis.^58–60^ Considering the similarity of benzosiloxaboroles and benzoxaboroles it is tempting to speculate that both compound families share their antibacterial mechanism. In support of this hypothesis, investigated benzosiloxaboroles display rather bacteriostatic than bactericidal character which is in line with the characteristics of the OBORT mechanism. Following this assumption, we modelled the AMP covalent adducts of 3a, 3d–3f, with the putative molecular target, to try and identify properties of the ligand–target complex, that could help us better understand the differences in bacteriostatic activity.

**Fig. 5:**
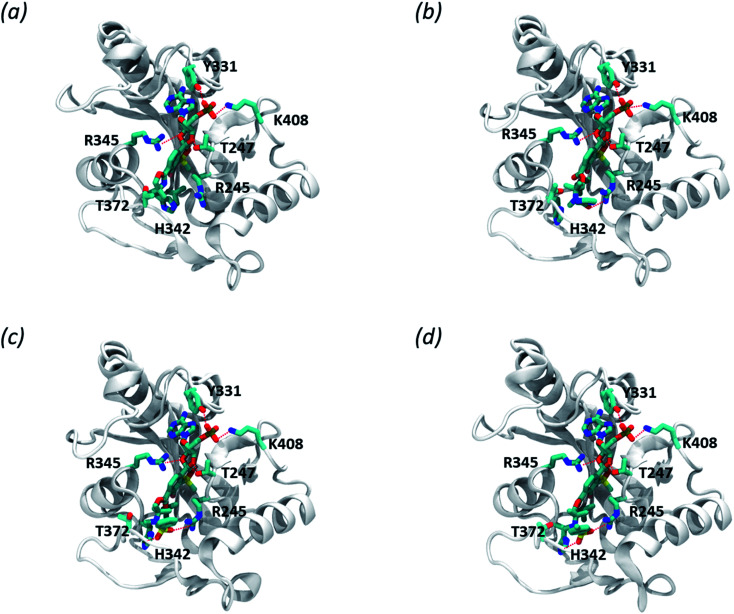
Predicted complexes of benzosiloxaboroles 3a (a), 3d (b), 3e (c), and 3f (d) bound to LeuRS in complex with *S. aureus* MRSA leucyl-tRNA synthetase. Residues contacting the compound are depicted in licorice, polar interactions are depicted as red lines.

We modelled the *S. aureus* MRSA leucyl-tRNA synthetase (*Sa*LeuRS) structure based on the crystal structure of the *T. thermopilus* LeuRS (*Tt*LeuRS) with an adduct of AMP and a closely related antibacterial benzoxaborole locked in the editing site (PDB code: 2V0C).^60^ The sequence of the modelled protein was derived from NCBI GenBank^61,62^ (sequence similarity to template 68.7%). Each ligand was studied as an AMP adduct in order to mimic a terminus of the trapped tRNA which is a common practice for molecular modelling studies concerning the OBORT mechanism.^54,63,64^ Afterwards, the AMP adducts of each of the studied compounds were placed in the enzyme editing site, using coordinates of the crystallized ligand. The resulting complexes were then minimized, using the internal protocol available in MOE.^65^ The obtained complexes allow us to appreciate how in all adducts, the AMP moiety is stabilized by multiple polar interactions with the protein, *i.e.*, T247, Y331, R345, K408 (Fig. 5a–d). Interestingly when comparing compounds 3a and 3d we can appreciate, that within the longer compound the carbonyl group is able to form polar interactions with R245. This additional interaction does not appear to impact the biological activity of this compound, as both compounds 3a and 3d have similar antibacterial potency. In contrast, both compounds 3e and 3f, apart from forming polar interactions with R245, also interact with H342 which is due to presence of a sulfonamide group in their structure. Presumably, an increased number of polar bonds formed by 3e and 3f contributes to the stability of their complexes with LeuRS resulting in higher anti-bacterial activity.

## Conclusions

In conclusion, two isomeric fluorinated benzosiloxaboroles 3a and 5a bearing pendant oxazoline rings were successfully obtained. The synthesis of compound 5a needs careful temperature control after boronation step as a specific merry-go-round intramolecular hydride transfer from silicon through boron to oxazoline C2 atom occurs at higher temperatures eventually leading to a unique structure 4a being the first example of a 7-membered heterocycle with Si–O–B–O–B linkage. It can be readily converted to benzosiloxaborole 4b by simple treatment with dilute aq. HCl. Under the same conditions, the opening of oxazoline ring in 3a and 5a results in derivatives 3b and 5b, respectively. They comprise carboxylic alkyl ester group with attached terminal ammonium functionality. Interestingly, 3a and 5a showed different reactivity towards methyl triflate which is clearly affected by position of oxazoline ring with respect to boronic functionality. Thus, *N*-methylation of 3a occurs cleanly to give the salt 3c which is in contrast with net protonation of 5a. The *N*-methyloxazolinium cation in 3c undergoes readily hydrolytic ring opening which results in compound 3d. Its synthetic utility was demonstrated by *N*-sulfonylation and *N*-acylation to give structurally expanded benzosiloxaboroles 3e–3g. Antimicrobial activity of all new obtained benzosiloxaborole has been investigated in order to gain deeper insight into structure–property relationships for this group of organoboron heterocycles. Compounds 3e and 3f demonstrate relatively high antibacterial activity which shows that the presence of arylsulfonamide motif attached to the benzosiloxaborole core was beneficial. Overall, obtained oxazoline-substituted benzosiloxaboroles and products of their transformations seem to be good candidates for various transformations including conjugation with selected biomolecules which may give rise to novel systems with diverse and potent bioactivity. Further studies in this area will be carried out in our group and the results will be presented in due course.

## Experimental section

### General comments

Solvents used for reactions were dried by heating to reflux with sodium/benzophenone and distilled under argon. Starting materials and other reagents including thionyl chloride, alkyllithiums, diisopropylamine, trimethyl borate, chlorodimethylsilane, were used as received without further purification. In the ^13^C NMR spectra the resonances of boron-bound carbon atoms were not observed in most cases as a result of their broadening by a quadrupolar boron nucleus. ^1^H, and ^13^C NMR chemical shifts are given relative to TMS using residual solvent resonances. ^11^B and ^19^F NMR chemical shifts are given relative to BF_3_·Et_2_O and CFCl_3_, respectively.

### Synthesis

#### *N*-[2-(1-Hydroxy-2-methyl)propyl]-2-fluoro-4-bromobenzamide (1a)

A mixture of 2-fluoro-4-bromobenzoic acid 1 (25.0 g, 0.114 mol) and thionyl chloride (45.0 g, 28 mL, 0.378 mol) was refluxed under an argon atmosphere for 8 h. The excess of SOCl_2_ was removed under reduced pressure to leave crude 2-fluoro-4-bromobenzoyl chloride as a colorless liquid. It was dissolved in dry CH_2_Cl_2_ (100 mL); the solution was cooled in an ice bath followed by a dropwise addition a solution of 2-amino-2-methyl-1-propanol (19.5 g, 0.219 mol) in CH_2_Cl_2_ (100 mL) during 30 min. A white precipitate was formed immediately and the obtained slurry was stirred at ambient temperature for 2 h. It was filtered and washed with CH_2_Cl_2_ (2 × 50 mL). The combined solutions were washed with water (2 × 100 mL) and dried with anhydrous MgSO_4_. The solvent was evaporated under reduced pressure to give 1a (28.4 g, 86%) as a colorless viscous oil. ^1^H NMR (400 MHz, CDCl_3_) *δ* 7.87 (t, *J* = 8.5 Hz, 1H), 7.38 (ddd, *J* = 8.5, 1.8, 0.4 Hz, 1H), 7.28 (dd, *J* = 11.3, 1.8 Hz, 1H), 6.77 (d, *J* = 13.5 Hz, 1H), 4.44 (s, 1H), 3.65 (s, 2H), 1.39 (s, 6H) ppm. ^13^C NMR (101 MHz, CDCl_3_) *δ* 162.9 (d, *J* = 3.7 Hz), 160.0 (d, *J* = 251.0 Hz), 133.1 (d, *J* = 2.6 Hz), 128.5 (d, *J* = 3.4 Hz), 126.5 (d, *J* = 10.5 Hz), 120.7 (d, *J* = 11.5 Hz), 119.7 (d, *J* = 28.2 Hz), 70.3, 56.8, 24.6 ppm. ^19^F NMR (376 MHz, CDCl_3_) *δ* −110.99 (ddd, *J* = 13.6, 11.3, 8.6 Hz) ppm. Anal. calcd for C_11_H_13_BrFNO_2_ (290.13): C, 45.54; H, 4.52; N, 4.83. Found: C, 45.42; H, 4.40; N, 4.80.

#### 2-(2′-Fluoro-4′-bromophenyl)-4,4-dimethyl-2-oxazoline (1b)

Thionyl chloride (33.0 g, 0.277 mol) was added to the neat 1a (28.0 g, 0.097 mol) at 0 °C. The yellow solution was formed in an exothermic reaction; it was stirred at ambient temperature for 6 h. It was diluted with Et_2_O (300 mL) and the resulting slurry was stirred for 1 h at 0 °C. It was filtered and the collected white solid was washed with cold Et_2_O (50 mL) and dissolved in water (200 mL). The obtained clear colorless solution was neutralized by dropwise addition of aqueous 20% NaOH (150 mL) at 0 °C, with stirring. The product was extracted with Et_2_O (2 × 100 mL) and the organic phase was dried over anhydrous MgSO_4_. The solvent was removed under reduced pressure and the liquid residue was subjected to distillation under reduced pressure, to give pure 1b as a colorless viscous oil (b.p. 95–98 °C, *ca.* 1 mbar) which crystallizes on standing to give a white solid, m.p. 38–40 °C. Yield 22.3 g (85%). ^1^H NMR (400 MHz, CDCl_3_) *δ* 7.71 (dd, *J* = 8.6, 7.8 Hz, 1H), 7.31–7.29 (m, 1H), 7.28–7.27 (m, 1H), 4.05 (s, 2H), 1.35 (s, 6H) ppm. ^13^C NMR (101 MHz, CDCl_3_) *δ* 160.8 (d, *J* = 262.5 Hz), 158.3 (d, *J* = 5.5 Hz), 132.2 (d, *J* = 2.6 Hz), 127.4 (d, *J* = 3.8 Hz), 125.8 (d, *J* = 9.2 Hz), 120.3 (d, *J* = 25.2 Hz), 115.6 (d, *J* = 10.7 Hz), 78.9, 68.0, 28.4 ppm. ^19^F NMR (376 MHz, CDCl_3_) *δ* −106.66 (dd, *J* = 9.9, 7.7 Hz) ppm. Anal. calcd for C_11_H_11_BrFNO (272.12): C, 48.55; H, 4.07; N, 5.15. Found: C, 48.52; H, 4.03; N, 5.14.

#### 2-(2′-Fluoro-3′-dimethylsilyl-4′-bromophenyl)-4,4-dimethyl-2-oxazoline (1c)

A solution of 1b (10.9 g, 40.0 mmol) in THF (30 mL) was added dropwise at −75 °C to a stirred solution of LDA, freshly prepared from diisopropylamine (4.55 g, 45.0 mmol) and *n*BuLi (1.6 M, 27.5 mL, 44.0 mmol) in THF (80 mL). After *ca.* 30 min stirring at −78 °C chlorodimethylsilane (4.70 g, 50 mmol) was added slowly and the mixture was stirred for 30 min at −75 °C. It was allowed to warm to room temperature. The resulting almost clear colorless solution was concentrated under reduced pressure. The residue was triturated with heptane (20 mL) and the obtained suspension was filtered in order to remove the solid byproduct LiCl. The filtrate was concentrated and finally subjected to high vacuum (105–110 °C, *ca.* 1 mbar) to give 1c as a pale yellow oil. Yield 11.5 g (87%). ^1^H NMR (400 MHz, CDCl_3_) *δ* 7.72–7.67 (m, 1H), 7.38 (dd, *J* = 8.3, 0.5 Hz, 1H), 4.83–4.75 (m, 1H), 4.08 (s, 2H), 1.39 (s, 6H), 0.46 (dd, *J* = 3.9, 1.9 Hz, 6H) ppm. ^13^C NMR (101 MHz, CDCl_3_) *δ* 165.00 (d, *J* = 256.5 Hz), 158.5 (d, *J* = 6.0 Hz), 133.6 (d, *J* = 12.2 Hz), 133.3 (d, *J* = 3.3 Hz), 129.0 (d, *J* = 3.6 Hz), 127.4 (d, *J* = 33.6 Hz), 115.3 (d, *J* = 15.6 Hz), 78.8, 68.1, 28.4, −3.1 (d, *J* = 4.3 Hz) ppm. ^19^F NMR (376 MHz, CDCl_3_) *δ* −91.47 (dddt, *J* = 7.3, 5.5, 3.8, 1.9 Hz) ppm. Anal. calcd for C_13_H_17_BrFNOSi (330.27): C, 47.28; H, 5.19; N, 4.24. Found: C, 47.22; H, 5.11; N, 4.20.

#### *N*-[2-(1-Hydroxy-2-methyl)propyl]-2-bromo-4-fluorobenzamide (2a)

The synthesis was performed as described for 1a using 2-bromo-4-fluorobenzoic acid (20.0 g, 0.091 mol) and thionyl chloride (36.0 g, 22.5 mL, 0.302 mol). The product was obtained as a white solid, m.p. 97–99 °C. Yield (24.7 g, 93%). ^1^H NMR (400 MHz, CDCl_3_) *δ* 7.48 (dd, *J* = 8.6, 5.9 Hz, 1H), 7.30 (dd, *J* = 8.2, 2.5 Hz, 1H), 7.05 (ddd, *J* = 8.6, 7.8, 2.5 Hz, 1H), 6.30 (s, 1H), 4.39 (broad, 1H), 3.63 (s, 2H), 1.39 (s, 6H) ppm. ^13^C NMR (101 MHz, CDCl_3_) *δ* 167.6, 163.0 (d, *J* = 254.8 Hz), 134.4 (d, *J* = 3.7 Hz), 131.1 (d, *J* = 8.8 Hz), 120.7 (d, *J* = 24.8 Hz), 119.9 (d, *J* = 9.5 Hz), 115.12 (d, *J* = 21.4 Hz), 70.3, 57.3, 24.5 ppm. ^19^F NMR (376 MHz, CDCl_3_) *δ* 108.11 (td, *J* = 8.0, 6.0 Hz) ppm. Anal. calcd for C_11_H_13_BrFNO_2_ (290.13): C, 45.54; H, 4.52; N, 4.83. Found: C, 45.47; H, 4.43; N, 4.77.

#### 2-(2′-Bromo-4′-fluoro)-4,4-dimethyl-2-oxazoline (2b)

The synthesis was performed as described for 1b starting with 2a (24.0 g, 0.083 mol). The product was obtained as a colorless oil (b.p. 94–96 °C, *ca.* 1 mbar). Yield (20.5 g, 91%). ^1^H NMR (400 MHz, CDCl_3_) *δ* 7.62 (dd, *J* = 8.7, 6.0 Hz, 1H), 7.31 (dd, *J* = 8.3, 2.5 Hz, 1H), 7.00 (ddd, *J* = 8.7, 7.9, 2.5 Hz, 1H), 4.07 (s, 2H), 1.35 (s, 6H) ppm. ^13^C NMR (101 MHz, CDCl_3_) *δ* 164.5, 161.4 (d, *J* = 98.9 Hz), 132.8 (d, *J* = 9.1 Hz), 126.6 (d, *J* = 3.5 Hz), 122.6 (d, *J* = 9.6 Hz), 121.1 (d, *J* = 24.5 Hz), 114.5 (d, *J* = 21.3 Hz), 79.4, 68.2, 28.3 ppm. ^19^F NMR (376 MHz, CDCl_3_) *δ* −107.95 (td, *J* = 7.9, 6.0 Hz) ppm. Anal. calcd for C_11_H_11_BrFNO (272.12): C, 48.55; H, 4.07; N, 5.15. Found: C, 48.43; H, 4.00; N, 5.11.

#### 2-(2′-Bromo-3′-dimethylsilyl-4′-fluorophenyl)-4,4-dimethyl-2-oxazoline (2c)

The synthesis was performed as described for 1c starting with 2b (10.9 g, 40 mmol). The product 2c was obtained as a colorless viscous oil (b.p. 108–112 °C, *ca.* 1 mbar). Yield 11.1 g (84%). ^1^H NMR (400 MHz, CDCl_3_) *δ* 7.51 (dd, *J* = 8.5, 6.1 Hz, 1H), 6.97 (t, *J* = 8.3 Hz, 1H), 4.76 (dhept, *J* = 5.7, 3.9 Hz, 1H), 4.10 (s, 2H), 1.38 (s, 6H), 0.43 (dd, *J* = 3.9, 1.9 Hz, 6H) ppm. ^13^C NMR (101 MHz, CDCl_3_) *δ* 167.6 (d, *J* = 249.4 Hz), 161.9, 133.6 (d, *J* = 10.2 Hz), 130.0 (d, *J* = 12.6 Hz), 128.0 (d, *J* = 3.5 Hz), 127.81 (d, *J* = 31.6 Hz), 114.1 (d, *J* = 27.8 Hz), 79.5, 68.2, 28.2, −3.0 (d, *J* = 4.6 Hz) ppm. ^19^F NMR (376 MHz, CDCl_3_) *δ* −92.24 to −92.17 (m) ppm. Anal. calcd for C_13_H_17_BrFNOSi (330.27): C, 47.28; H, 5.19; N, 4.24. Found: C, 47.20; H, 5.07; N, 4.22.

#### 6-(4,4-Dimethyl-2-oxazolin-2-yl)-7-fluoro-1,1-dimethyl-3-hydroxybenzo[1,2,3]siloxaborole (3a)

A solution of 1c (9.9 g, 30 mmol) in Et_2_O (30 mL) was added dropwise to a solution of *t*-BuLi (1.9 M in pentane, 31.5 mL, 60 mmol) in Et_2_O (50 mL) at −90 °C. After 30 min of stirring at −95 °C, B(OMe)_3_ (6.6 mL, 60 mmol) was added slowly to the orange mixture at −90 °C. The resulting suspension was warmed slowly to *ca.* 0 °C, quenched with 1 M aq. NaOH (40 mL) and stirred at room temperature until evolution of H_2_ ceased. The two-phase mixture was concentrated under reduced pressure in order to remove solvents and other volatile organic components. The residual aqueous alkaline solution was transferred to a separatory funnel and washed with Et_2_O (30 mL) and hexane (30 mL). Then it was placed in a beaker, cooled in an ice bath and carefully neutralized by a slow dropwise addition with 1 M aq. H_2_SO_4_ (to reach the pH = 5–6). The precipitated voluminous white solid was filtered and washed several times with distilled water. Then it was resuspended in hexane (30 mL), stirred for 30 min and filtered. Finally, the product was dried *in vacuo* to give 3a as a white powder, m.p. 132–134 °C. Yield 6.68 g (76%). ^1^H NMR (400 MHz, CDCl_3_) *δ* 7.93 (t, *J* = 7.2 Hz, 1H), 7.58 (dd, *J* = 7.5, 1.6 Hz, 1H), 6.62 (broad, 1H), 4.12 (s, 2H), 1.41 (s, 6H), 0.46 (s, 6H) ppm. ^13^C NMR (101 MHz, CDCl_3_) *δ* 162.7 (d, *J* = 255.9 Hz), 159.3 (d, *J* = 5.9 Hz), 136.7 (d, *J* = 32.4 Hz), 133.8, 127.4 (d, *J* = 3.6 Hz), 117.7 (d, *J* = 13.4 Hz), 79.0, 68.1, 28.5, −0.7 ppm. ^19^F NMR (376 MHz, CDCl_3_) *δ* −100.43 to −100.45 (m) ppm. ^10^B NMR (54 MHz, acetone-*d*_6_) *δ* 30.0 ppm. ^29^Si NMR (99.3 MHz, acetone-*d*_6_) *δ* 19.61 ppm. Anal. calcd for C_13_H_17_BFNO_3_Si (293.18): C, 53.26; H, 5.84; N, 4.78. Found: C, 53.12; H, 5.80; N, 4.75. HRMS (ESI, negative ion mode) calcd for C_13_H_16_BFNO_3_Si^−^ [M − H]^−^: 292.0982; found: 292.0984. HRMS (ESI, positive ion mode) calcd for C_13_H_18_BFNO_3_Si^+^ [M + H]^+^: 294.1128; found: 294.1125.

#### 6-(3,4,4-Trimethyl-2-oxazolinium-2-yl)-7-fluoro-1,1-dimethyl-3-hydroxybenzo[1,2,3]siloxaborole trifluoromethanesulfonate (3b)

Methyl trifluoromethanesulfonate (3.1 g, 18.9 mmol) was added dropwise to a stirred solution of 3a (2.05 g, 7.0 mmol) in CHCl_3_ (10 mL). The temperature increased to *ca.* 50 °C during a few minutes indicating the progress of the reaction. Subsequently, a gradual precipitation of a white solid was observed. The suspension was stirred overnight at ambient temperature. Then it was cooled to *ca.* −20 °C and filtered under argon. The solid was washed with cold CHCl_3_ (10 mL) and dried under reduced pressure, to give 3b, m.p. 200–202 °C. Yield 2.65 g (83%). ^1^H NMR (400 MHz, acetone-*d*_6_) *δ* 8.07 (dd, *J* = 7.5, 6.4 Hz, 1H), 7.92 (dd, *J* = 7.5, 1.9 Hz, 1H), 5.17 (s, 2H), 3.52 (d, *J* = 2.3 Hz, 3H), 1.83 (s, 6H), 0.51 (s, 6H) ppm. ^13^C NMR (101 MHz, acetone-*d*_6_) *δ* 169.8 (d, *J* = 2.0 Hz), 162.1 (d, *J* = 255.3 Hz), 154.2 (broad), 137.7 (d, *J* = 31.2 Hz), 135.0, 129.0 (d, *J* = 3.2 Hz), 122.2 (d, *J* = 321.4 Hz), 111.6 (d, *J* = 16.0 Hz), 83.6, 70.0, 31.3 (d, *J* = 5.3 Hz), 23.9, −0.9 ppm. ^19^F NMR (376 MHz, acetone-*d*_6_) *δ* −78.93, −100.22 (dp, *J* = 6.8, 2.3 Hz) ppm. ^10^B NMR (54 MHz, acetone-*d*_6_) *δ* 30.1 ppm. ^29^Si NMR (99.3 MHz, acetone-*d*_6_) *δ* 20.32 ppm.

#### 6-[2-Methyl-2-(*N*-methylammonium)propoxycarbonyl]-7-fluoro-1,1-dimethyl-3-hydroxybenzo[1,2,3]siloxaborole trifluoromethanesulfonate (3c)

Compound 3b (2.7 g, 7 mmol) was dissolved in acetone (20 mL) and water (2 mL, 0.1 mol) was added. The mixture was stirred for 2 h at ambient temperature. Acetone was evaporated under reduced pressure and the residue was dried under high vacuum. DCM (20 mL) was added and the suspension was filtered to give 3c as a white solid, m.p. 161–164 °C. Yield (2.25 g, 80%). ^1^H NMR (400 MHz, DMSO-*d*_6_) *δ* 8.55 (q, *J* = 5.4 Hz, 2H), 8.13 (t, *J* = 7.4 Hz, 1H), 7.76 (dd, *J* = 7.4, 1.5 Hz, 1H), 4.38 (s, 2H), 2.58 (t, *J* = 5.4 Hz, 3H), 1.35 (s, 6H), 0.44 (s, 6H) ppm. ^13^C NMR (101 MHz, DMSO-*d*_6_) *δ* 163.1 (d, *J* = 4.4 Hz), 162.6 (d, *J* = 257.3 Hz), 150.5 (broad), 136.5 (d, *J* = 32.5 Hz), 134.6, 127.7 (d, *J* = 3.5 Hz), 120.7 (q, *J* = 322.2 Hz), 118.7 (d, *J* = 12.1 Hz), 67.0, 57.4, 26.7, 20.3, −0.7 ppm. ^19^F NMR (376 MHz, DMSO-*d*_6_) *δ* −77.79, −101.52 (d, *J* = 7.4 Hz) ppm. Anal. calcd for C_15_H_22_BF_4_NO_7_SSi (475.29): C, 37.91; H, 4.67; N, 2.95; S, 6.75. Found: C, 37.79; H, 4.62; N, 2.85; S, 6.67. HRMS (ESI, positive ion mode) calcd for C_13_H_22_BFNO_3_Si^+^ [M − OTf]^+^: 326.1390; found: 326.1387.

#### 6-[2-Methyl-2-(*N*-methylpyrazinamido)propoxycarbonyl]-7-fluoro-1,1-dimethyl-3-hydroxybenzo[1,2,3]siloxaborole (3d)

To a solution of 3c (293 mg, 1.0 mmol) in MeCN (5 mL) Et_3_N (0.45 mL, 3.5 mmol) and pyrazinoyl chloride^66^ (170 mg, 1.2 mmol) were added. The mixture was heated at 60 °C for 3 h. Then it was evaporated to leave the violet residue. It was dissolved in acetone (5 mL) and water (5 mL) was added. The mixture was concentrated to remove acetone and a white suspension was obtained. The product was isolated as a white powder, m.p. 129–131 °C. Yield 255 mg (59%). ^1^H NMR (400 MHz, CDCl_3_) *δ* 8.83 (d, *J* = 1.6 Hz, 1H), 8.60 (d, *J* = 2.5 Hz, 1H), 8.54 (dd, *J* = 2.5, 1.5 Hz, 1H), 8.09–8.02 (m, 1H), 7.62 (dd, *J* = 7.4, 1.6 Hz, 1H), 5.09 (s, 1H), 4.83 (s, 2H), 3.03 (s, 3H), 1.65 (s, 6H), 0.49 (s, 6H) ppm. ^13^C NMR (101 MHz, CDCl_3_) *δ* 168.5, 164.7 (d, *J* = 4.0 Hz), 163.3 (d, *J* = 257.9 Hz), 151.5, 149.0 (br), 145.0, 144.7, 143.1, 137.2 (d, *J* = 33.2 Hz), 134.9, 127.5 (d, *J* = 3.4 Hz), 119.8 (d, *J* = 12.9 Hz), 69.6, 59.4, 35.5, 24.1, −0.6 ppm. ^19^F NMR (376 MHz, CDCl_3_) *δ* −100.75 ppm. Anal. calcd for C_19_H_23_BFN_3_O_5_Si (431.30): C, 52.91; H, 5.38; N, 9.74. Found: C, 52.87; H, 5.33; N, 9.67. HRMS (ESI, negative ion mode) calcd for C_19_H_22_BFN_3_O_5_Si^−^ [M − H]^−^: 430.1411; found: 430.1412.

#### 6-[2-Methyl-2-(*N*-methylphenylsufonamido)propoxycarbonyl]-7-fluoro-1,1-dimethyl-3-hydroxybenzo[1,2,3]siloxaborole (3e)

To a solution of 2b (190 mg, 0.40 mmol) in MeCN (2 mL) Et_3_N (0.15 mL, 1.0 mmol) and PhSO_2_Cl (88 mg, 0.5 mmol) were added. The mixture was heated at 60 °C for 3 h. Then it was evaporated to leave the viscous colorless residue. It was dissolved in acetone (5 mL) and water (5 mL) was added. The mixture was concentrated to remove acetone and a white suspension was obtained. The solid product 3 was collected by filtration, washed with water (2 × 1 mL) and dried, m.p. 130–132 °C. Yield 140 mg (75%). ^1^H NMR (400 MHz, CDCl_3_) *δ* 7.92 (t, *J* = 7.1 Hz, 1H), 7.85–7.81 (m, 2H), 7.60 (dd, *J* = 7.4, 1.6 Hz, 1H), 7.39 (tt, *J* = 8.8, 6.2 Hz, 3H), 5.05 (s, 1H), 4.52 (s, 2H), 3.05 (s, 3H), 1.50 (s, 8H), 0.51 (s, 6H) ppm. ^13^C NMR (101 MHz, CDCl_3_) *δ* 164.4 (d, *J* = 4.5 Hz), 163.4 (d, *J* = 258.6 Hz), 142.6, 137.2 (d, *J* = 32.9 Hz), 134.9, 132.2, 128.9, 127.4 (d, *J* = 3.7 Hz), 127.0, 119.7 (d, *J* = 12.9 Hz), 71.1, 60.5, 33.2, 25.3, −0.6 ppm. ^19^F NMR (376 MHz, CDCl_3_) *δ* −100.52 (dd, *J* = 7.4, 1.6 Hz) ppm. Anal. calcd for C_20_H_25_BFNO_6_SSi (465.37): C, 51.62; H, 5.41; N, 3.01; S, 6.89. Found: C, 51.40; H, 5.34; N, 2.97; S, 6.67. HRMS (ESI, negative ion mode) calcd for C_20_H_24_BFNO_6_Si^−^ [M − H]^−^: 464.1176; found: 464.1177.

#### 6-{2-Methyl-2-[*N*-methyl-(4-chlorophenyl)sulfonamido]propoxycarbonyl}-7-fluoro-1,1-dimethyl-3-hydroxybenzo[1,2,3]siloxaborole (3f)

The synthesis was performed as described for 3d using 4-chlorophenylsulfonyl chloride. Yield 125 mg (63%). ^1^H NMR (400 MHz, CDCl_3_) *δ* 7.91 (dd, *J* = 7.5, 6.8 Hz, 1H), 7.75 (d, *J* = 8.9 Hz, 1H), 7.61 (dd, *J* = 7.4, 1.7 Hz, 1H), 7.31 (d, *J* = 8.8 Hz, 1H), 4.85 (s, 1H), 4.51 (s, 2H), 3.04 (s, 3H), 1.51 (s, 6H), 0.51 (s, 6H) ppm. ^13^C NMR (101 MHz, CDCl_3_) *δ* 164.4 (d, *J* = 4.1 Hz), 163.3 (d, *J* = 258.3 Hz), 145.6, 141.1, 138.6, 137.3 (d, *J* = 32.8 Hz), 134.8, 129.2, 128.4, 127.5 (d, *J* = 3.7 Hz), 119.5 (d, *J* = 12.8 Hz), 71.0, 60.7, 33.3, 25.4, −0.6 ppm. ^19^F NMR (376 MHz, CDCl_3_) *δ* −100.54 (dd, *J* = 6.8, 1.3 Hz) ppm. Anal. calcd for C_20_H_24_BClFNO_6_SSi (499.82) C, 48.06; H, 4.84; N, 2.80; S, 6.41. Found: C, 47.88; H, 4.79; N, 2.70; S, 6.23. HRMS (ESI, negative ion mode) calcd for C_20_H_23_BClFNO_6_Si^−^ [M − H]^−^: 498.0786; found: 498.0794.

#### 6-[2-Methyl-2-ammoniumpropoxycarbonyl]-7-fluoro-1,1-dimethyl-3-hydroxybenzo[1,2,3]siloxaborole chloride (3g)

Compound 3a (293 mg, 1.0 mmol) was dissolved in acetone (5 mL) followed by addition of 2 M aq. HCl (0.7 mL). The mixture was stirred for 30 min and concentrated under reduced pressure. The residue was triturated with Et_2_O (5 mL) and the obtained suspension was filtered. The solid was washed with Et_2_O (5 mL) and dried to give the product as a white powder, m.p. 220–224 °C. Yield 310 mg (89%). ^1^H NMR (400 MHz, DMSO-*d*_6_) *δ* 8.40 (s, 3H), 8.20 (t, *J* = 7.3 Hz, 1H), 7.78 (dd, *J* = 7.5, 1.6 Hz, 1H), 4.31 (s, 2H), 1.36 (s, 6H), 0.44 (s, 6H) ppm. ^13^C NMR (101 MHz, DMSO-*d*_6_) *δ* 163.1 (d, *J* = 4.2 Hz), 162.6 (d, *J* = 256.0 Hz), 150.3 (broad), 136.3 (d, *J* = 32.3 Hz), 134.6, 127.7 (d, *J* = 3.7 Hz), 118.6 (d, *J* = 11.9 Hz), 68.8, 52.8, 22.5, −0.7 ppm. ^19^F NMR (376 MHz, DMSO-*d*_6_) *δ* −96.95 (d, *J* = 7.1 Hz) ppm. HRMS (ESI, positive ion mode) calcd for C_13_H_20_BFNO_4_Si^+^ [M − Cl]^+^: 312.1233; found: 312.1227.

#### 10-(*tert*-Butyl)-7-fluoro-3,3,8,8-tetramethyl-2,3-dihydro-4*H*,8*H*-1,9,11-trioxa-3*a*-aza-8-sila-10,11*a*,l4-diborabenzo[*ij*]cyclopenta[*c*]azulene (4a)

A solution of 2c (9.9 g, 30 mmol) in Et_2_O (30 mL) was added dropwise to a solution of *t*-BuLi (1.9 M in pentane, 23.5 mL, 45 mmol) in Et_2_O (40 mL) at −90 °C. After 30 min of stirring at −95 °C, B(OMe)_3_ (6.6 mL, 60 mmol) was added slowly to the orange mixture at −90 °C. The resulting suspension was warmed slowly to *ca.* 0 °C, quenched with 1 M aq. NaOH (40 mL) and stirred at room temperature until evolution of H_2_ ceased. The two-phase mixture was concentrated under reduced pressure in order to remove solvents and other volatile organic components. The residual aqueous alkaline suspension was cooled in an ice bath and carefully neutralized by a slow dropwise addition with 1 M aq. H_2_SO_4_ (to reach the pH = 5–6). It was filtered and washed several times with distilled water, hexane (3 × 30 mL) and dried. The crude solid product was mixed with chloroform (50 mL), stirred for 30 min and filtered. The filter cake was washed with chloroform (2 × 20 mL). Combined chloroform solution concentrated under reduced pressure; the residue was mixed with hexane (20 mL) and obtained suspension was filtered, to give 4 as a white powder, m.p. 247–250 °C. Yield 2.92 g (54%). ^1^H NMR (400 MHz, DMSO-*d*_6_) *δ* 7.13 (dd, *J* = 8.3, 5.1 Hz, 1H), 6.85 (t, *J* = 8.6 Hz, 1H), 6.72 (t, *J* = 6.8 Hz, 1H), 4.09 (dd, *J* = 15.8, 5.8 Hz, 1H), 3.99 (dd, *J* = 15.8, 8.0 Hz, 1H), 3.53 (d, *J* = 9.4 Hz, 1H), 3.44 (d, *J* = 9.4 Hz, 1H), 1.53 (s, 3H), 1.24 (s, 3H), 0.85 (s, 9H), 0.35 (d, *J* = 2.8 Hz, 3H), 0.29 (s, 3H) ppm. ^13^C NMR (101 MHz, DMSO-*d*_6_) *δ* 165.1 (d, *J* = 238.1 Hz), 155.6, 136.8 (d, *J* = 1.8 Hz), 125.4 (d, *J* = 8.1 Hz), 124.6 (d, *J* = 27.2 Hz), 113.0 (d, *J* = 27.4 Hz), 73.1, 60.7, 47.5, 27.9, 25.3, 20.7, 2.0 (d, *J* = 4.0 Hz), 1.8 ppm. ^19^F NMR (376 MHz, DMSO-*d*_6_) *δ* −106.06 to −106.11 (m) ppm. ^10^B NMR (54 MHz, DMSO-*d*_6_) *δ* 32.6, 12.7 ppm. ^29^Si NMR (99.3 MHz, DMSO-*d*_6_) *δ* −1.25 ppm. ^1^H NMR (400 MHz, DMSO-*d*_6_ + TfOD/D_2_O) *δ* 7.60 (dd, *J* = 8.3, 5.1 Hz, 1H), 7.26 (dd, *J* = 8.3, 6.9 Hz, 1H), 4.31 (s, 2H), 3.48 (s, 2H), 1.29 (s, 6H), 0.83 (s, 8H), 0.41 (s, 6H) ppm. ^19^F NMR (376 MHz, DMSO-*d*_6_ + TfOD/D_2_O) *δ* −104.26 (t, *J* = 5.6 Hz) ppm. Anal. calcd for C_17_H_28_B_2_FNO_3_Si (363.12): C, 56.23; H, 7.77; N, 3.86. Found: C, 56.04; H, 7.62; N, 3.81. HRMS (ESI, negative ion mode) calcd for C_17_H_27_B_2_FNO_3_Si^−^ [M − H]^−^: 362.1936; found: 362.1943. HRMS (ESI, positive ion mode) calcd for C_17_H_29_B_2_FNO_3_Si^+^ [M + H]^+^: 364.2081; found: 364.2077.

#### *N*-((7-Fluoro-3-hydroxy-1,1-dimethyl-1,3-dihydrobenzo[*c*][1,2,5]oxasilaborol-4-yl)methyl)-1-hydroxy-2-methylpropan-2-aminium chloride (4b)

Compound 4a (0.55 g, 1.5 mmol) was suspended in acetone (5 mL) followed by the addition of 2 M aq. HCl (2 mL). The mixture was stirred for 2 h at 60 °C and the resulting solution was cooled and concentrated under reduced pressure. The residue was triturated with Et_2_O (5 mL) and the obtained suspension was filtered. The solid was washed with Et_2_O (5 mL) and dried to give the product as a white powder, m.p. 234–238 °C (decomp.). Yield 390 mg (78%). ^1^H NMR (500 MHz, DMSO-*d*_6_) *δ* 9.60 (s, 1H), 8.68 (s, 2H), 7.78 (dd, *J* = 8.4, 5.0 Hz, 1H), 7.26 (t, *J* = 7.6 Hz, 1H), 4.39–4.33 (m, 2H), 3.54 (s, 2H), 1.32 (s, 6H), 0.43 (s, 6H) ppm. ^13^C NMR (101 MHz, DMSO-*d*_6_) *δ* 164.3 (d, *J* = 244.0 Hz), 145.6, 136.5 (d, *J* = 6.8 Hz), 134.5 (d, *J* = 31.9 Hz), 133.0 (d, *J* = 2.9 Hz), 116.6 (d, *J* = 24.4 Hz), 65.1, 60.3, 42.3 (d, *J* = 2.4 Hz), 20.6, −0.6 ppm. ^19^F NMR (376 MHz, DMSO-*d*_6_) *δ* −104.47 (t, *J* = 6.1 Hz) ppm. ^10^B NMR (54 MHz, DMSO-*d*_6_) *δ* 29.0 ppm. ^29^Si NMR (99 MHz, DMSO-*d*_6_) *δ* 18.33 ppm. HRMS (ESI, positive ion mode) calcd for C_13_H_22_BFNO_3_Si^+^ [M − Cl]^+^: 298.1441; found: 298.1437.

#### 8-Fluoro-9-(hydroxydimethylsilyl)-3,3-dimethyl-2,3,5-trihydro-10λ4-benzo[3,4][1,2]azaborolo[2,1-*b*][1,3,2]oxazaborol-10-ol (4c)

Compound 4b (333 mg, 1.0 mmol) was treated with the solution of NaHCO_3_ (125 mg, 1.5 mmol) in water (3 mL). The obtained suspension was stirred for 1 h and filtered. The solid was washed with cold water (2 × 2 mL) and dried to give the product as a white powder, m.p. 153–156 °C. Yield 223 mg (75%). ^1^H NMR (400 MHz, DMSO-*d*_6_) *δ* 7.08 (dd, *J* = 8.2, 5.1 Hz, 1H), 6.81 (dd, *J* = 9.3, 8.2 Hz, 1H), 6.14 (t, *J* = 6.0 Hz, 1H), 4.45 (s, 1H), 3.99 (d, *J* = 5.3 Hz, 2H), 3.44 (d, *J* = 9.3 Hz, 1H), 3.11 (d, *J* = 9.3 Hz, 1H), 1.36 (s, 3H), 1.07 (s, 3H), 0.28 (d, *J* = 2.8 Hz, 3H), 0.25 (d, *J* = 1.7 Hz, 3H) ppm. ^13^C NMR (101 MHz, DMSO-*d*_6_) *δ* 165.6 (d, *J* = 236.5 Hz), 137.7 (d, *J* = 1.7 Hz), 126.4 (d, *J* = 26.6 Hz), 124.6 (d, *J* = 8.5 Hz), 113.0 (d, *J* = 28.5 Hz), 72.5, 60.1, 46.8, 25.8, 20.6, 2.73 (d, *J* = 4.6 Hz), 2.69 (d, *J* = 2.8 Hz) ppm. ^10^B NMR (54 MHz, DMSO-*d*_6_) *δ* 12.7 ppm. ^19^F NMR (376 MHz, DMSO-*d*_6_) *δ* −104.36 ppm. ^29^Si NMR (99 MHz, DMSO-*d*_6_) *δ* 2.12 ppm. Anal. calcd for C_13_H_21_BFNO_3_Si (297.20): C, 52.54; H, 7.12; N, 4.71. Found: C, 52.31; H, 6.80; N, 4.65. HRMS (ESI, positive ion mode) calcd for C_13_H_22_BFNO_3_Si^+^ [M + H]^+^: 298.1441; found: 298.1436.

#### (5-Fluoro-2-(7-fluoro-3,3,8,8-tetramethyl-2,3-dihydro-4*H*,8*H*-1,9,11-trioxa-3*a*-aza-8-sila-10,11*a*λ^4^-diborabenzo[*ij*]cyclopenta[*c*]azulen-10-yl)phenyl)methanol (4d)

A mixture of 4c (75 mg, 0.25 mmol) and Tavaborole (38 mg, 0.25 mmol) in acetone (2 mL) was heated at 50 °C for *ca.* 10 min. The resulting solution was concentrated and the residue was triturated with DCM (1 mL) followed by addition of hexane (2 mL). The obtained suspension was cooled and filtered to give 4d as a white solid. Yield 71 mg (66%). ^1^H NMR (400 MHz, DMSO-*d*_6_) ^1^H NMR (400 MHz, acetone-*d*_6_) *δ* 7.77 (dd, *J* = 8.1, 5.7 Hz, 1H), 7.19 (ddt, *J* = 9.4, 1.6, 0.8 Hz, 1H), 7.14–7.06 (m, 2H), 6.79 (dd, *J* = 9.2, 8.3 Hz, 1H), 5.02 (s, 2H), 4.22–4.17 (m, 2H), 3.56 (d, *J* = 9.3 Hz, 1H), 3.22 (d, *J* = 9.3 Hz, 1H), 1.47 (s, 3H), 1.18 (s, 3H), 0.31 (d, *J* = 2.3 Hz, 3H), 0.29 (d, *J* = 2.3 Hz, 3H) ppm. ^19^F NMR (376 MHz, acetone-*d*_6_) *δ* −105.21, −111.89 (td, *J* = 9.4, 5.7 Hz) ppm. ^1^H and ^19^F NMR spectra show additional signals which may indicate some instability of 4d in solution, therefore ^13^C NMR analysis was not performed. The formation of 4d was confirmed by mass spectrometry. HRMS (ESI, positive ion mode) calcd for C_20_H_26_B_2_F_2_NO_4_Si^+^ [M + H]^+^: 432.1780; found: 432.1783.

#### 4-(4,4-Dimethyl-2-oxazolin-2-yl)-7-fluoro-1,1-dimethyl-3-hydroxybenzo[1,2,3]siloxaborole (5a)

A solution of 2c (9.9 g, 30 mmol) in Et_2_O (30 mL) was added dropwise to a solution of *t*-BuLi (1.9 M in pentane, 23.5 mL, 45 mmol) in Et_2_O (40 mL) at −90 °C. After 30 min of stirring at −95 °C, B(OMe)_3_ (5.5 mL, 50 mmol) was added slowly to the orange mixture at −90 °C. The resulting suspension was allowed to warm slowly to *ca.* −70 °C, quenched with 1 M aq. NaOH (40 mL) and stirred at room temperature until evolution of H_2_ ceased. A two-phase mixture was concentrated under reduced pressure in order to remove solvents and other volatile organic components. A residual aqueous alkaline suspension was cooled in an ice bath and carefully neutralized by a slow dropwise addition with 1 M aq. H_2_SO_4_ (to reach the pH = 5–6). It was filtered and washed several times with water. The crude solid product was mixed with hexane (70 mL), stirred for 30 min and filtered; the filter cake was washed with hexane (2 × 30 mL). Combined hexane solution was dried with MgSO_4_ and evaporated to dryness, to give 5a as a white powder, m.p. 67–69 °C. Yield 6.2 g (71%). ^1^H NMR (400 MHz, CDCl_3_) *δ* 12.81 (broad s, 1H), 8.02 (dd, *J* = 8.6, 5.4 Hz, 1H), 7.10 (dd, *J* = 8.6, 6.3 Hz, 1H), 4.16 (s, 2H), 1.43 (s, 6H), 0.47 (s, 6H) ppm. ^13^C NMR (101 MHz, CDCl_3_) *δ* 166.6 (d, *J* = 250.8 Hz), 163.6, 146.2, 137.6 (d, *J* = 33.3 Hz), 134.8 (d, *J* = 7.5 Hz), 126.8 (d, *J* = 3.1 Hz), 116.4 (d, *J* = 24.9 Hz), 79.0, 67.3, 28.4, −0.7 ppm. ^19^F NMR (376 MHz, CDCl_3_) *δ* −98.86 (dd, *J* = 6.3, 5.4 Hz) ppm. ^10^B NMR (54 MHz, acetone-*d*_6_) *δ* 30.1 ppm. ^29^Si NMR (99.3 MHz, acetone-*d*_6_) *δ* 18.35 ppm. Anal. calcd for C_13_H_17_BFNO_3_Si (293.18): C, 53.26; H, 5.84; N, 4.78. Found: C, 53.22; H, 5.70; N, 4.68. HRMS (ESI, negative ion mode) calcd for C_13_H_16_BFNO_3_Si^−^ [M − H]^−^: 292.0982; found: 292.0985.

#### 4-(4,4-Dimethyl-2-oxazolin-2-yl)-7-fluoro-1,1-dimethyl-3-hydroxybenzo[1,2,3]siloxaborole trifluoromethanesulfonate (5b)

The synthesis was performed as described as described for 3b starting with 5a (1.46 g, 5.00 mmol). The product 5b was obtained as a white powder, m.p. 206–209 °C. Yield 1.37 g (62%). ^1^H NMR (400 MHz, acetone-*d*_6_) *δ* 13.16 (s, 1H), 8.37 (dd, *J* = 8.7, 5.2 Hz, 1H), 7.54 (dd, *J* = 8.7, 6.4 Hz, 1H), 5.10 (s, 2H), 1.78 (s, 6H), 0.53 (s, 6H) ppm. ^13^C NMR (101 MHz, acetone-*d*_6_) *δ* 171.2, 169.4 (d, *J* = 255.5 Hz), 139.8 (d, *J* = 34.7 Hz), 138.5 (d, *J* = 9.2 Hz), 122.1 (q, *J* = 321.2 Hz), 121.5, 118.7 (d, *J* = 25.6 Hz), 84.2, 65.1, 26.6, −1.2 ppm. ^19^F NMR (376 MHz, acetone-*d*_6_) *δ* −78.98 (s, OTf^−^), −93.52 (s) ppm. Anal. calcd for C_14_H_18_BF_4_NO_6_SiS (443.25): C, 37.94; H, 4.09; N, 3.16; S, 7.23. Found: C, 37.66; H, 3.98; N, 3.12; S, 7.11.

#### 4-[2-Methyl-2-ammoniumpropoxycarbonyl]-7-fluoro-1,1-dimethyl-3-hydroxybenzo[1,2,3]siloxaborole chloride (5c)

The synthesis was performed as described for 3g using 5a (0.59 g, 2.0 mmol) as a starting material. The product was isolated as a white powder, m.p. 221–224 °C. Yield 0.66 g (95%). ^1^H NMR (400 MHz, DMSO-*d*_6_) *δ* 9.43 (s, 1H), 8.50 (dd, *J* = 8.6, 4.9 Hz, 4H), 7.46–7.39 (m, 2H), 4.36 (s, 2H), 1.38 (s, 6H), 0.44 (s, 6H) ppm. ^13^C NMR (101 MHz, DMSO-*d*_6_) *δ* 168.4, 166.8 (d, *J* = 250.7 Hz), 147.0, 137.5 (d, *J* = 7.9 Hz), 136.7 (d, *J* = 33.0 Hz), 129.4 (d, *J* = 3.2 Hz), 116.9 (d, *J* = 24.9 Hz), 69.7 (d, *J* = 2.1 Hz), 52.7, 22.4, −0.8 ppm. ^19^F NMR (376 MHz, DMSO-*d*_6_) *δ* −97.02 ppm. ^10^B NMR (54 MHz, DMSO-*d*_6_) *δ* 30.0 ppm. ^29^Si NMR (99 MHz, DMSO-*d*_6_) *δ* 19.22 ppm. HRMS (ESI, positive ion mode) calcd for C_13_H_20_BFNO_4_Si^+^ [M − Cl]^+^: 312.1233; found: 312.1227.

#### 5,12-Difluoro-2,9-bis((1-hydroxy-2-methylpropan-2-yl)iminio)-6,6,13,13-tetramethyl-2*H*,6*H*,9*H*,13*H*-1,7,8,14,15-pentaoxa-6,13-disila-7*a*,14*a*-dibora-7*a*,14*a*-methanocyclodeca[1,2,3-*cd*:6,7,8-*c*′*d*′]diindene-7*a*,14*a*-diuide (5d)

Compound 5c (347 mg, 1.0 mmol) was dissolved in methanol (10 mL) followed by the addition of K_2_CO_3_ (276 mg, 2 mmol). The mixture was stirred for 12 h at 70 °C and cooled. 2 M aq. HCl (3 mL) was added and mixture was concentrated. The obtained suspension was filtered; the solid was washed with water (2 × 5 mL) and dried to give the product as a white solid, m.p. 219–222 °C. Yield 274 mg (91%). ^1^H NMR (400 MHz, DMSO-*d*_6_) *δ* 10.27 (s, 1H), 8.52 (dd, *J* = 8.6, 4.7 Hz, 1H), 7.17 (t, *J* = 8.5 Hz, 1H), 3.59 (d, *J* = 11.2 Hz, 1H), 3.56 (d, *J* = 11.2 Hz, 1H), 1.41 (s, 3H), 1.39 (s, 3H), 0.32 (s, 3H), 0.24 (s, 3H) ppm. ^13^C NMR (101 MHz, DMSO-*d*_6_) *δ* 172.0, 169.5 (d, *J* = 249.9 Hz), 165.6, 129.0 (d, *J* = 11.3 Hz), 127.1, 126.3 (d, *J* = 30.7 Hz), 114.7 (d, *J* = 28.0 Hz), 66.6, 59.4 (d, *J* = 2.1 Hz), 23.1, 22.9, 1.83, 1.80 ppm. ^10^B NMR (54 MHz, DMSO-*d*_6_) *δ* 9.9 ppm. ^19^F NMR (376 MHz, DMSO-*d*_6_) *δ* −95.38 ppm. ^29^Si NMR (99 MHz, DMSO-*d*_6_) *δ* −5.62 ppm. Anal. calcd. for C_26_H_36_B_2_F_2_N_2_O_7_Si_2_ (604.36): C, 51.67; H, 6.00; N, 4.64. Found: C, 51.43; H, 5.92; N, 4.64. HRMS (ESI, negative ion mode) calcd for C_26_H_35_B_2_F_2_N_2_O_7_Si_2_^−^ [M − H]^−^: 603.2142; found: 603.2158. HRMS (ESI, positive ion mode) calcd for C_26_H_37_B_2_F_2_N_2_O_7_Si_2_^+^ [M + H]^+^: 605.2288; found: 605.2279.

#### Single crystal X-ray diffraction

Single crystals of all studied systems were prepared by slow solvent evaporation at room temperature from corresponding concentrated CHCl_3_ solutions. Obtained crystals were measured on SuperNova diffractometer equipped with Atlas detector (Cu-K_α_ radiation, *λ* = 1.54184 Å). In all the cases a selected crystal was maintained at low temperature (*T* = 100 K) with the use of Oxford Cryosystems nitrogen gas-flow device. The crystal structures were established in a conventional way *via* X-ray data refinement employing the Independent Atom Model (IAM). Data reduction and analysis were carried out with the *CrysAlisPro* suites of programs.^67^ All structures were solved by direct methods using *SHELXS-97* (ref. 68) and refined using *SHELXL-2016*.^69^ The refinement was based on *F*^2^ for all reflections except those with highly negative values of *F*^2^. Weighted *R* factors (w*R*) and all goodness-of-fit (GooF) values are based on *F*^2^. Conventional *R* factors are based on *F* with *F* set to zero for negative *F*^2^. The *F*_o_^2^ > 2*σ*(*F*_o_^2^) criterion was used only for calculating *R* factors and is not relevant to the choice of reflections for the refinement. All non-hydrogen atoms were refined anisotropically. All carbon-bound hydrogen atoms were placed in calculated positions. The positions of O–H hydrogen atoms were derived from difference electron density maps. The O–H distances were fixed to 0.87 Å with standard deviation of 0.01 Å. All-important crystallographic data including measurement, reduction, structure solution and refinement details are included in Tables S1–S3 (ESI†) or in the associated CIF files. Deposition numbers 2166283 (3a), 2166284 (3b), 2166285 (3d), 2166286 (3e), 2166287 (4a), 2166288 (4c), 2166289 (5b), 2166290 (5c), 2166291 (5d) contain the supplementary crystallographic data for this paper. Additional information on measured crystal structures including packing description, parameters of hydrogen-bond interactions and other relevant parameters can be found in the ESI.†

#### Theoretical calculations

Theoretical calculations were performed using *Gaussian16* program.^70^ Molecules were optimized using M062X^71^ method with 6-311++G(d,p) basis set.^72^ The starting geometries were adopted from corresponding crystal structures or manually modified in the *GaussView*^73^ programme, if crystal data was not available. Following geometry optimization, the vibrational frequencies were calculated and the results showed that optimized structures are stable geometric structures (no imaginary frequencies, Table S4, ESI†). Symmetry constraints were not applied in optimization processes. The standard Gibbs free energies (Δ*G*°) were obtained from the frequency calculations with the temperature set to 298 K. To take into account the experimental conditions all calculations were performed in the presence of the solvent field with the polarizable continuum model (PCM) using the CPCM polarizable conductor calculation model.^74^ The standard Gibbs free energies for the complexation of hydroxyl group (Δ*G*_OH_°) in 3a and 5a were determined for the process A + OH^−^ = AOH^−^.

### Antimicrobial activity

#### Bacterial and fungal strains and their growth conditions

To determine the direct antimicrobial activity the standard and clinical strains were used. The following standard strains were tested: (1) Gram-positive cocci: methicillin-sensitive *Staphylococcus aureus* ATCC 6538P (MSSA), methicillin-resistant *S. aureus* subsp. *aureus* ATCC 43300 (MRSA), *S. epidermidis* ATCC 12228, *Enterococcus faecalis* ATCC 29212, *E. faecium* ATCC 6057, *Bacillus subtilis* ATCC 6633; (2) Gram-negative bacteria from *Enterobacteriales* order: *Escherichia coli* ATCC 25922, *Klebsiella pneumoniae* ATCC 13883, *Proteus mirabilis* ATCC 12453, *Enterobacter cloacae* DSM 6234, *Serratia marcescens* ATCC 13880; (3) Gram-negative non-fermentative rods: *Pseudomonas aeruginosa* ATCC 27853, *Acinetobacter baumannii* ATCC 19606, *Stenotrophomonas maltophilia* ATCC 12714, *S. maltophilia* ATCC 13637, *Burkholderia cepacia* ATCC 25416, *Bordetella bronchiseptica* ATCC 4617; (4) yeasts: *Candida albicans* ATCC 90028, *C. parapsilosis* ATCC 22019, *C. tropicalis* IBA 171, *C. tropicalis* (Castellani) Berkhout ATCC 750, *C. guilliermondii* IBA 155, *C. krusei* ATCC 6258 and *Saccharomyces cerevisiae* ATCC 9763. Moreover, the study was carried out on 5 clinical strains of methicillin-resistant *S. aureus* No. NMI 664K, NMI 1576K, NMI 1712K, NMI 1991K and NMI 2541K. All strains were stored at −80 °C. Prior to testing, each bacterial strain was subcultured twice on tryptic soy agar TSA (bioMerieux) medium and yeast strains on Sabouraud dextrose agar (bioMerieux) for 24–48 h at 30 °C to ensure viability.

#### Determination of antimicrobial activity

Direct antimicrobial activity against yeast, Gram-positive and Gram-negative bacterial strains was examined as previously described^5^ by the disc-diffusion test and the MIC determination assays according to the EUCAST^75,76^ and CLSI^47,77,78^ recommendations. Determination of bactericidal (MBC) and fungicidal (MFC) activity was performed according to the CLSI recommendations.^79,80^ The following reference agents were used: fluconazole (in the case of fungi), linezolid (for Gram-positive bacteria) and nitrofurantoin (for Gram-negative rods). The tested compounds were dissolved in DMSO.

#### Determination of the MICs of compounds in the presence of PAβN

To investigate the contribution of the MDR efflux pumps to the resistance of Gram-negative rods to the new synthesized compounds, the MIC values of studied agents with or without the pump inhibitor PAβN (20 mg L^−1^) (Sigma) were determined.^81^ The MIC determination was performed in Mueller–Hinton II broth medium (MHB) (Becton Dickinson) using 2-fold serial dilutions of tested agents, according to the CLSI guidelines.^47^ In order to minimize the influence of PAβN on destabilization of bacterial cell covers, the tests were conducted in the presence of 1 mM MgSO_4_ (Sigma).^82^ At least a 4-fold decrease in the MIC value after the addition of PAβN was considered significant.^83,84^

#### Cytotoxicity studies

MRC-5 pd30 human fibroblasts (ECACC) were cultured in MEME, Minimum Essential Medium Eagle (Merck) supplemented with 10% fetal bovine serum (Merck), 2 mM l-glutamine, antibiotics (100 U mL^−1^ penicillin, 100 mg L^−1^ streptomycin, Merck) and 1% non-essential amino-acids (Merck). Cells were grown in 75 cm^2^ cell culture flasks (Sarstedt), in a humidified atmosphere of CO_2_/air (5/95%) at 37 °C. MTT-based viability assay was conducted as described previously.^5^ Optical densities were measured at 570 nm using BioTek microplate reader. All measurements were carried out in three replicates and the results expressed as a percent of viable cells *versus* control cells.

#### Structural insights into the antibacterial activity of benzosiloxaboroles

Homology modelling of the *S. aureus* MRSA leucyl-tRNA synthetase (*Sa*LeuRS) editing domain has been done based on the structure of the *T. thermopilus* LeuRS (*Tt*LeuRS) (PDB code: 2V0C). Studied molecules were placed based on the ligand coordinates obtained from the crystal structure. The resulting complexes were then minimized (Amber10:EHT forcefield). All steps were carried out in MOE.^65^ Images were rendered using VMD.^85^

## Conflicts of interest

There are no conflicts of interest to declare.

## Supplementary Material

RA-012-D2RA03910A-s001

RA-012-D2RA03910A-s002

RA-012-D2RA03910A-s003

RA-012-D2RA03910A-s004

RA-012-D2RA03910A-s005

RA-012-D2RA03910A-s006

RA-012-D2RA03910A-s007
