# Supplementary material for: Oxazoline scaffold in synthesis of benzosiloxaboroles and related ring-expanded heterocycles: diverse reactivity, structural peculiarities and antimicrobial activity
Source: RSC Adv. 2022 Aug 16;12(36):23099–117. doi: 10.1039/d2ra03910a (PMC9379557; doi:10.1039/d2ra03910a)
Supplement: RA-012-D2RA03910A-s005 [file RA-012-D2RA03910A-s005.pdf]

Electronic Supplementary Material (ESI) for RSC Advances.
This journal is © The Royal Society of Chemistry 2022

Oxazoline scaffold in synthesis of benzosiloxaboroles and related ring-expanded heterocycles: diverse reactivity and structural peculiarities and antimicrobial activity

Joanna Krajewska,[a] Krzysztof Nowicki,[b] Krzysztof Durka,[b] Paulina H. Marek-Urban,[b] Patrycja Wińska,[b] Tomasz Stępniewski,[c] Krzysztof Woźniak,[d] Agnieszka E. Laudy,[a,*] Sergiusz Luliński[b,*]

[a]*Department of Pharmaceutical Microbiology, Medical University of Warsaw, Oczki 3, 02-007, Warsaw, Poland*

[b]*Warsaw University of Technology, Faculty of Chemistry, Noakowskiego 3, 00-664 Warsaw, Poland*

[c]*GPCR Drug Discovery Lab, Research Programme on Biomedical Informatics (GRIB), Hospital del Mar Medical Research Institute (IMIM) – Department of Experimental and Health Sciences of Pompeu Fabra University (UPF), Carrer del Dr. Aiguader, 88, 08003 Barcelona, Spain*

[d]*University of Warsaw, Faculty of Chemistry, Pasteura 1, 02-093 Warsaw, Poland*

Supporting Information

List of contents

1. **Single-crystal X-ray diffraction……………………………………………….………..…..S2**
2. **Theoretical calculations..…………………………………………………………….…..S8**
3. **Antimicrobial activity………………………………………………..……………..….S11**
4. **NMR spectra……………………………………………………………….…..……..S16**

1. Single-crystal X-ray diffraction

Table S1. Selected crystal data, data collection and refinement parameters for **3a**, **3b**, **3d** and **3e**.

	**3a**	**3b**	**3d**	**3e**
Empirical formula	$C_{13}H_{17}BFNO_3Si$	$C_{15}H_{20}BF_4NO_6SSi$	$C_{19}H_{24}BFN_3O_{5.50}Si$	$C_{20}H_{25}BFNO_6SSi$
Formula weight	293.17	457.28	440.31	465.37
T / K	100.0(1)	100.0(1)	100.0(1)	100.0(1)
Radiation	CuK_α (λ = 1.54178)	CuK_α (λ = 1.54178)	CuK_α (λ = 1.54178)	CuK_α (λ = 1.54178)
Crystal system	Orthorhombic	Monoclinic	Triclinic	Monoclinic
Space group	$P\ 2_12_12_1$	$P\ 2_1/c$	P -1	$P\ 2_1/c$
a / Å	9.7672(3)	2.3262(2)	7.3320(2)	15.9031(3)
b / Å	12.4444(4)	10.8908(2)	10.4076(2)	6.97990(10)
c / Å	12.5674(3)	15.6822(3)	14.5602(4)	20.0486(4)
α / °	90	90	100.665(2)	90
β / °	90	102.680(2)	100.149(2)	99.036(2)
γ / °	90	90	91.918(2)	90
Volume / Å^3	1527.53(8)	2053.87(7)	1072.28(5)	2197.82(7)
Z	4	4	2	4
ρ_{calc} / g·cm^{-3}	1.275	1.479	1.364	1.406
μ / mm^{-1}	1.512	2.596	1.385	2.237
F(000)	616	944	462	976
Crystal size / mm^3	0.159×0.133×0.093	0.249×0.146×0.091	0.197×0.110×0.083	0.423×0.138×0.073
Theta range / °	5.001 to 76.654	3.675 to 76.618	3.143 to 72.668	2.813 to 74.071
Index ranges	–11<=h<=12, –15<=k<=15, –14<=l<=15	–15<=h<=15, –13<=k<=13, –19<=l<=18	–9<=h<=8, –12<=k<=12, –17<=l<=17	–13<=h<=19, –8<=k<=5, –25<=l<=24
Reflections collected	8470	21659	15571	13050
Independent reflections	3081 [R(int) = 0.0184]	4285 [R(int) = 0.0210]	4189 [R(int) = 0.0648]	4390 [R(int) = 0.0246]
Max. and min. transmission	1.000 and 0.851	1.000 and 0.659	1.000 and 0.575	1.000 and 0.752
Data/restraints/parameters	3081 / 1 / 184	4285 / 1 / 265	4189 / 3 / 289	4390 / 1 / 283
Goodness-of-fit on F^2	1.037	1.060	1.052	1.043
Final R indexes [I>=2σ (I)]	R1 = 0.0252, wR2 = 0.0667	R1 = 0.0548, wR2 = 0.1482	R1 = 0.0525, wR2 = 0.1421	R1 = 0.0334, wR2 = 0.0875
Final R indexes [all data]	R1 = 0.0265, wR2 = 0.0677	R1 = 0.0560, wR2 = 0.1496	R1 = 0.0582, wR2 = 0.1490	R1 = 0.0384, wR2 = 0.0927
Largest diff. peak/hole / e·Å^{-3}	0.196 and –0.229	1.001 and –0.527	0.698 and –0.653	0.320 and –0.405

Table S2. Selected crystal data, data collection and refinement parameters for **4a**, **4b** and **4c**.

	**4a**	**4b**	**4c**
Empirical formula	$C_{17}H_{28}B_2FNO_3Si$	$C_{13}H_{22}BClFNO_3Si$	$C_{13}H_{21}BFNO_3Si$
Formula weight	363.11	333.66	297.21
T / K	100.0(1)	100.0(1)	100.0(1)
Radiation	CuK_α (λ = 1.54178)	CuK_α (λ = 1.54178)	CuK_α (λ = 1.54178)
Crystal system	Monoclinic	Monoclinic	Monoclinic
Space group	$P\,2_1/c$	$P\,2_1/c$	$P\,2_1/c$
a / Å	11.5789(3)	11.7424(3)	9.4868(3)
b / Å	9.7201(3)	11.9111(2)	8.2232(2)
c / Å	17.4568(5)	12.6569(3)	18.9873(4)
α / °	90	90	90
β / °	91.799(3)	110.168(2)	98.992(2)
γ / °	90	90	90
Volume / Å^3	1963.76(10)	1661.71(7)	1463.03(7)
Z	4	4	4
ρ_{calc} / g·cm^{-3}	1.228	1.334	1.349
μ / mm^{-1}	1.259	2.893	1.579
F(000)	776	704	632
Crystal size / mm^3	0.333×0.115× 0.066	0.211×0.084× 0.048	0.259×0.200× 0.085
Theta range / °	5.069 to 72.571	4.010 to 76.785	4.716 to 76.679
Index ranges	−14<=h<=10, −7<=k<=11, −21<=l<=16	−14<=h<=14, −14<=k<=10, −13<=l<=15	−11<=h<=11, −8<=k<=10, −23<=l<=20
Reflections collected	7510	7956	16598
Independent reflections	3794 [R(int) = 0.0233]	3445 [R(int) = 0.0310]	3060 [R(int) = 0.0365]
Max. and min. transmission	1.000 and 0.717	1.000 and 0.861	1.000 and 0.744
Data/restraints/parameters	3794 / 1 / 229	3445 / 4 / 202	3060 / 3 / 190
Goodness-of-fit on F^2	0.975	1.070	1.035
Final R indexes [I>=2σ (I)]	R1 = 0.0358, wR2 = 0.0899	R1 = 0.0363, wR2 = 0.0950	R1 = 0.0391, wR2 = 0.1079
Final R indexes [all data]	R1 = 0.0422, wR2 = 0.0950	R1 = 0.0401, wR2 = 0.0995	R1 = 0.0422, wR2 = 0.1112
Largest diff. peak/hole / e·Å^{-3}	0.334 and −0.271	0.385 and −0.286	0.501 and −0.286

Table S3. Selected crystal data, data collection and refinement parameters for **5b** and **5d**.

	**5b**	**5d**
Empirical formula	$C_{14}H_{18}BF_4NO_6SSi$	$C_{29}H_{45}B_2F_2N_2O_{10}Si_2$
Formula weight	443.25	697.47
T / K	100.0(1)	100.0(1)
Radiation	MoK_α (λ = 0.71073)	CuK_α (λ = 1.54178)
Crystal system	Triclinic	Triclinic
Space group	P -1	P -1
a / Å	8.8063(8)	10.1514(2)
b / Å	9.3428(7)	11.8762(3)
c / Å	12.2512(11)	16.5964(3)
α / °	76.873(7)	106.057(2)
β / °	86.633(7)	90.992(2)
γ / °	82.552(7)	114.642(2)
Volume / Å^3	972.90(15)	1727.43(7)
Z	2	2
ρ_{calc} / g·cm^{-3}	1.513	1.341
μ / mm^{-1}	0.296	1.510
F(000)	456	738
Crystal size / mm^3	0.281×0.271× 0.234	0.274×0.209×0.113
Theta range / °	2.255 to 29.697	2.803 to 77.096
Index ranges	–10<=h<=11, –12<=k<=12, –16<=l<=11	–12<=h<=12, –14<=k<=14, –20<=l<=20
Reflections collected	7537	36536
Independent reflections	4855 [R(int) = 0.0240]	7240 [R(int) = 0.0314]
Max. and min. transmission	1.000 and 0.755	1.000 and 0.555
Data/restraints/parameters	4855 / 0 / 254	7240 / 6 / 481
Goodness-of-fit on F^2	1.057	1.023
Final R indexes [I>=2σ (I)]	R1 = 0.0858, wR2 = 0.2664	R1 = 0.0545, wR2 = 0.1521
Final R indexes [all data]	R1 = 0.1043, wR2 = 0.2902	R1 = 0.0569, wR2 = 0.1546
Largest diff. peak/hole / e·Å^{-3}	1.945 and –0.489	0.814 and –0.675

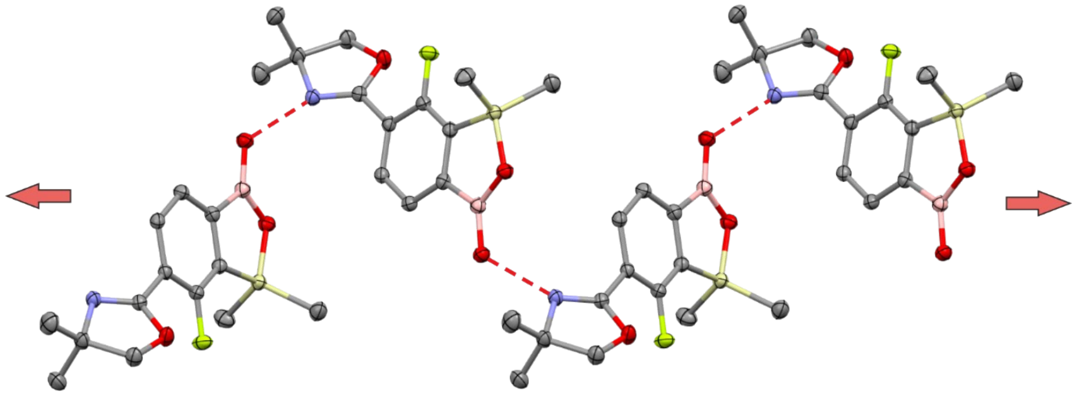

Figure S1. Supramolecular structures of **3a** showing the formation of molecular chains based on O–H…N HB interactions ($d_{O\ldots N}$ = 2.752(2) Å, $d_{H\ldots N}$ = 1.89(2) Å, $\alpha_{O–H\ldots N}$ = 169(2)°). Hydrogen atoms are omitted for clarity.

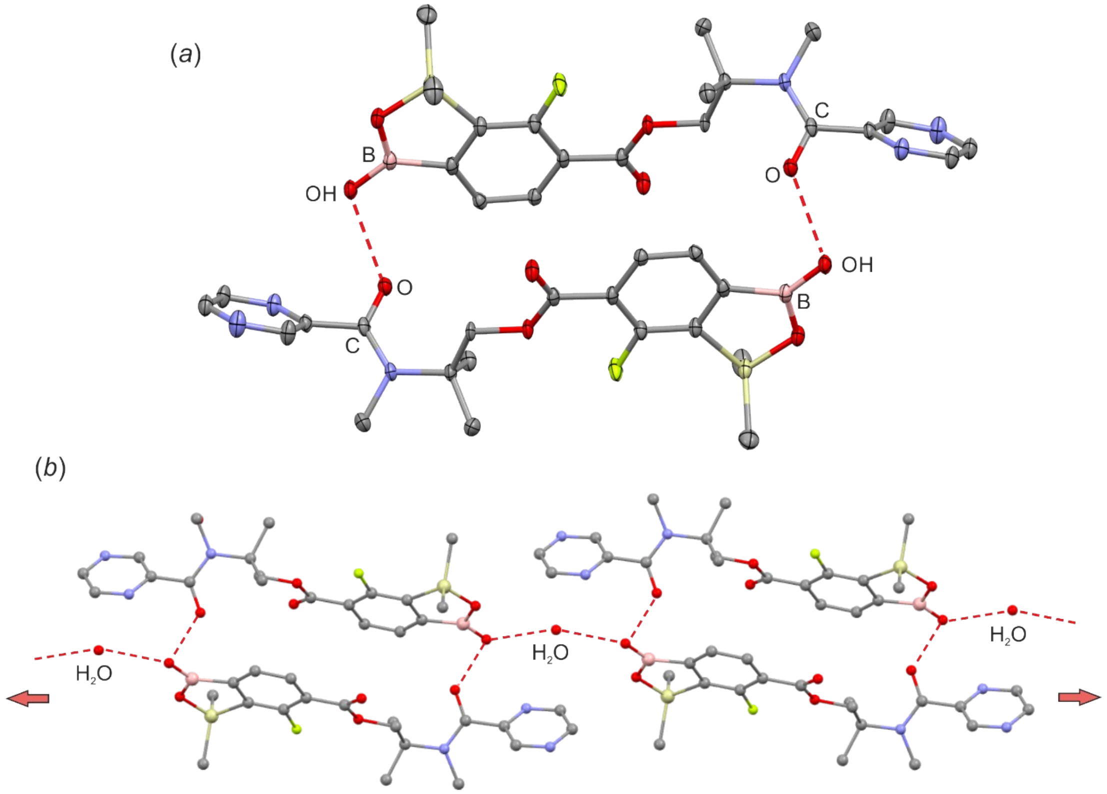

Figure S2. (a) Centrosymmetric dimeric motif based on O-H…O HB interactions between B–OH and C=O moieties ($d_{O\ldots O}$ = 2.684(2) Å, $d_{H\ldots O}$ = 1.84(2) Å, $\alpha_{O–H\ldots O}$ = 167(2)°) and (b) formation of double chain motif through HB interactions with H_2O molecule ($d_{O\ldots O}$ = 2.870(5) Å, $d_{H\ldots O}$ = 2.05(5) Å, $\alpha_{O–H\ldots O}$ = 154(5)°) in crystal structure **3d**. Hydrogen atoms and disordered site of the water molecule are omitted for clarity.

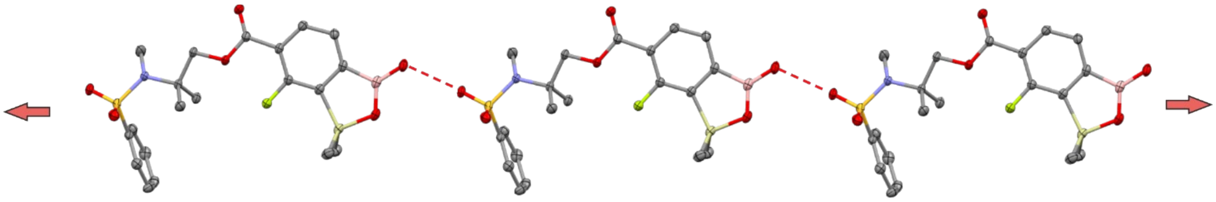

Figure S3. Molecular chain based on O-H…O HB interactions between B-OH and SO_2 groups ($d_{O…N}$ = 2.742(2) Å, $d_{H…N}$ = 1.90(2) Å, $\alpha_{O–H…N}$ = 164(2)°) in crystal structure **3e**. Hydrogen atoms are omitted for clarity.

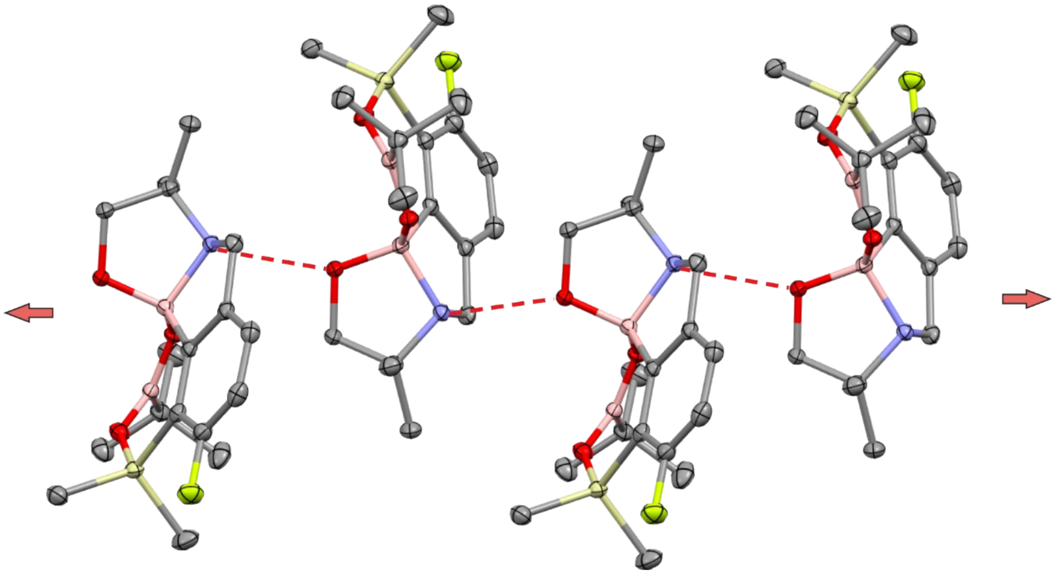

Figure S4. Molecular chain based on charge supported N-H…O HB interactions ($d_{N…O}$ = 2.846(2) Å, $d_{H…O}$ = 2.02(2) Å, $\alpha_{N–H…O}$ = 156(1)°) in crystal structure **4a**. Hydrogen atoms are omitted for clarity.

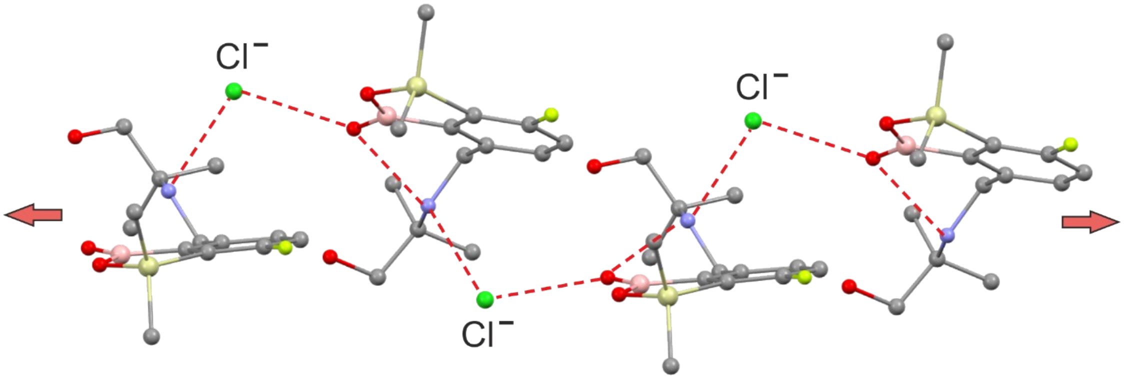

Figure S5. Hydrogen bond molecular chains in structure **4b**: $d_{O1…Cl}$ = 3.074(1) Å, $d_{H…Cl}$ = 2.24(2) Å, $\alpha_{O1–H…Cl}$ = 172(2)°; $d_{O3…Cl}$ = 3.045(1) Å, $d_{H…Cl}$ = 2.21(2) Å, $\alpha_{O3–H…Cl}$ = 177(2)°; $d_{N…Cl}$ = 3.086(1) Å, $d_{H…Cl}$ = 2.24(2) Å, $\alpha_{N–H…Cl}$ = 177(2)°. Hydrogen atoms are omitted for clarity.

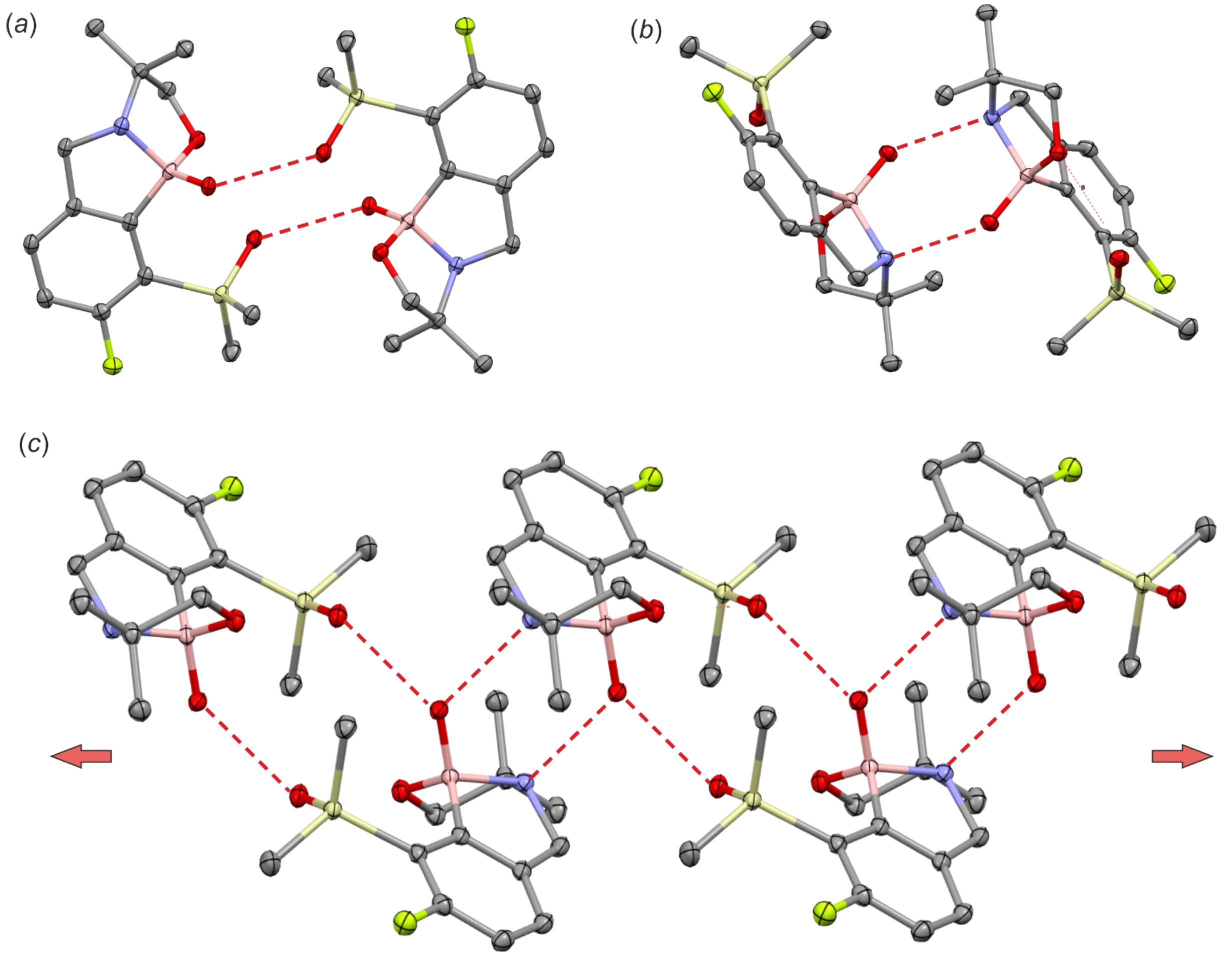

Figure S6. (*a*, *b*) Two types of centrosymmetric hydrogen-bonded dimers ($d_{O...O}$ = 2.873(1) Å, $d_{H...O}$ = 2.04(2) Å, $\alpha_{O–H...O}$ = 163(2)°; $d_{N...O}$ = 2.784(1) Å, $d_{H...O}$ = 1.95(2) Å, $\alpha_{N–H...O}$ = 161(2)°) and (*c*) their propagation into 1D zigzag motif in crystal structure **4c**. Hydrogen atoms are omitted for clarity.

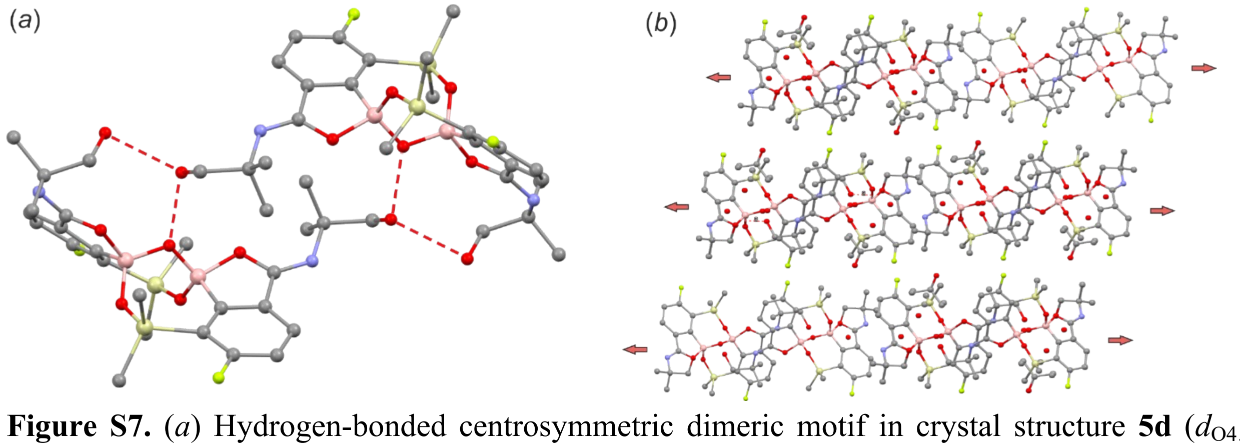

Figure S7. (*a*) Hydrogen-bonded centrosymmetric dimeric motif in crystal structure **5d** ($d_{O4...O3}$ = 2.729(2) Å, $d_{H...O3}$ = 1.98(2) Å, $\alpha_{O4–H...O3}$ = 150(2)°; $d_{O7...O3}$ = 2.754(2) Å, $d_{H...O3}$ = 2.11(2) Å, $\alpha_{O7–H...O3}$ = 170(2)°). (*b*) Molecular HB layers parallel to (011) plane.

2. Theoretical calculations.

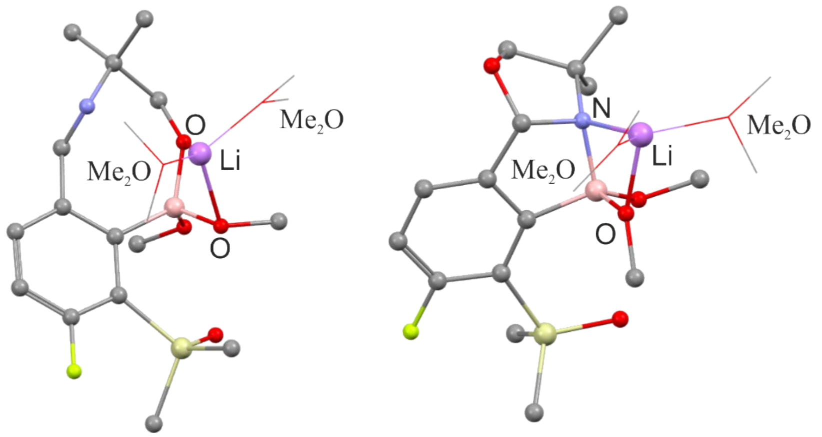

Figure S8. DFT-optimized structures of two proposed reduction intermediates of compound **2c**.

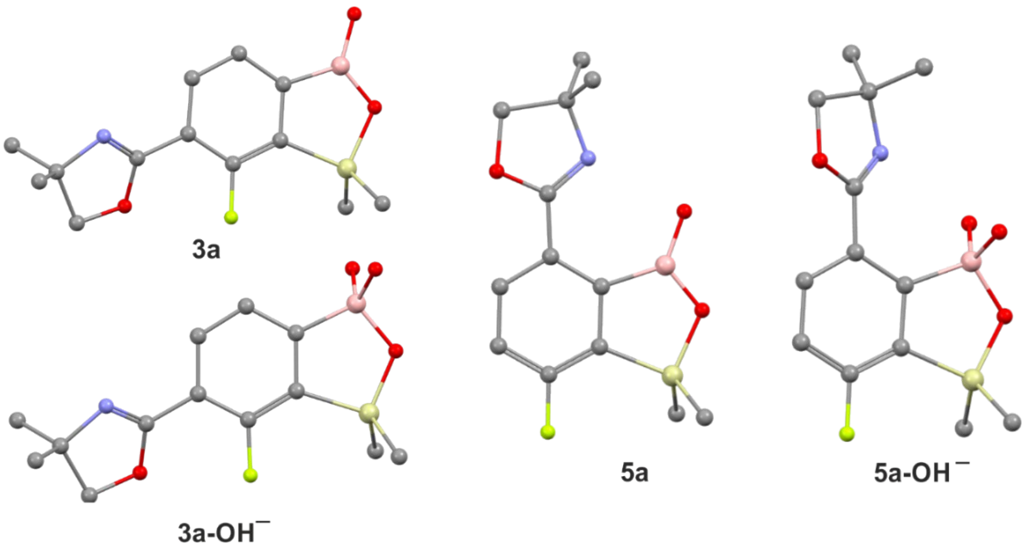

Figure S9. DFT-optimized structures of **3a**, **5a** and their corresponding anions.

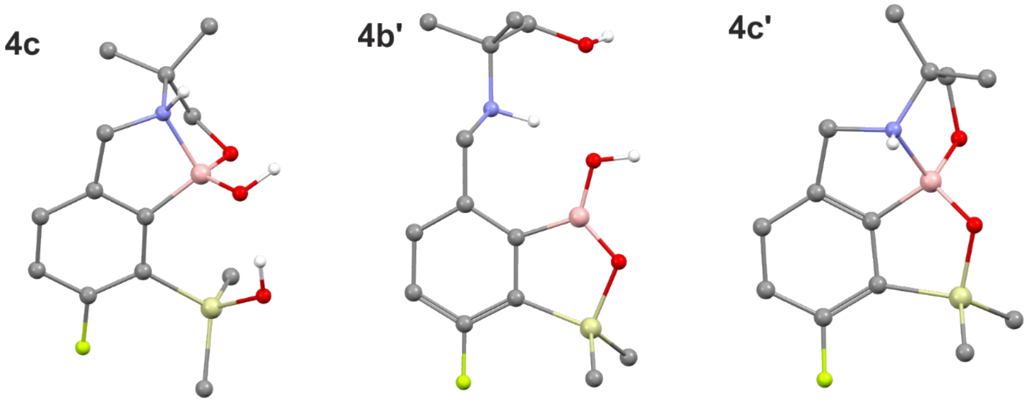

$\Delta\Delta G°$/ kJ·mol^{-1} 0.0 34.2 47.0

Figure S10. DFT-optimized structures **4c**, **4b’** and **4c’** and their relative stabilities.

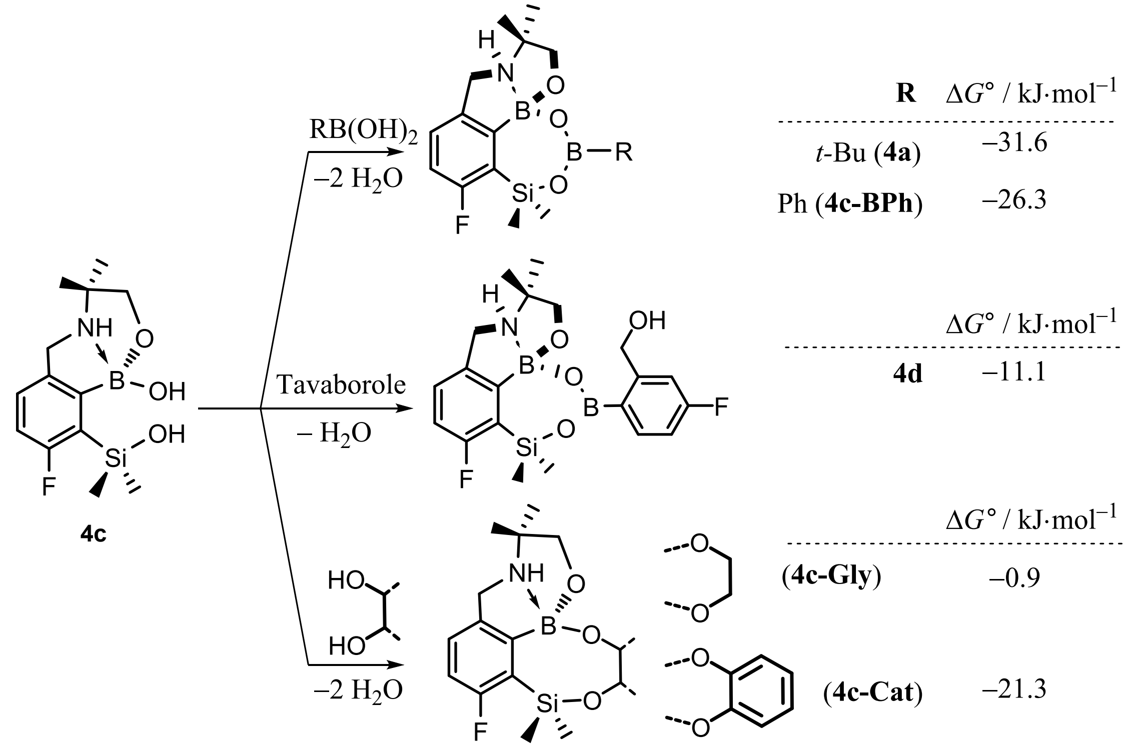

Scheme S1. Trapping of representative alkyl and arylboronic acids, and diols with **4c**. The standard free enthalpy values ΔG° are provided. Calculations were performed at M062X/6-311++G(d,p) level of theory with water solvent field.

Table S4. Computed total energy values and number of imaginary frequencies for the optimized structures (M062X/6-311++G(d,p)).

Compound	*G* / a.u.	No. of imag. freq.
**3a**	–1200.072940	0
**3a-OH$^-$**	–1275.976710	0
**5a**	–1200.083945	0
**5a-OH$^-$**	–1275.968065	0
OH^-	–75.920572	0
H_2O	–76.425173	0
**4c**	–1202.458190	0
**4b'**	–1202.445167	0
**4c'**	–1126.015127	0
**4a**	–1383.926622	0
**4d**	–1671.511597	0
**4c-BPh**	–1457.759959	0
**4c-Gly**	–1432.195216	0
**4c-Cat**	–1279.789454	0
t-BuB(OH)$_2$	–334.306734	0
PhB(OH)$_2$	–408.142102	0
Tavaborole	–545.474357	0
Ethylene Glycol	–230.181258	0
Catechol	–382.579239	0
**2c_oxazolidine**	–1673.075399	0
**2c_imine**	–1673.09268	0

3. Antimicrobial activity.

Table S5. The antibacterial activity of tested agents against standard Gram-positive strains.

**Agent tested**	**MIC in mg/L [MBC in mg/L][a] (Diameter of inhibition zone in mm)**					
	S. aureus **ATCC 6538P**	***S. aureus*** **ATCC 43300 MRSA**	***S. epidermidis*** **ATCC 12228**	***E. faecalis*** **ATCC 29212**	***E. faecium*** **ATCC 6057**	***B. subtilis*** **ATCC 6633[b]**
**3a**	50 [100] (21)	50 (21)	50 [200] (18)	>400 (-)	>400 (-)	NT (20)
**3b**	>400 (-)	>400 (-)	>400 (-)	>400 (-)	>400 (-)	NT (-)
**3d**	200 [400] (15)	200 [400] (14)	400 (-)	>400 (-)	200 (-)	NT (13)
**3e**	**6.25** (28)	**6.25** (26)	12.5 (19)	50 (-)	25 (-)	NT (20)
**3f**	**3.12** [400] (25)	**6.25** [400] (22)	12.5 (16)	25 (13)	25 (15)	NT (22)
**3g**	>400 (-)	>400 (-)	>400 (-)	>400 (-)	>400 (-)	NT (-)
**4a**	>200 (-)	>200 (-)	>200 (-)	>200 (-)	>200 (-)	NT (-)
**4b**	>400 (-)	>400 (-)	>400 (-)	>400 (-)	>400 (-)	NT (-)
**4c**	>100 (-)	>100 (-)	>100 (-)	>100 (-)	>100 (-)	NT (-)
**4d**	50 (19)	100 (19)	50 (28)	400 (-)	>400 (-)	NT (26)
**5a**	>400 (-)	>400 (-)	>400 (-)	>400 (-)	>400 (-)	NT (-)
**5c**	>400 (-)	>400 (-)	>400 (-)	>400 (-)	>400 (-)	NT (-)
**5d**	>400 (-)	>400 (-)	>400 (-)	>400 (-)	>400 (-)	NT (-)
**LIN[c]**	1 [>128] (25)	2 [>128] (25)	1 [>128] (26)	2 [>128] (15)	2 [>128] (14)	NT (30)

The highest activity against Gram-positive bacteria indicated by the low MIC values (≤6.25 mg/L) is shown in boldface.

(-): The inhibition zone was not observed in the disc-diffusion method. The diameter of the paper discs was 9 mm.

[a] Only the MBC values ≤400 mg/L (for **3a–3g**, **4b**, **4d**, **5a**, **5c** and **5d**), ≤200 mg/L (for **4a**) and ≤100 mg/L (for **4c**) are presented. The tested substance **4a** and **4c** dissolved in DMSO precipitated after implementation into the MHB medium at a concentration above 200 mg/L for **4a** and above 100 mg/L for **4c**.

[b] The growth type of *B. subtilis* in the MHB medium prevented reading the MIC values of tested substances.

[c] LIN, linezolid was used as a reference agent active against Gram-positive bacteria. The diameter of commercial disc containing 0.03 mg of linezolid was 6 mm; the MIC of linezolid was determined according to the CLSI recommendations.[1]

Table S6. The antibacterial activity of tested agents against standard Gram-negative strains.

	**MIC in mg/L [MBC in mg/L][a] / x-fold reduction of MIC in the presence of PAβN[b] (Diameter of inhibition zone in mm)**										
**Agent tested**	***E. coli*** **ATCC 25922**	***K. pneumonia*** **ATCC 13883**	***P. mirabilis*** **ATCC 12453**	***E. cloacae*** **DSM 6234**	***S. marcescens*** **ATCC 13880**	***P. aeruginosa*** **ATCC 27853**	***S. maltophilia*** **ATCC 13637**	***S. maltophilia*** **ATCC 12714**	***A. baumannii*** **ATCC 19606**	***B. cepacia*** **ATCC 25416[c]**	***B. bronchiseptica*** **ATCC 4617[c]**
**3a**	>400 (-)	>400 (-)	>400 (-)	>400 (-)	>400 (-)	>400 (-)	>400 (-)	>400 (-)	>400 (-)	>400 (-)	>400 (-)
**3b**	>400 (-)	>400 (-)	>400 (-)	>400 (-)	>400 (-)	>400 (-)	>400 (-)	>400 (-)	>400 (-)	>400 (-)	>400 (-)
**3d**	>400 (-)	>400 (-)	>400 (-)	>400 (-)	>400 (-)	>400 (-)	>400 (-)	>400 (-)	>400 (-)	>400 (-)	>400 (-)
**3e**	>400 (-)	>400 (-)	>400 (-)	>400 (-)	>400 (-)	>400 (-)	>400 (-)	>400 (-)	>400 (-)	>400 (-)	>400 (-)
**3f**	>400 (-)	>400 (-)	>400 (-)	>400 (-)	>400 (-)	>400 (-)	>400 (-)	>400 (-)	>400 (-)	>400 (-)	>400 (-)
**3g**	>400 (-)	>400 (-)	>400 (-)	>400 (-)	>400 (-)	>400 (-)	>400 (-)	>400 (-)	>400 (-)	>400 (-)	>400 (-)
**4a**	>200 (-)	>200 (-)	>200 (-)	>200 (-)	>200 (-)	>200 (-)	>200 (-)	>200 (-)	>200 (-)	>200 (-)	>200 (-)
**4b**	>400 (-)	>400 (-)	>400 (-)	>400 (-)	>400 (-)	>400 (-)	>400 (-)	>400 (-)	>400 (-)	>400 (-)	>400 (-)
**4c**	>100 / 2 (-)	>100 (-)	>100 (-)	>100 (-)	>100 (-)	>100 (-)	>100 (-)	>100 (-)	>100 (-)	>100 (-)	>100 (-)
**4d**	25 (32)	100 (20)	100 (26)	50 (29)	50 (29)	>400 (-)	200 (20)	100 (25)	>400 (-)	>400 (-)	50 (32)
**5a**	>400 (-)	>400 (-)	>400 (-)	>400 (-)	>400 (-)	>400 (-)	>400 (-)	>400 (-)	>400 (-)	>400 (-)	>400 (-)
**5c**	>400 (-)	>400 (-)	>400 (-)	>400 (-)	>400 (-)	>400 (-)	>400 (-)	>400 (-)	>400 (-)	>400 (-)	>400 (-)
**5d**	>400 /2 (-)	>400 (-)	>400 (-)	>400 (-)	>400 (-)	>400 (-)	>400 (-)	>400 (-)	>400 (-)	>400 (-)	>400 (-)
**Nf[d]**	8 [8] (24)	32 [32] (23)	128 [>128] (9)	32 [32] (17)	128 [>128] (12)	>128 [>128] (-)	128 [>128] (-)	128 [>128] (-)	64 [128] (9)	32 [32] (12)	64 [128] (-)

PaβN: efflux pump inhibitor. (-): The inhibition zone was not observed in the disc-diffusion method. The diameter of the paper discs was 9 mm.

[a] Only the MBC values ≤400 mg/L (for **3a–3g**, **4b**, **4d**, **5a**, **5c** and **5d**), ≤200 mg/L (for **4a**) and ≤100 mg/L (for **4c**) are presented. The tested substance **4a** and **4c** dissolved in DMSO precipitated after implementation into the MHB medium at a concentration above 200 mg/L for **4a** and above 100 mg/L for **4c**.

[b] In the table, only at least a 2-fold decrease in the MIC values of tested compounds after the addition of PAβN is presented.

[c] The growth of *B. cepacia* ATCC 25416 and *B. bronchiseptica* ATCC 4617 strains was inhibited in the MHB medium supplemented with 1 mM $MgSO_4$ and 20 mg/L PAβN.

[d] Nf, nitrofurantoin was used as a reference agent active against Gram-negative bacteria. The diameter of commercial disc containing 0.3 mg of nitrofurantoin was 6 mm; the MIC of nitrofurantoin was determined according to the CLSI recommendations.[1]

Table S7. The antifungal activity of tested agents against yeasts strains.

	MIC in mg/L [MFC in mg/L][a] (Diameter of inhibition zone in mm)						
**Agent tested**	***C. albicans* ATCC 90028**	***C. parapsilosis* ATCC 22019**	***C. tropicalis* IBA 171**	***C. tropicalis* ATCC 750**	***C. guilliermondii* IBA 155**	***C. krusei* ATCC 6258**	***S. cerevisiae* ATCC 9763**
**3a**	>400 (-)	>400 (-)	>400 (-)	>400 (-)	>400 (-)	>400 (-)	50 (18)
**3b**	>400 (-)	>400 (-)	>400 (-)	>400 (-)	>400 (-)	>400 (-)	>400 (-)
**3d**	>400 (-)	>400 (-)	>400 (-)	>400 (-)	>400 (-)	>400 (-)	>400 (-)
**3e**	>400 (-)	>400 (-)	>400 (-)	>400 (-)	>400 (-)	>400 (-)	>400 (-)
**3f**	>400 (-)	>400 (-)	>400 (-)	>400 (-)	>400 (-)	>400 (-)	400 (16)
**3g**	>400 (-)	>400 (-)	>400 (-)	>400 (-)	>400 (-)	>400 (-)	>400 (-)
**4a**	>200 (-)	>200 (-)	>200 (-)	>200 (-)	>200 (-)	>200 (-)	>200 (-)
**4b**	>400 (-)	>400 (-)	>400 (-)	>400 (-)	>400 (-)	>400 (-)	>400 (-)
**4c**	>100 (-)	>100 (-)	>100 (-)	>100 (-)	>100 (-)	>100 (-)	>100 (-)
**4d**	3.12 (41)	6.25 (37)	3.12 (33)	3.12 (40)	3.12 (40)	12.5 (25)	0.78 [100] (51)
**5a**	>400 (-)	>400 (-)	>400 (-)	>400 (-)	>400 (-)	>400 (-)	>400 (-)
**5c**	>400 (-)	>400 (-)	>400 (-)	>400 (-)	>400 (-)	>400 (-)	>400 (-)
**5d**	>400 (-)	>400 (-)	>400 (-)	>400 (-)	>400 (-)	>400 (-)	>400 (-)
**Fl[b]**	1 (43)	2 (32)	0.38 (39)	0.38 (40)	0.75 (40)	64[c] (16)	16[d] (12)

(-): The inhibition zone was not observed in the disc-diffusion method. The diameter of paper discs was 9 mm.

[a] Only the MFC values ≤400 mg/L (for **3a–3g**, **4b**, **4d**, **5a**, **5c** and **5d**), ≤200 mg/L (for **4a**) and ≤100 mg/L (for **4c**) are presented. The tested substance **4a** and **4c** dissolved in DMSO precipitated after implementation into the RPMI medium at a concentration above 200 mg/L for **4a** and above 100 mg/L for **4c**. Only the MFC values ≤400 mg/L are presented.

[b] Fl, fluconazole was used as a reference antifungal agent; the diameter of a commercial disc containing 0.025 mg of fluconazole was 6 mm; the MIC value of fluconazole was determined by the Etest method.[2]

[c] The ellipse was visible pointing the MIC value 64 mg/L, however, with macro-colonies up to concentration ≥256 mg/L. In accordance with the recommendations for Etest method, the MIC value of fluconazole against *C. krusei* can be also interpreted as ≥256 mg/L.[2,3] *C. krusei* is intrinsically resistant to fluconazole.

[d] The ellipse was visible pointing the MIC value 16 mg/L, with colonies up to concentration ≥256 mg/L. There are no recommendations for Etest method interpretation of the MIC value of fluconazole against *S. cerevisiae*. The obtained MIC 16 mg/L is in line with the published results.[4]

Table S8. Comparison of activity of **4c**, **4d**, and Tavaborole against standard strains of bacteria and yeasts.

	**MIC in mg/L [MBC/MFC in mg/L][a]/ x-fold reduction of MIC In the presence of PAβN[b] (Diameter of inhibition zone in mm)**		
**Strain**	**4c**	**4d**	**Tavaborole**
S. aureus **ATCC 6538P**	>100 (-)	50 (19)	25 (20)
S. aureus **ATCC 43300 MRSA**	>100 (-)	100 (19)	50 (35)
S. epidermidis **ATCC 12228**	>100 (-)	50 (28)	12.5 [400] (40)
E. faecalis **ATCC 29212**	>100 (-)	400 (-)	100 (20)
E. faecium **ATCC 6057**	>100 (-)	>400 (-)	200 (-)
B. subtilis **ATCC 6633[c]**	NT (-)	NT (26)	NT (35)
E. coli **ATCC 25922**	>100/2 (-)	25 (32)	6.25 (37)
K. pneumoniae **ATCC 13883**	>100 (-)	100 (20)	12.5 (33)
P. mirabilis **ATCC 12453**	>100 (-)	100 (26)	100 (22)
E. cloacae **DSM 6234**	>100 (-)	50 (29)	12.5 (29)
S. marcescens **ATCC 13880**	>100 (-)	50 (29)	25 (36)
P. aeruginosa **ATCC 27853**	>100 (-)	>400 (-)	400 (13)
S. maltophilia **ATCC 13637**	>100 (-)	200 (20)	100 (19)
S. maltophilia **ATCC 12714**	>100 (-)	100 (25)	100 (19)
A. baumannii **ATCC 19606**	>100 (-)	>400 (-)	6.25 (30)
B. cepacia **ATCC 25416[d]**	>100 (-)	>400 (-)	400 (14)
B. bronchiseptica **ATCC 4617[d]**	>100 (-)	50 (32)	12.5 (32)
C. albicans **ATCC 90028**	>100 (-)	3.12 (41)	0.78 (63)
C. parapsilosis **ATCC 22019**	>100 (-)	6.25 (37)	1.56 (59)
C. tropicalis **IBA 171**	>100 (-)	3.12 (33)	1.56 (48)
C. tropicalis **ATCC 750**	>100 (-)	3.12 (40)	1.56 (60)
C. guilliermondii **IBA 155**	>100 (-)	3.12 (40)	1.56 (64)
C. krusei **ATCC 6258**	>100 (-)	12.5 (25)	3.12 (45)
S. cerevisiae **ATCC 9763**	>100 (-)	0.78 [100] (51)	0.39 (74)

PAβN, efflux pump inhibitor. (-) The inhibition zone was not observed in the disc-diffusion method. The diameter of paper discs was 9 mm.

[a] Only the MBC/MFC values ≤400 mg/L (for **4d** and Tavaborole) and ≤100 mg/L (for **4c**) are presented. The tested substance **4c** dissolved in DMSO precipitated after implementation into the MHB and RPMI medium at a concentration above 100 mg/L. [b] The growth type of *B. subtilis* in the MHB medium prevented reading the MIC values of tested substances. [c] In the table, only at least a 2-fold decrease in the MIC values of tested compounds after the addition of PAβN are presented. [d] The growth of *B. cepacia* ATCC 25416 and *B. bronchiseptica* ATCC 4617 strains was inhibited in the MHB medium supplemented with 1 mM $MgSO_4$ and 20 mg/L PAβN.

Table S9. Viability of MRC-5 cells treated with compounds **3b**, **3d–3f** determined by the MTT test after 72 h of incubation.

**Concentration of compound [mg/L]**	**Viability of MRC-5 [% of control ± SD]**			
	**3b**	**3d**	**3e**	**3f**
0.78125	103.8±4.1	108.7±3.2	116.9±5.9	120.3±10.0
1.5625	96.5±2.2	106.3±5.1	115.2±11.4	115.5±3.0
3.125	98.8±6.5	107.7±7.8	113.3±15.4	124.6±2.7
6.25	96.4±4.3	107.5±4.3	111.5±4.0	97.5±5.6
12.5	93.3±3.8	110.1±4.8	102.3±5.1	70.3±3.5
25	83.5±2.0	90.2±7.9	76.6±1.9	30.7±1.4
50	70.9±1.1	67.5±2.5	30.6±5.8	17.3±1.2

4. ^{1}H and ^{13}C NMR spectra

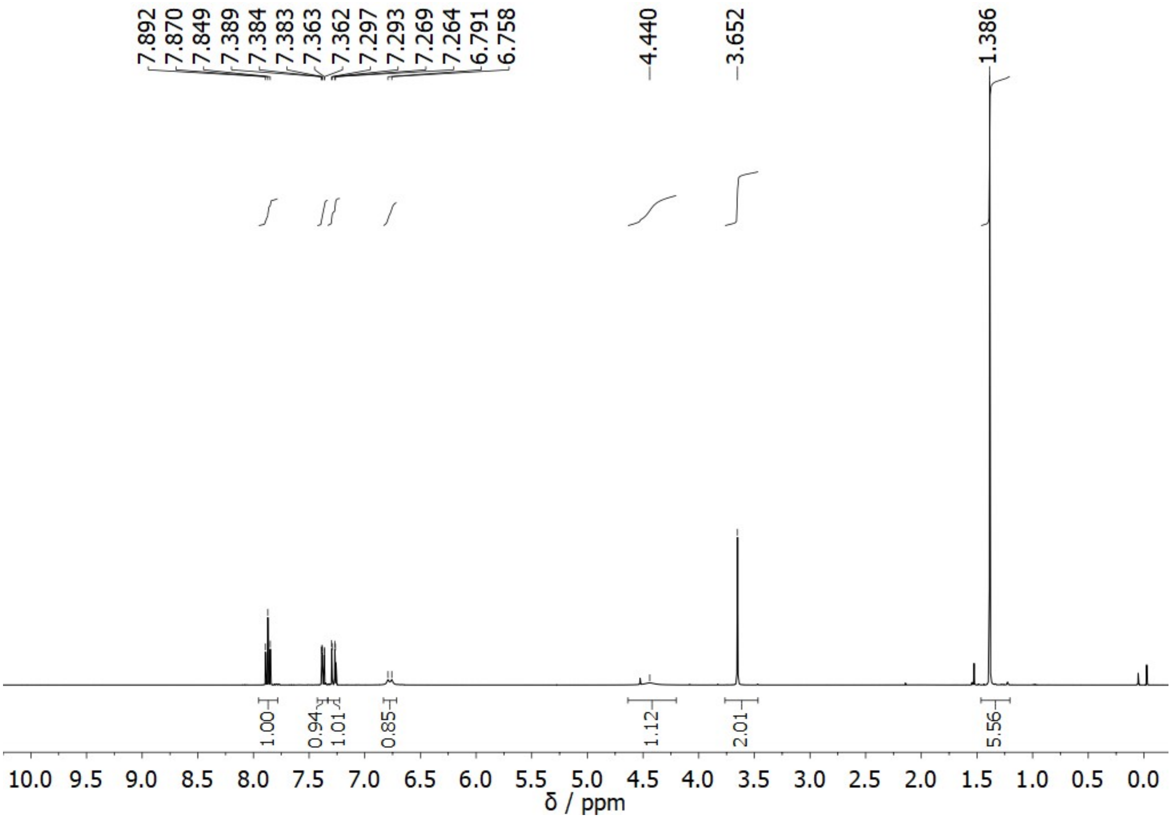

^{1}H NMR spectrum of **1a**.

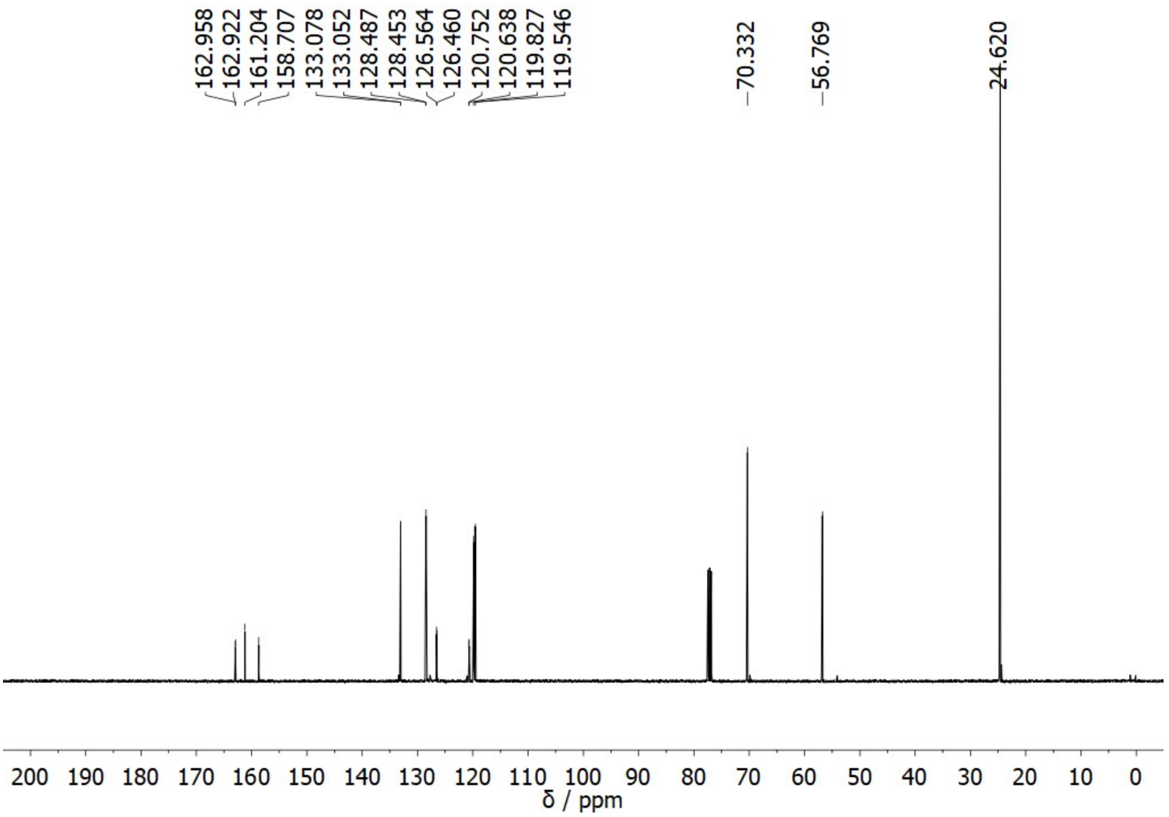

^{13}C NMR spectrum of **1a**.

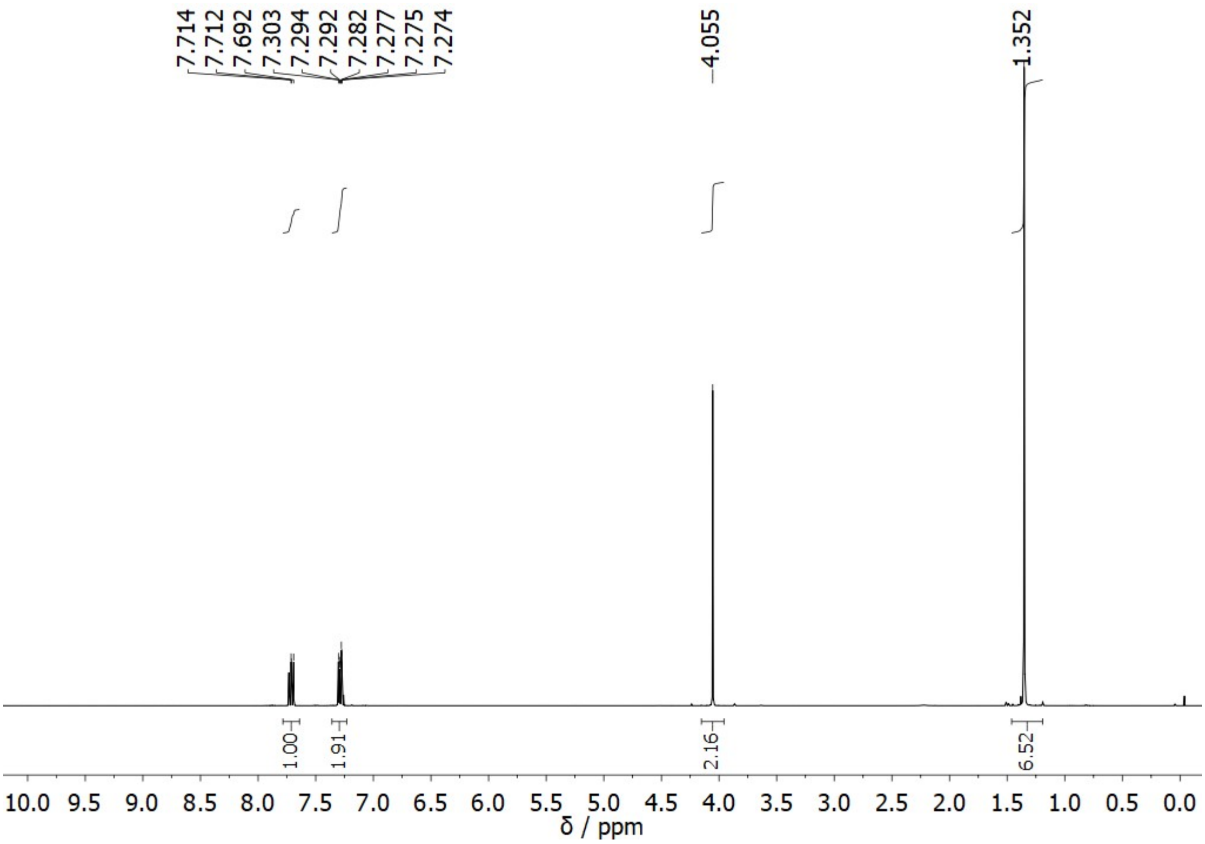

^{1}H NMR spectrum of **1b**.

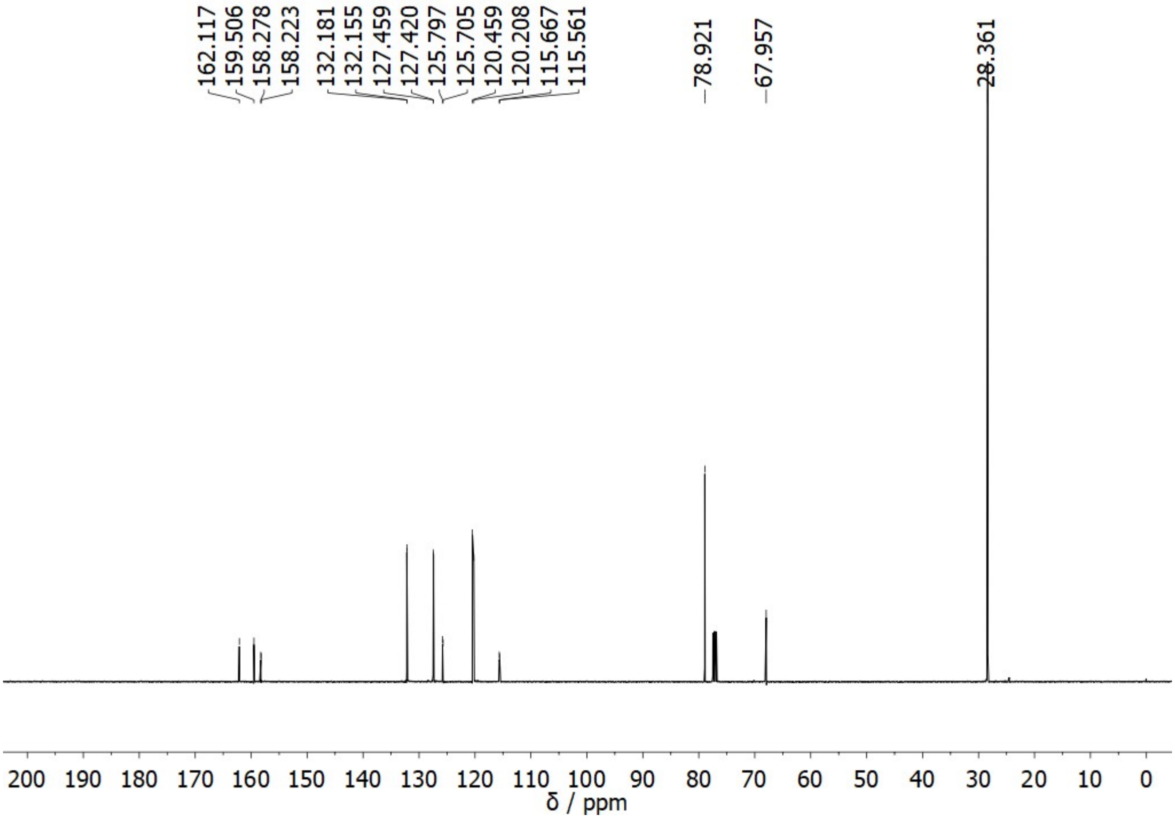

^{1}H NMR spectrum of **1b**.

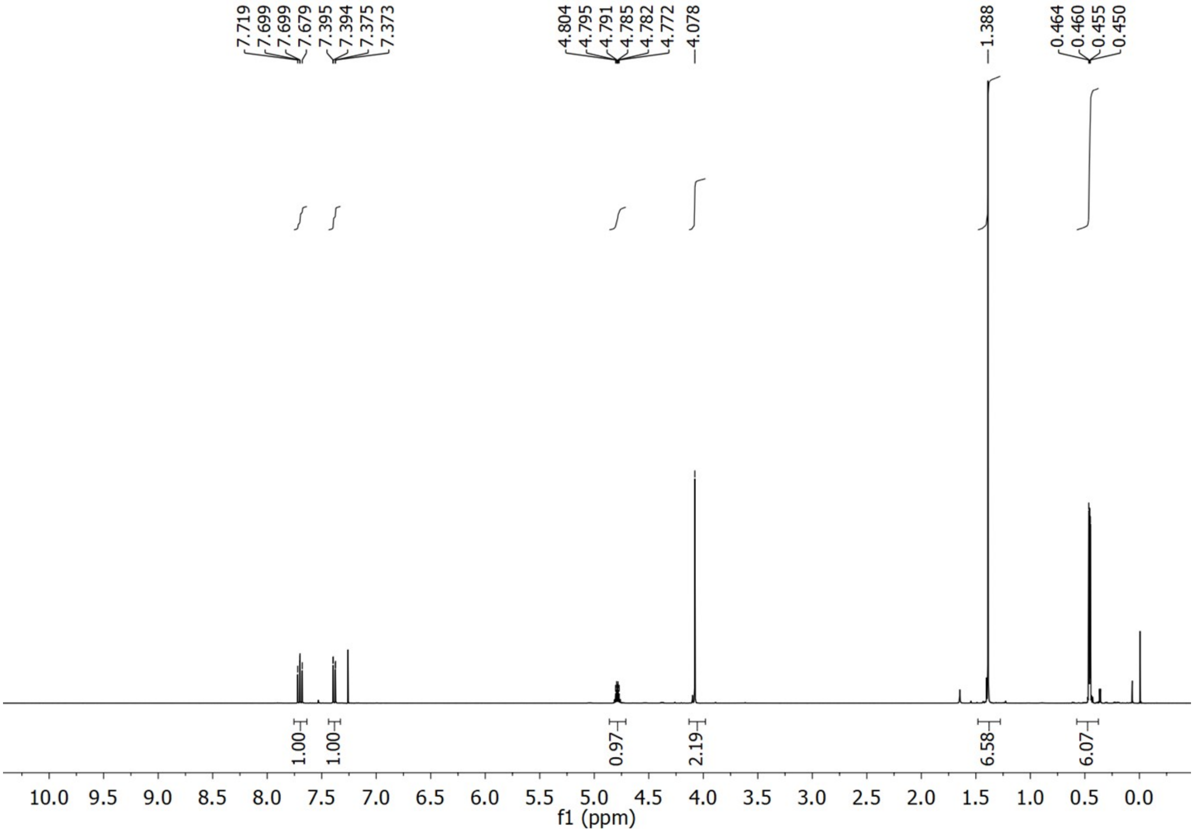

^{1}H NMR spectrum of **1c**.

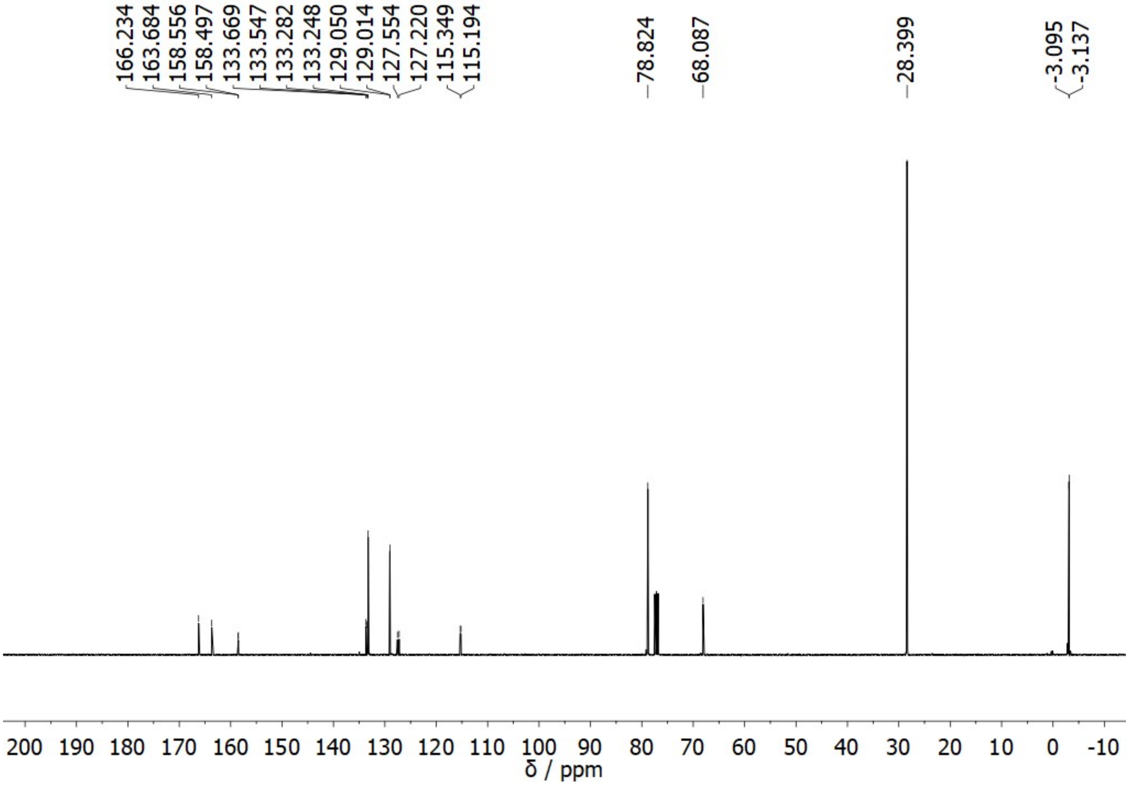

^{13}C NMR spectrum of **1c**.

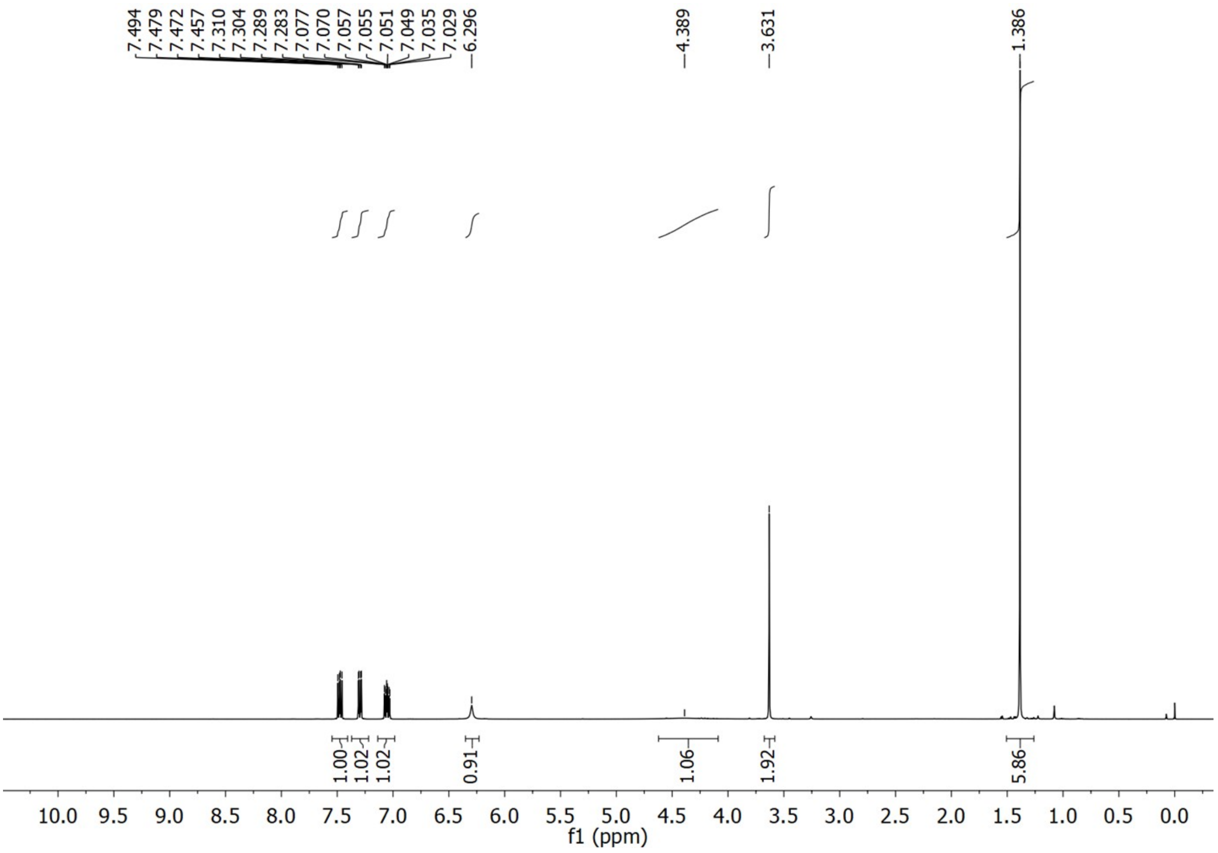

^{1}H NMR spectrum of **2a**.

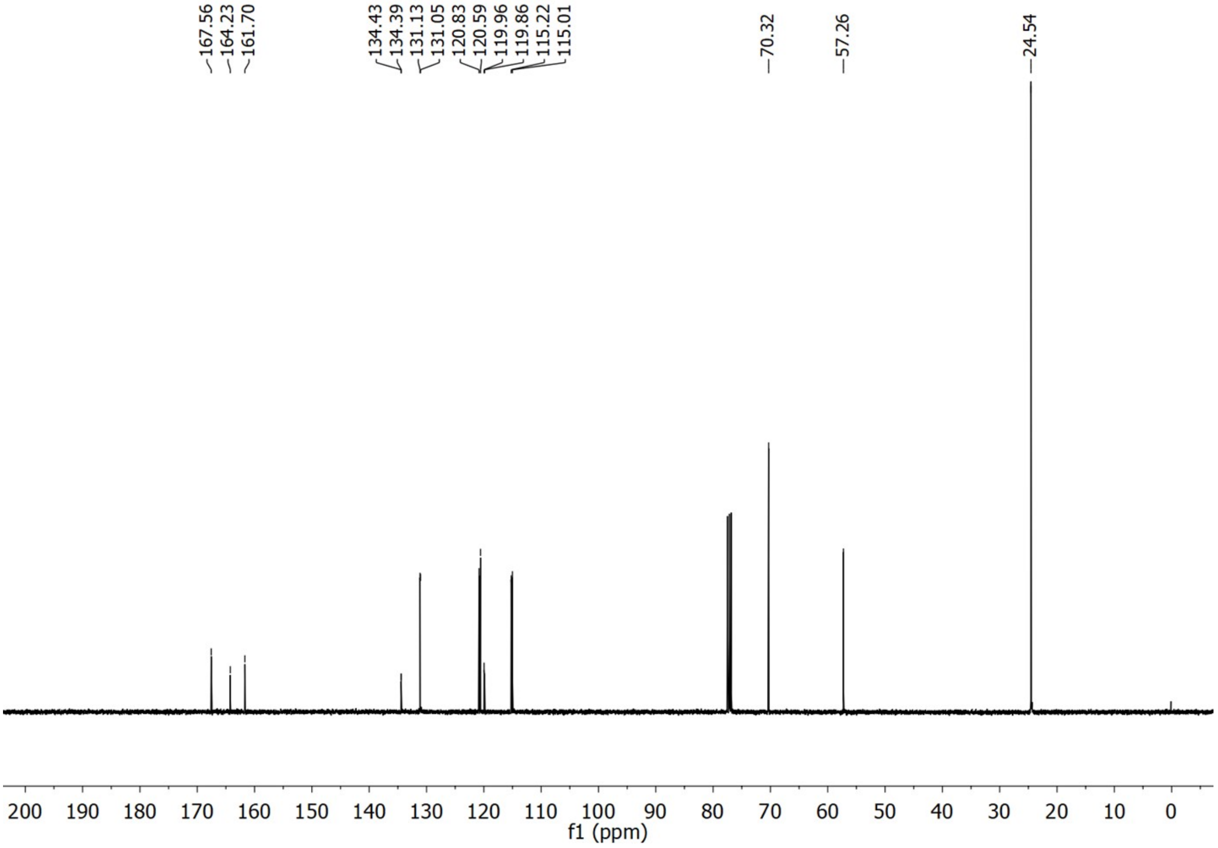

^{13}C NMR spectrum of **2a**.

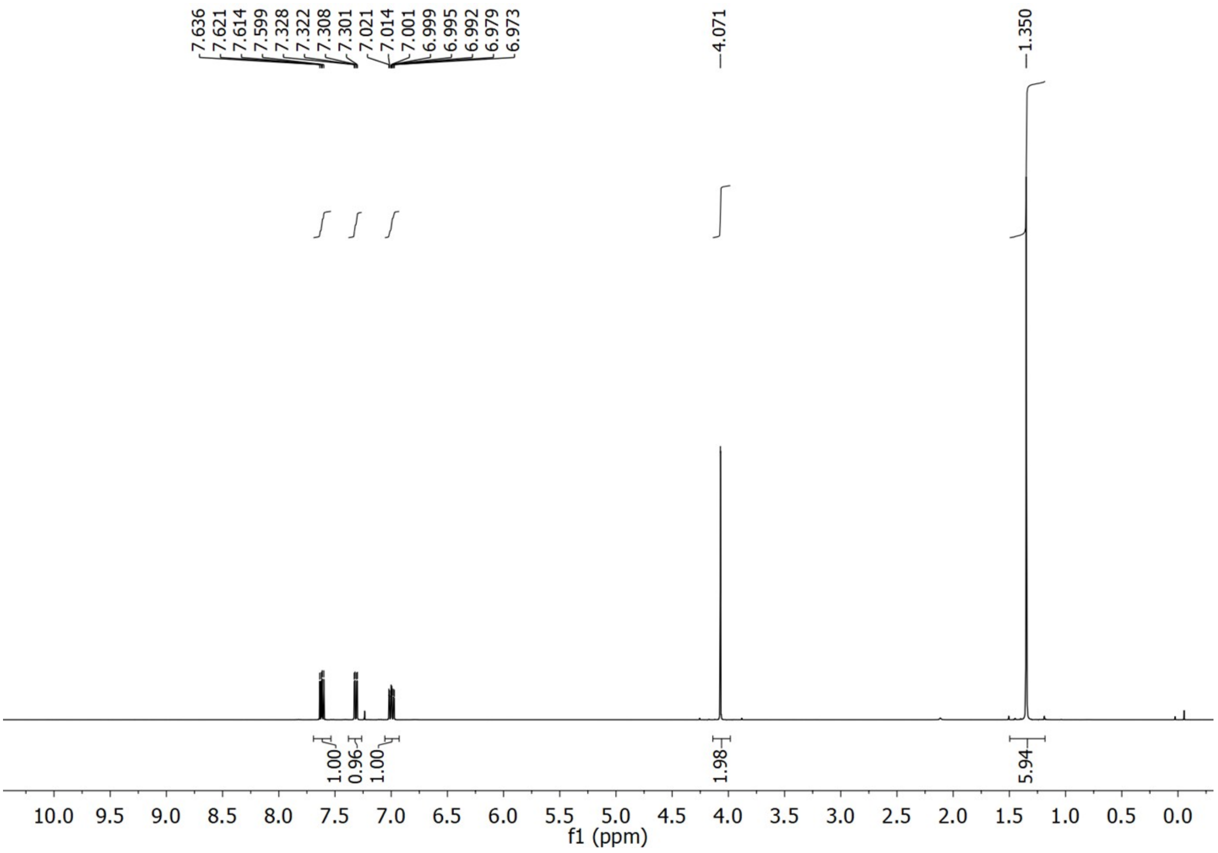

^{1}H NMR spectrum of **2b**.

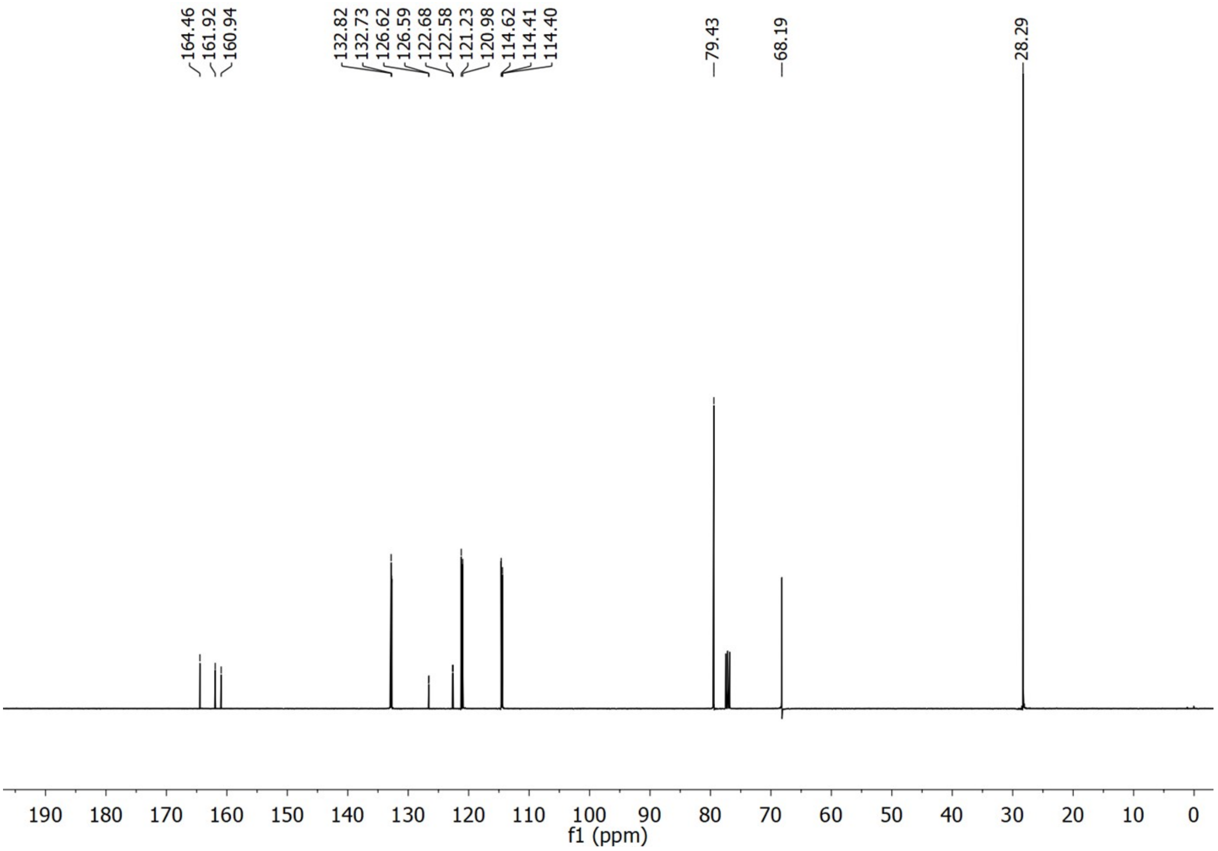

^{13}C NMR spectrum of **2b**.

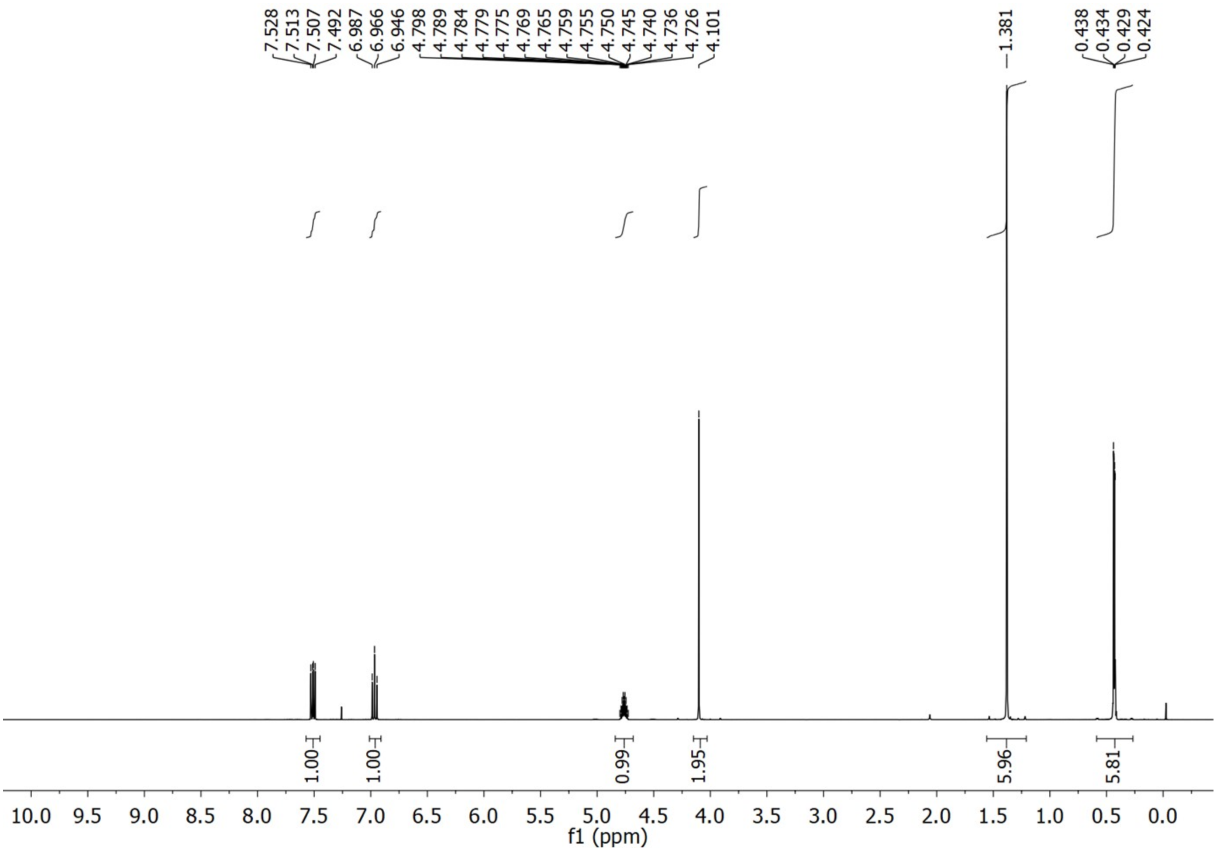

^{1}H NMR spectrum of **2b**.

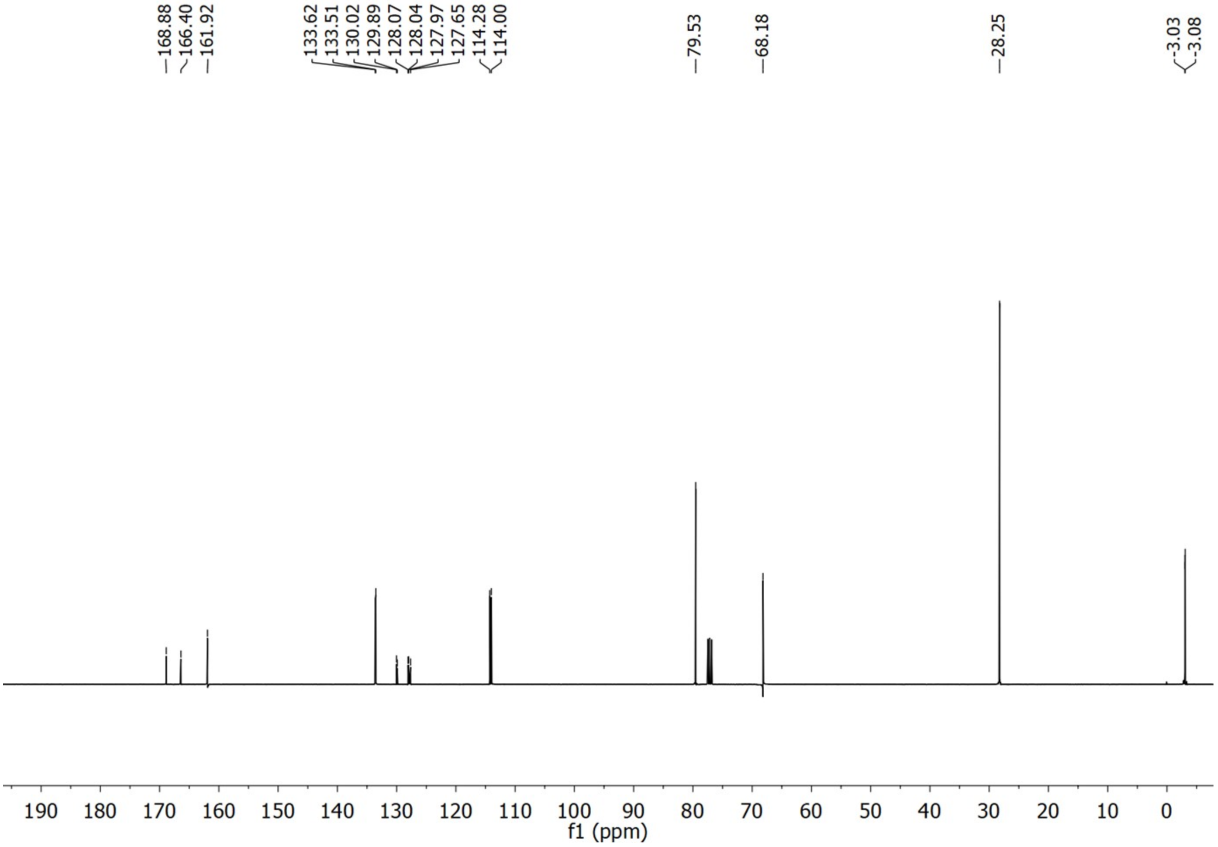

^{13}C NMR spectrum of **2b**.

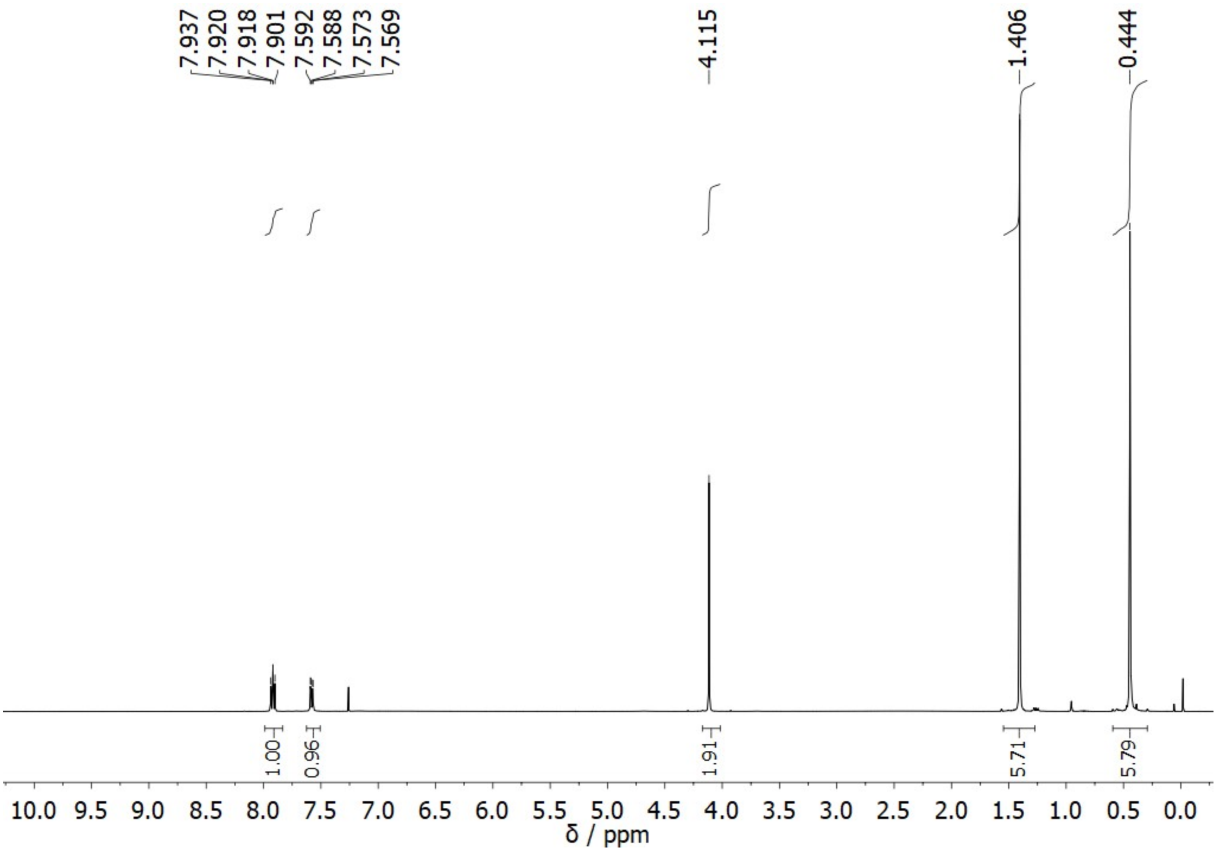

^{1}H NMR spectrum of **3a**.

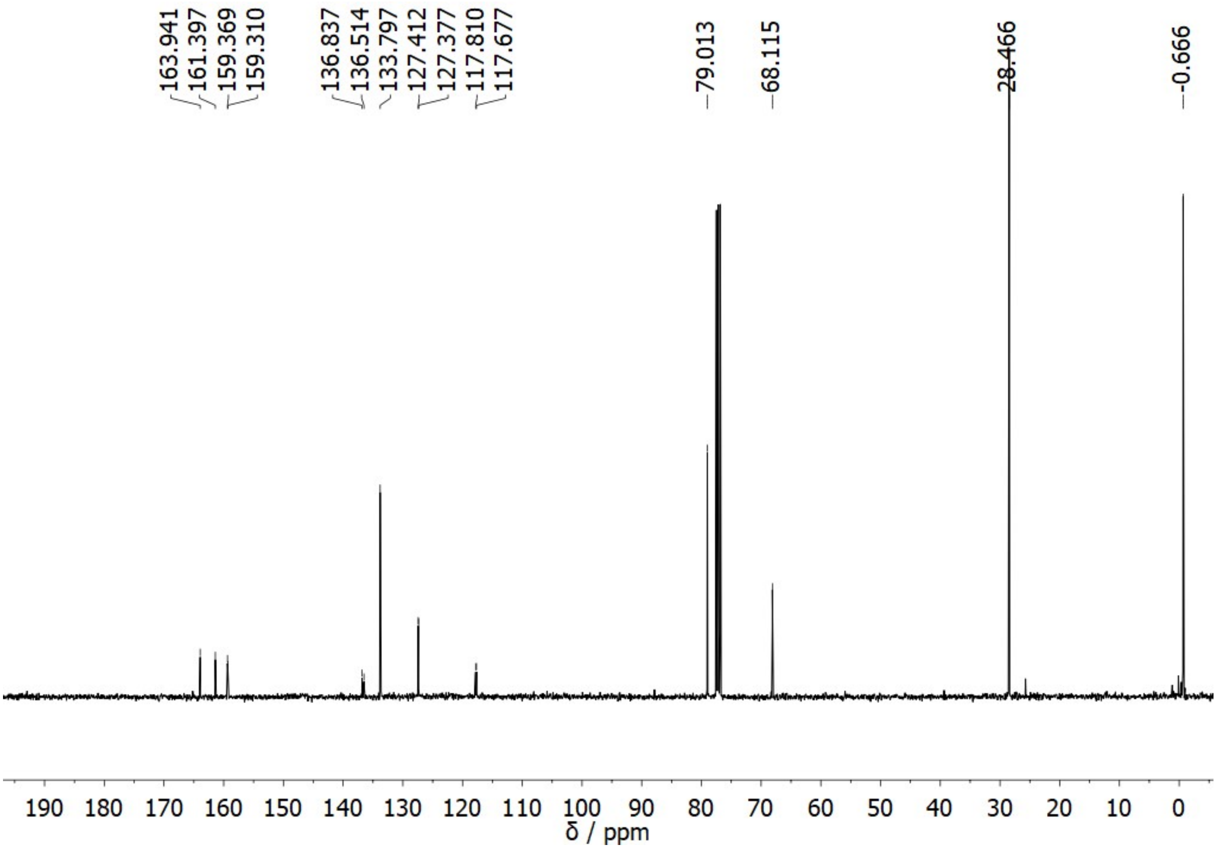

^{13}C NMR spectrum of **3a**.

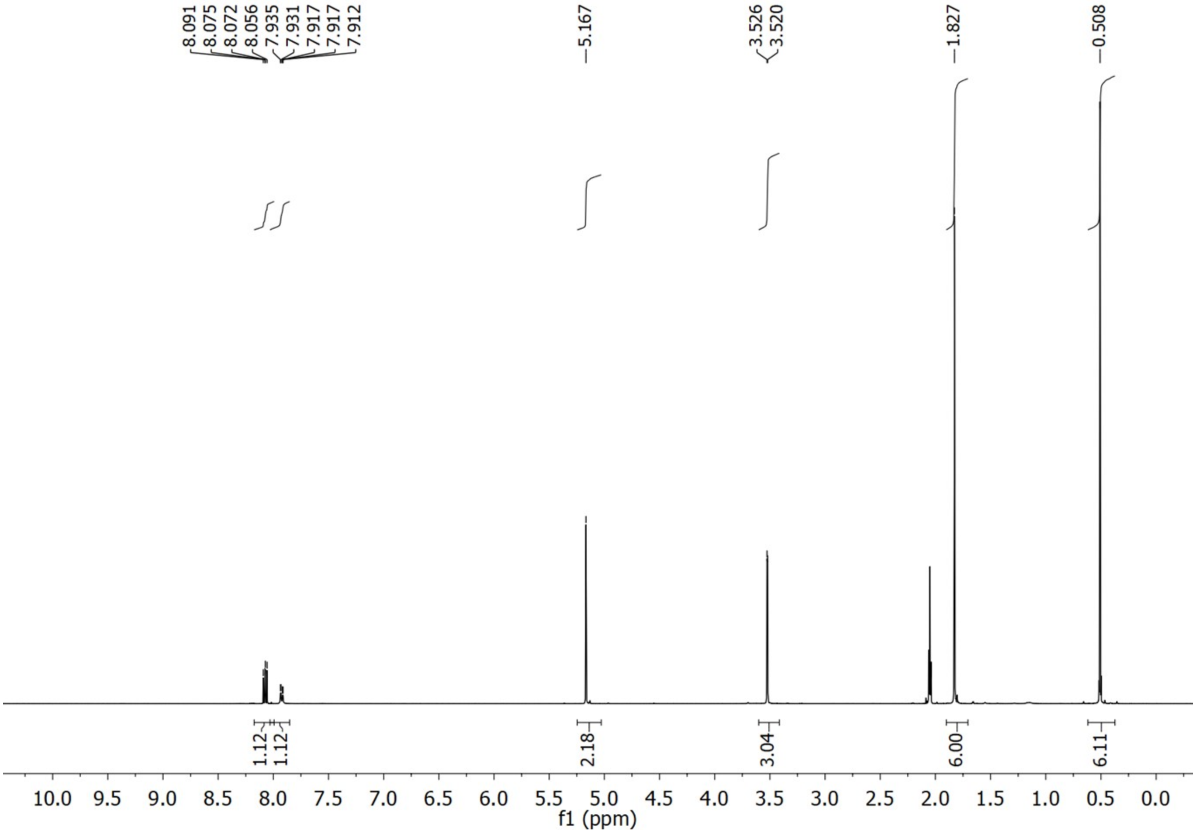

^{1}H NMR spectrum of **3b**.

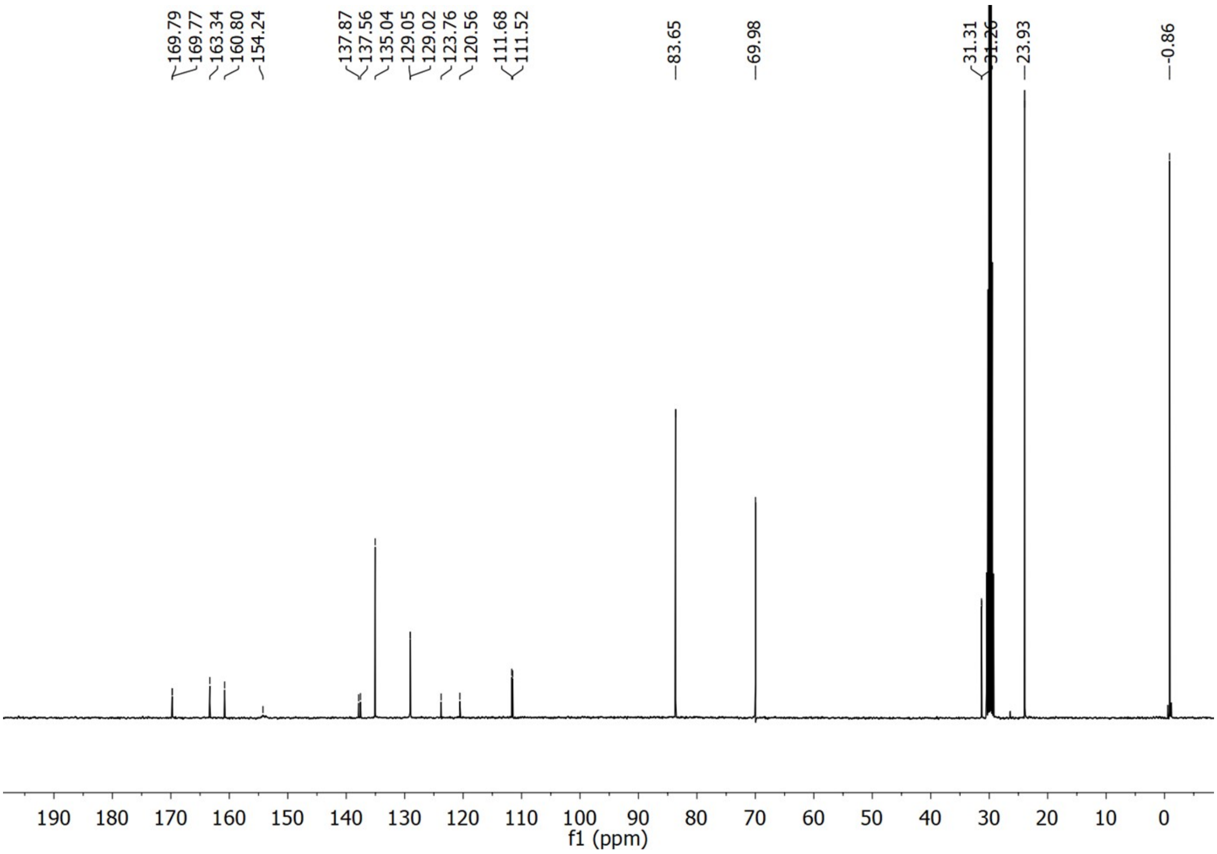

^{13}C NMR spectrum of **3b**.

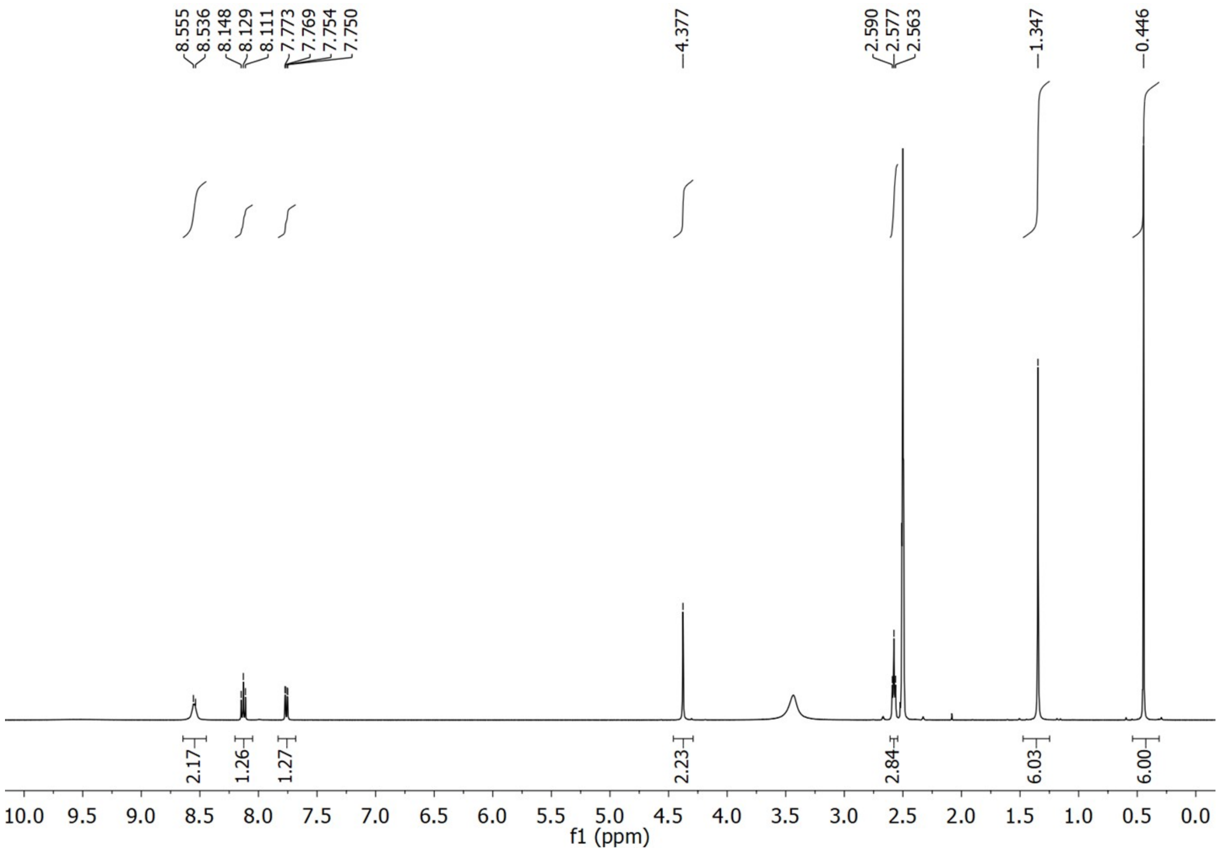

^{1}H NMR spectrum of **3c**.

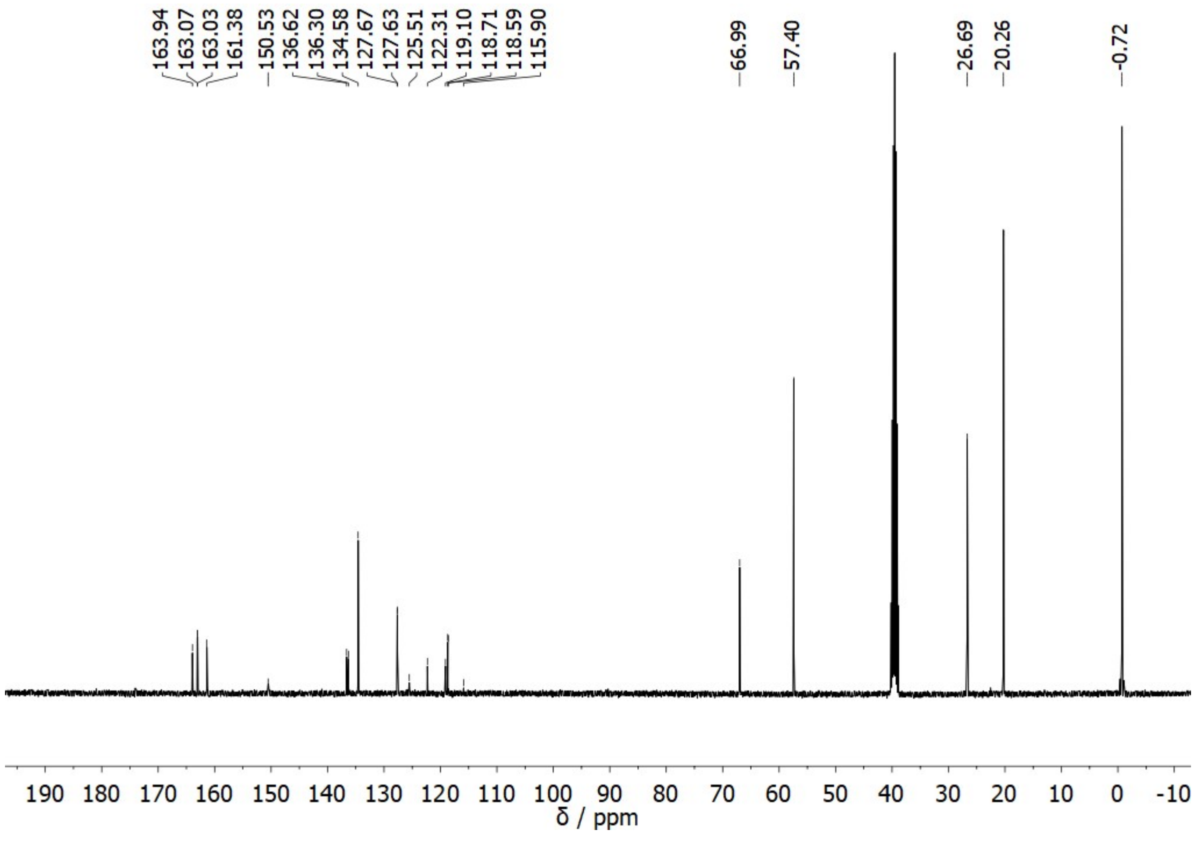

^{13}C NMR spectrum of **3c**.

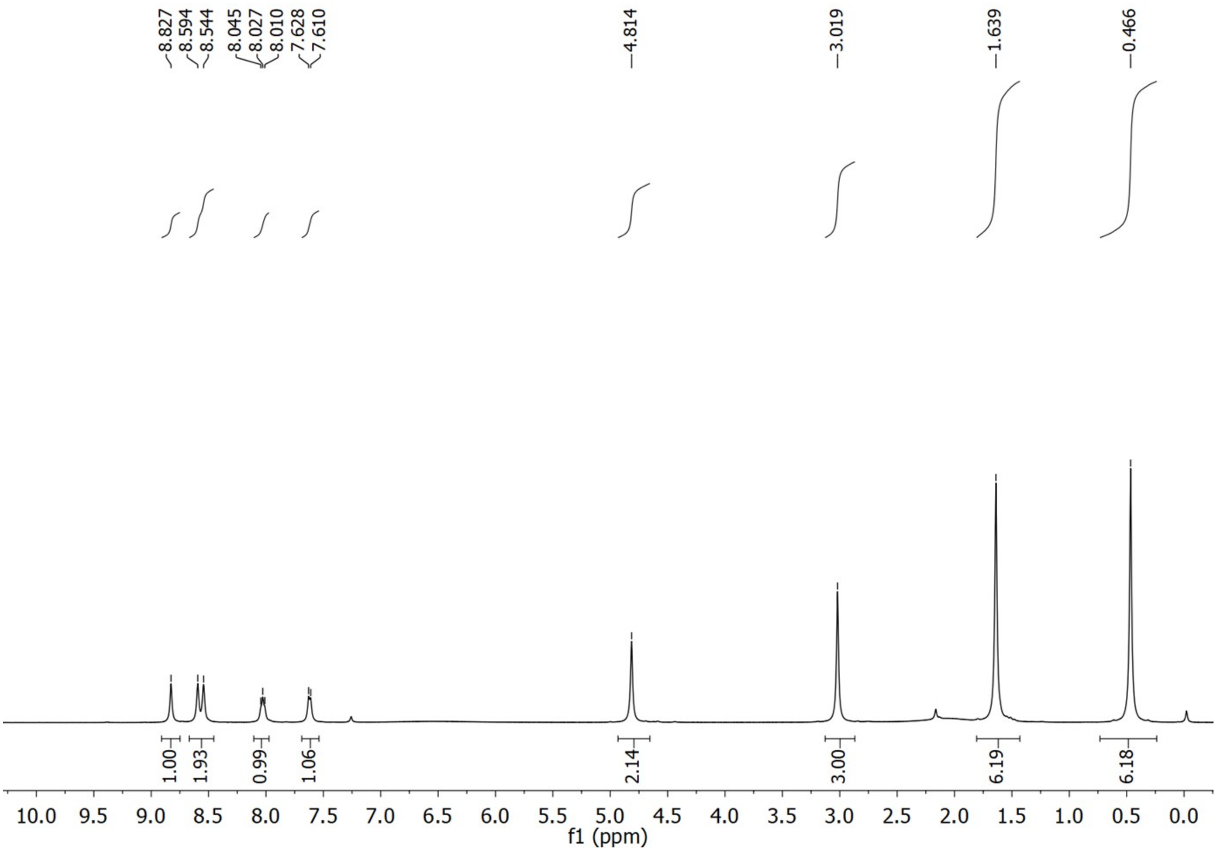

^{1}H NMR spectrum of **3d**.

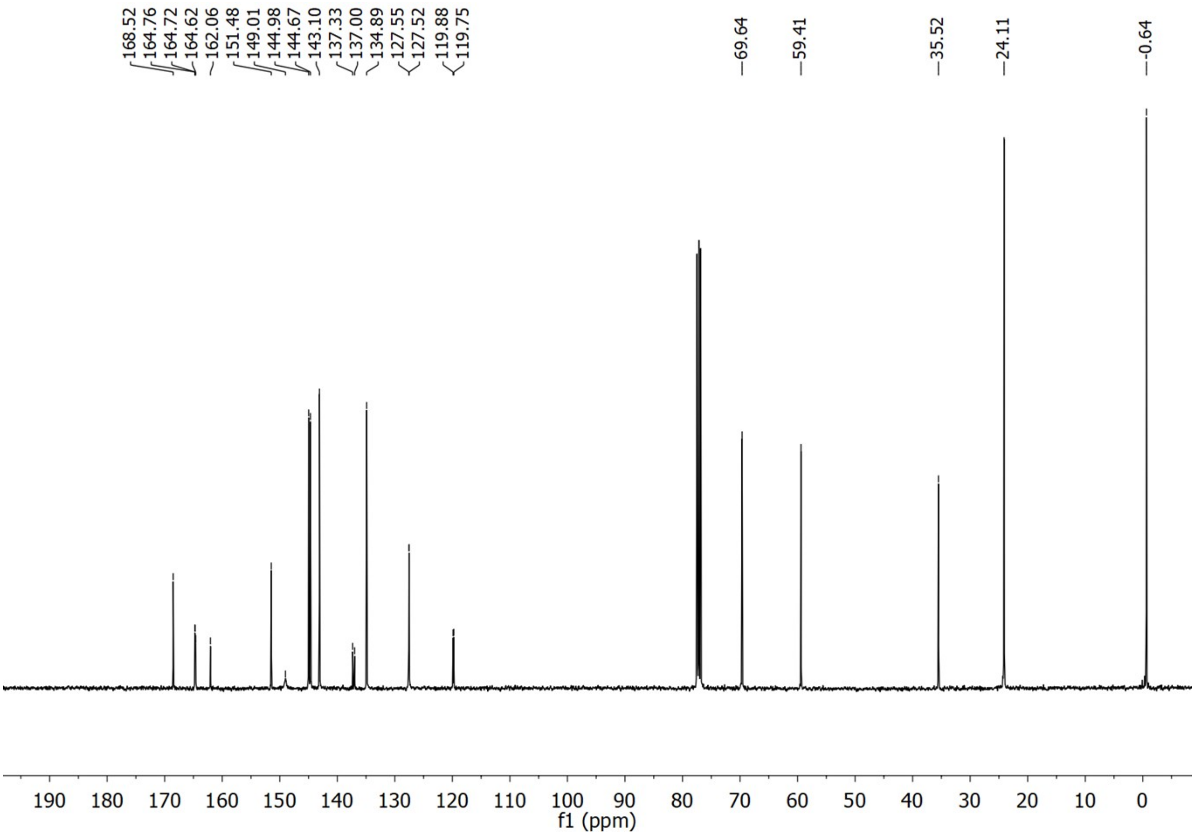

^{13}C NMR spectrum of **3d**.

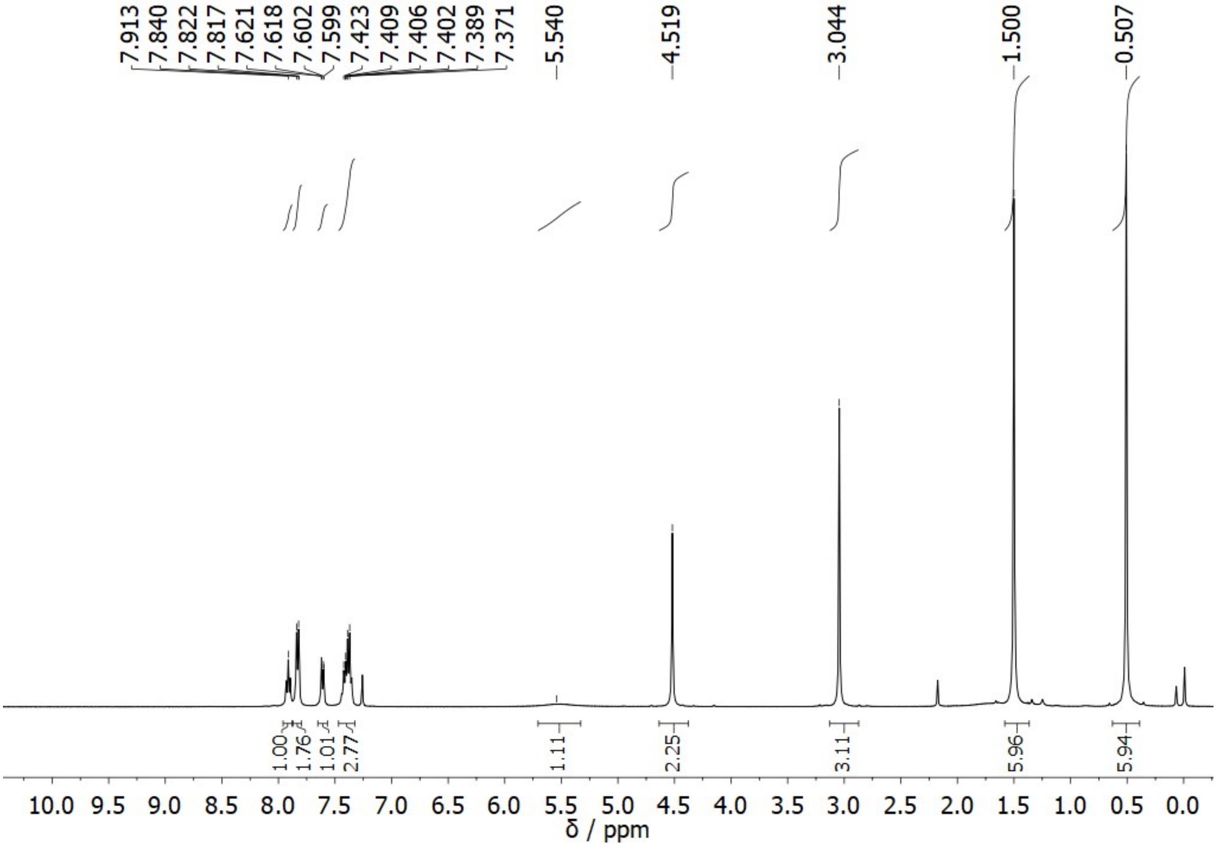

^{1}H NMR spectrum of **3e**.

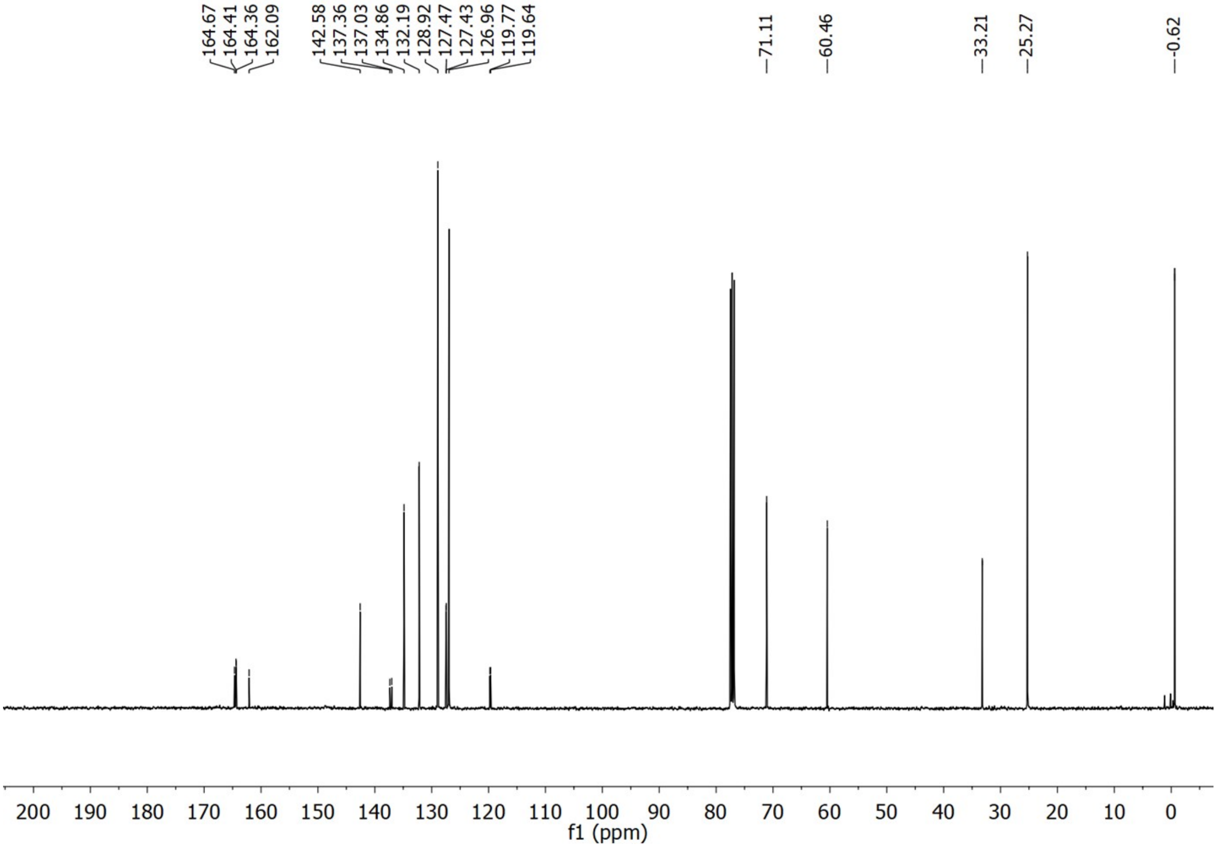

^{13}C NMR spectrum of **3e**.

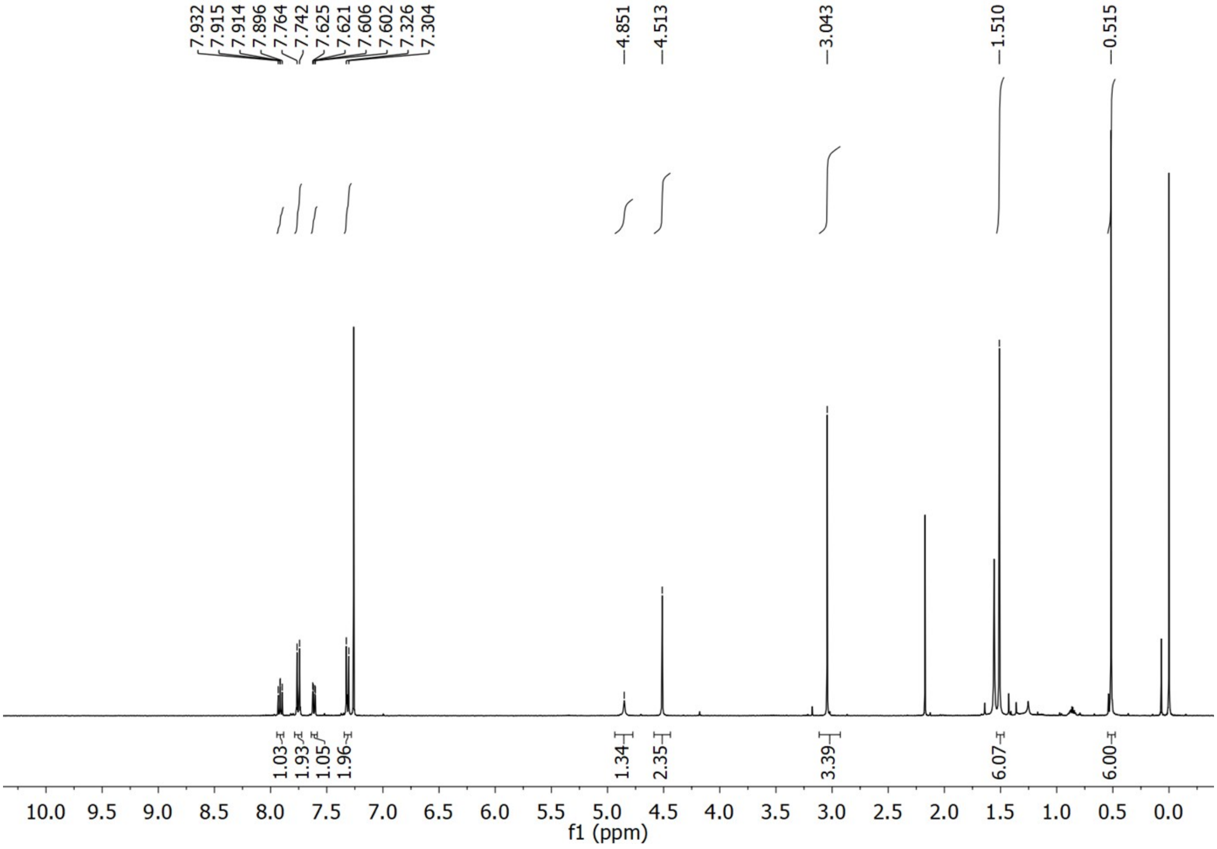

^{1}H NMR spectrum of **3f**.

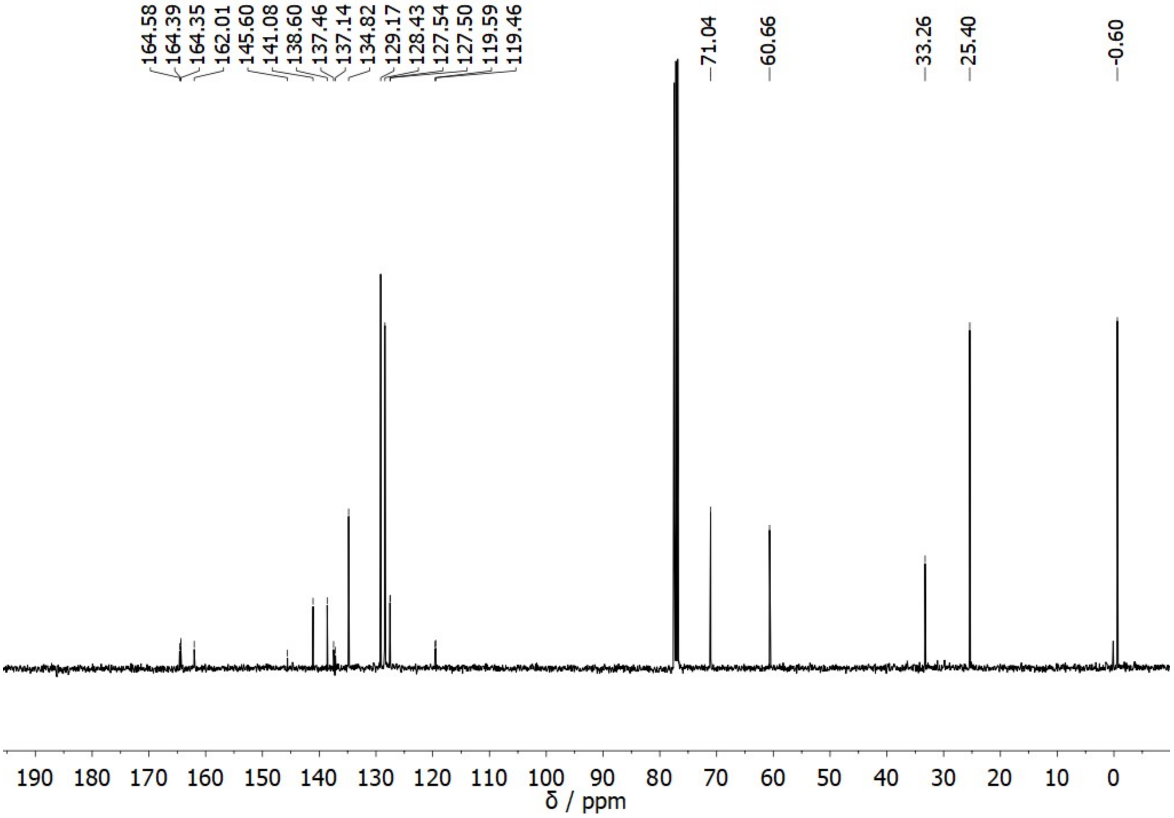

^{13}C NMR spectrum of **3f**.

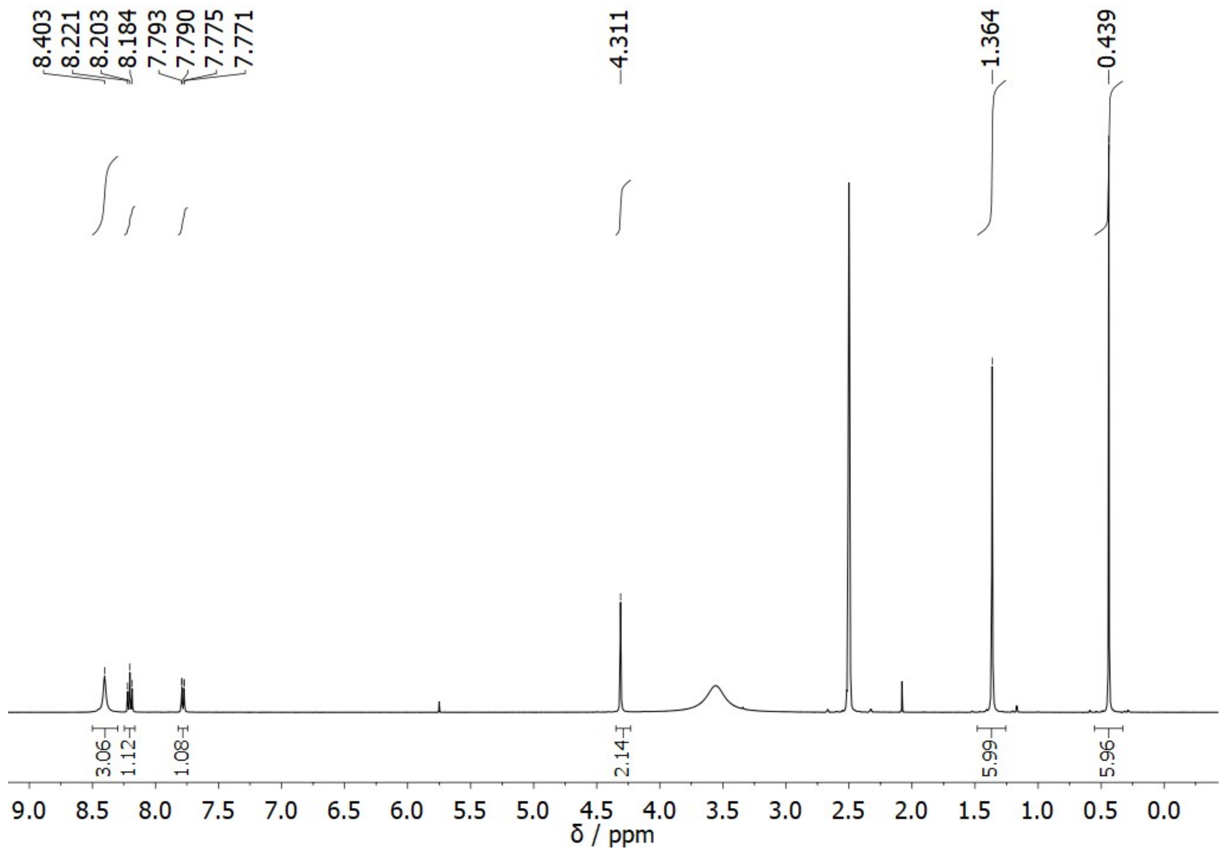

^{1}H NMR spectrum of **3g**.

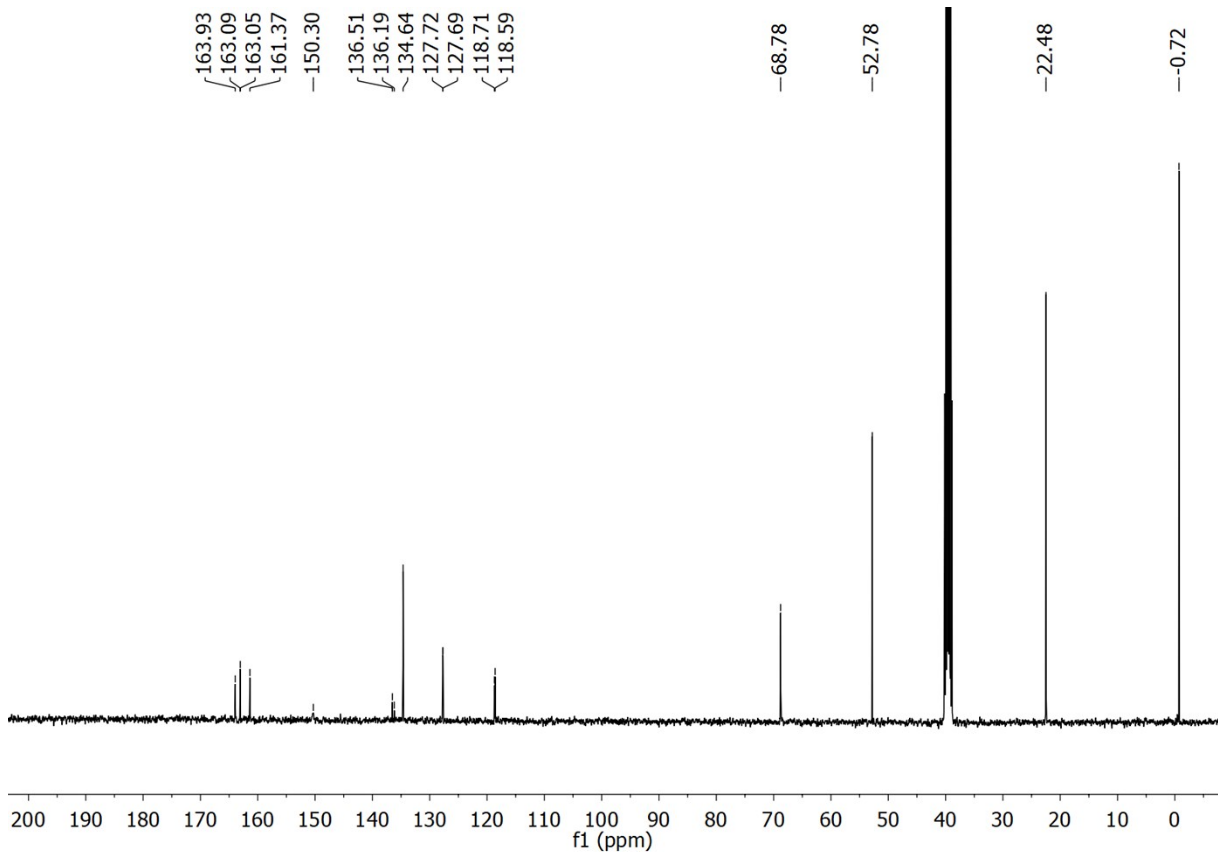

^{13}C NMR spectrum of **3g**.

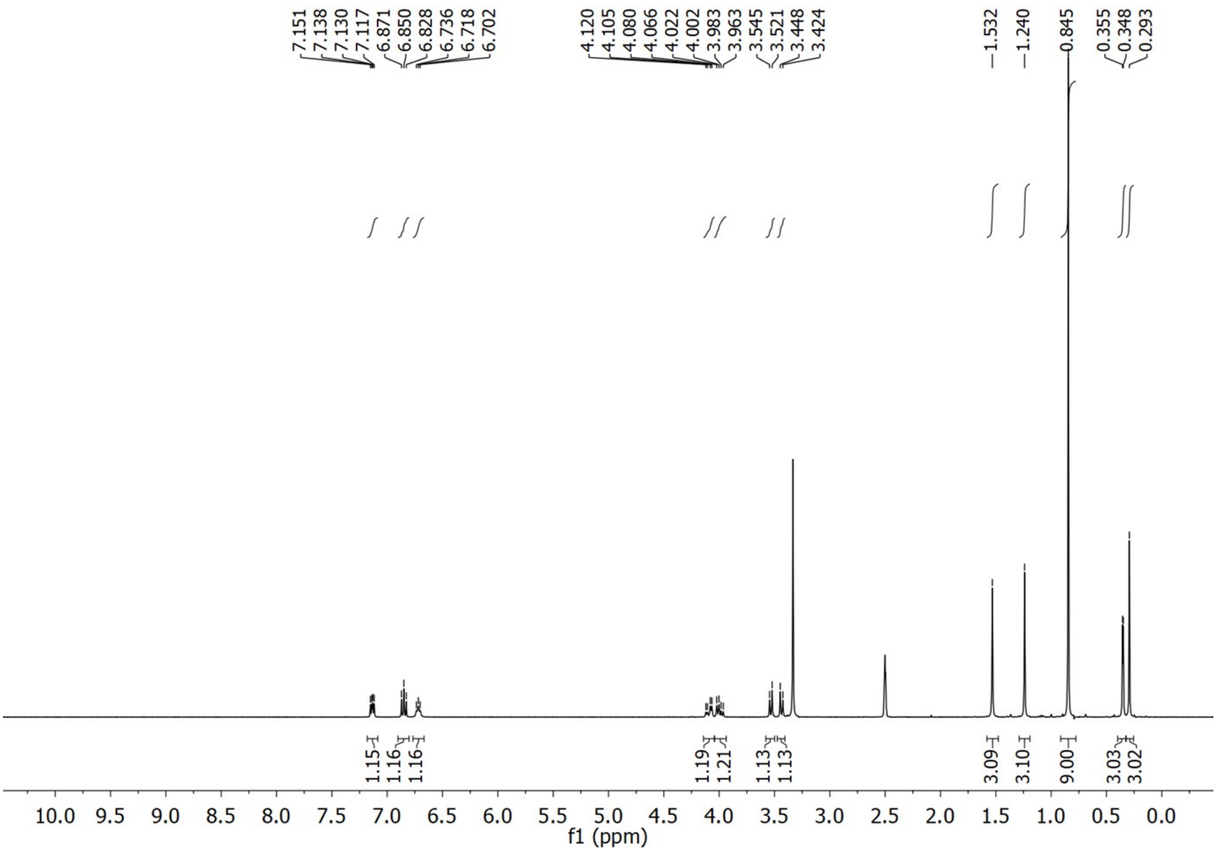

^{1}H NMR spectrum of **4a**.

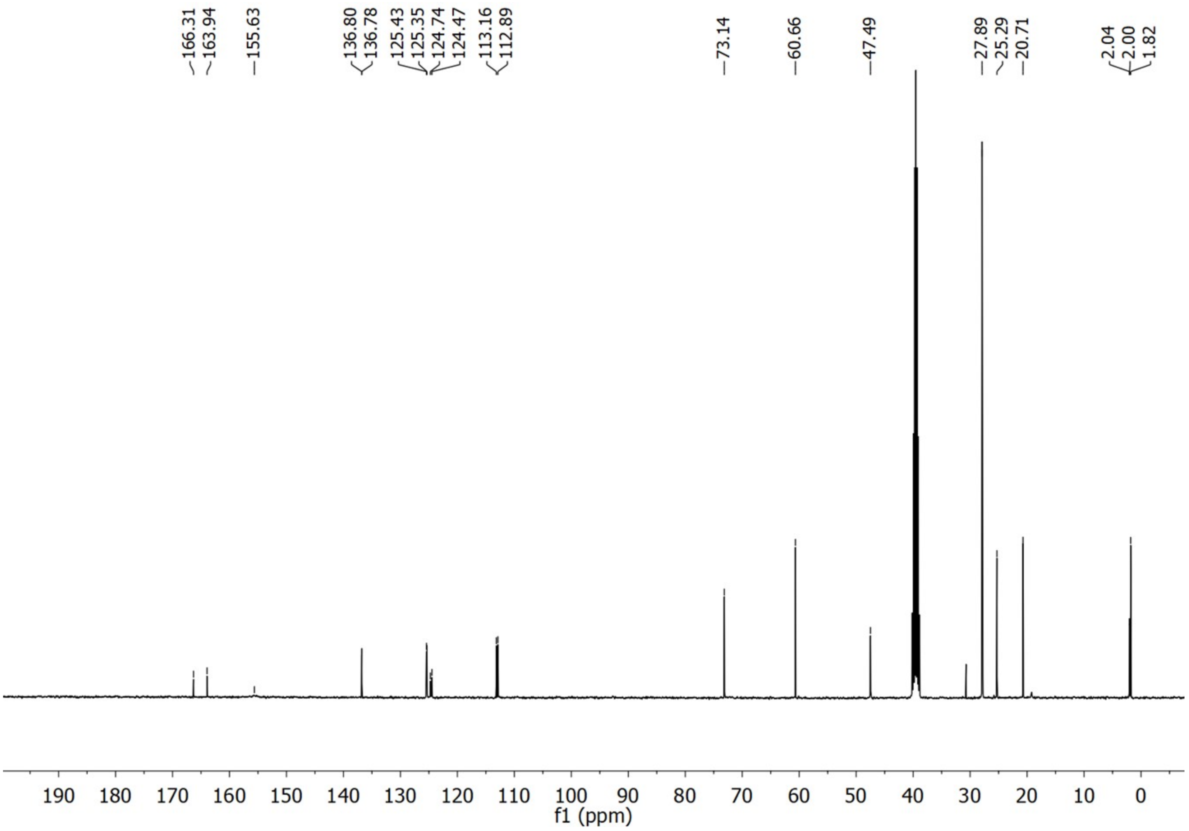

^{13}C NMR spectrum of **4a**.

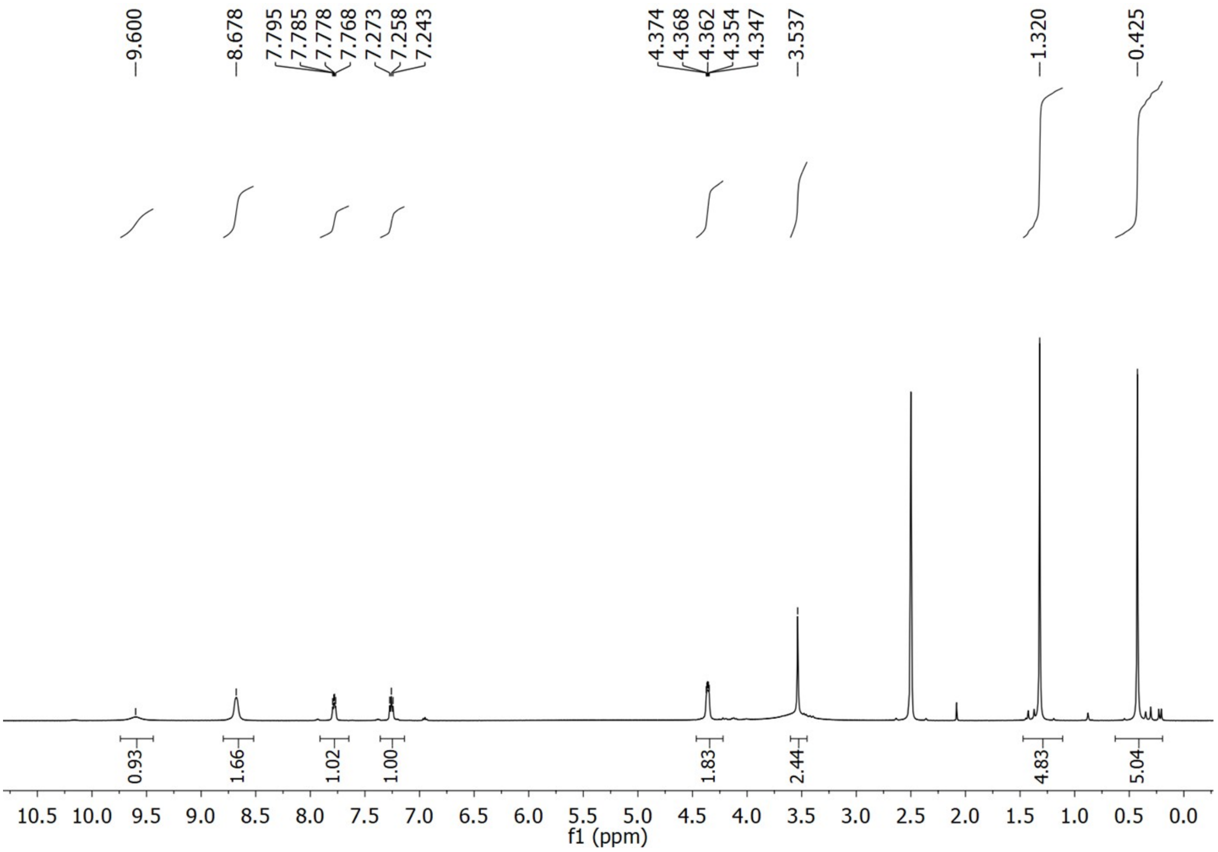

^{1}H NMR spectrum of **4b**.

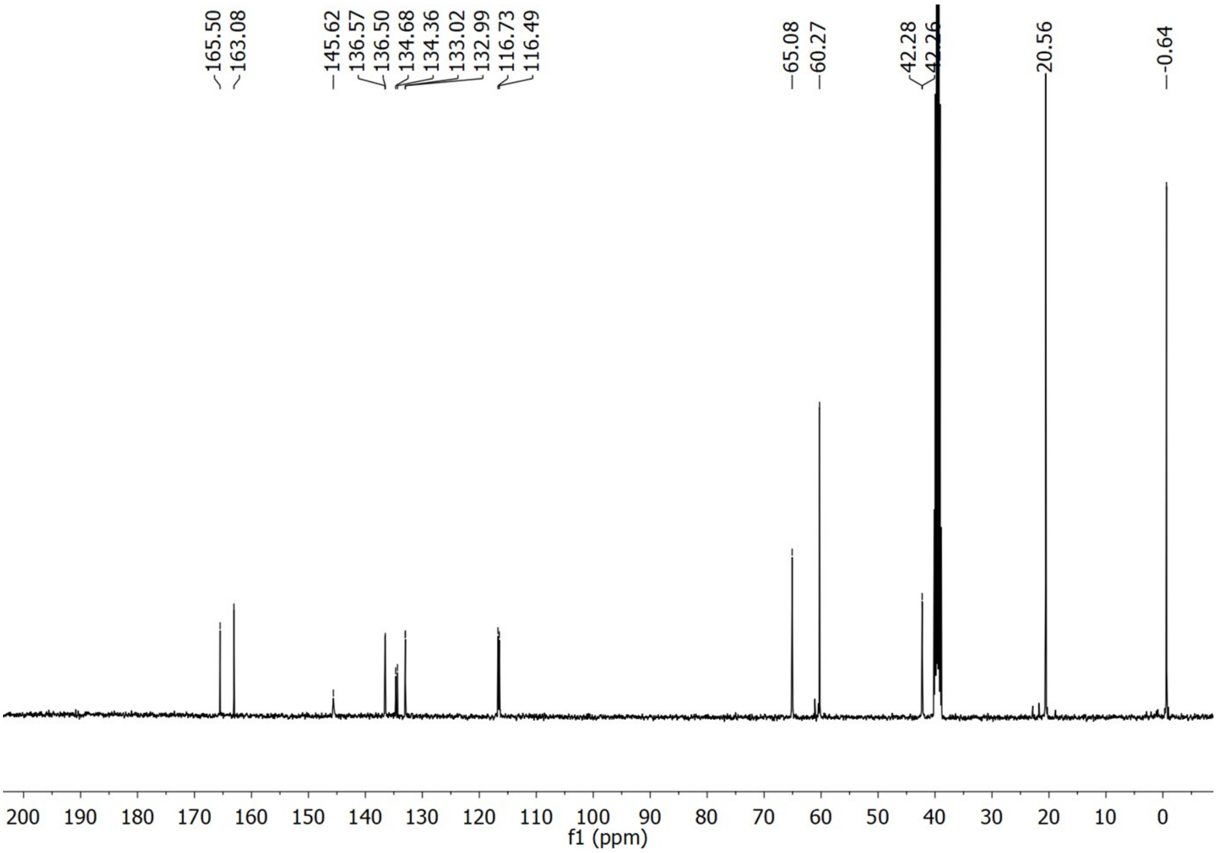

^{13}C NMR spectrum of **4b**.

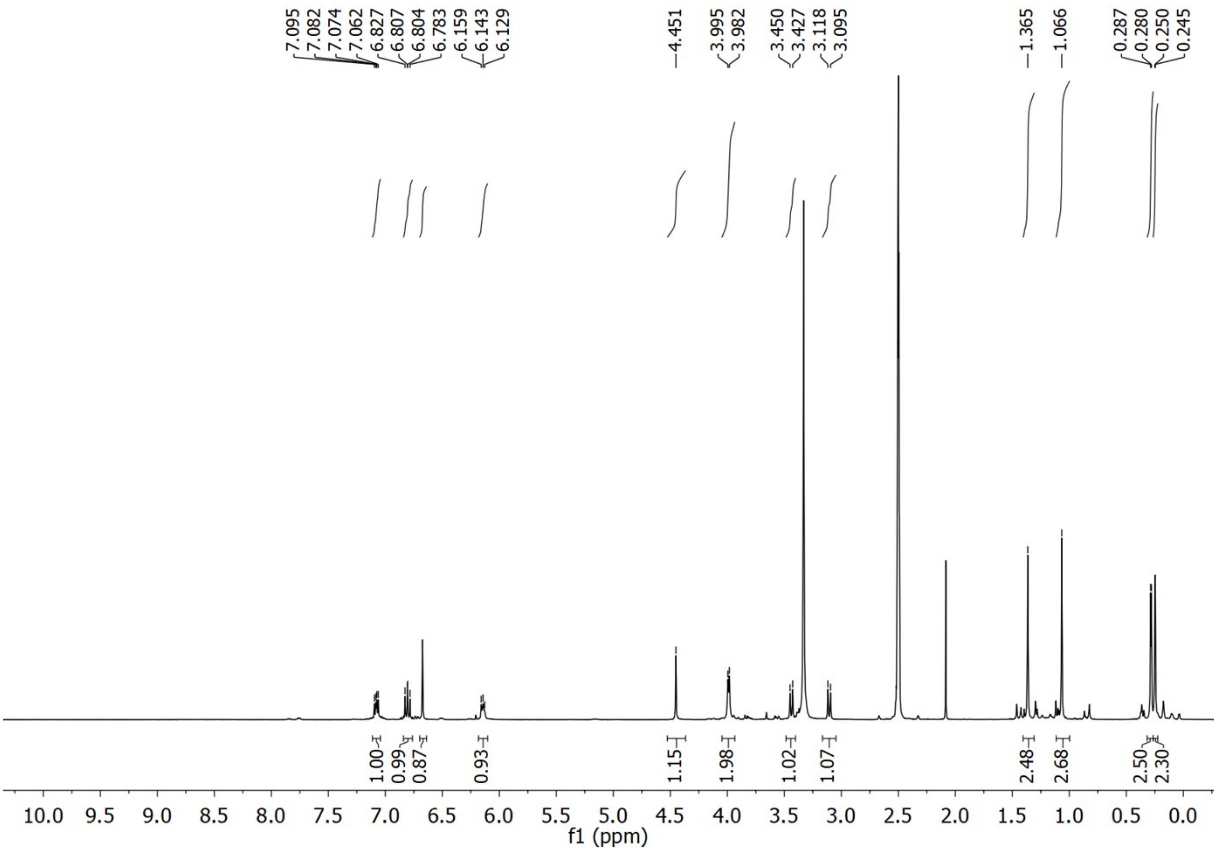

^{1}H NMR spectrum of **4c**.

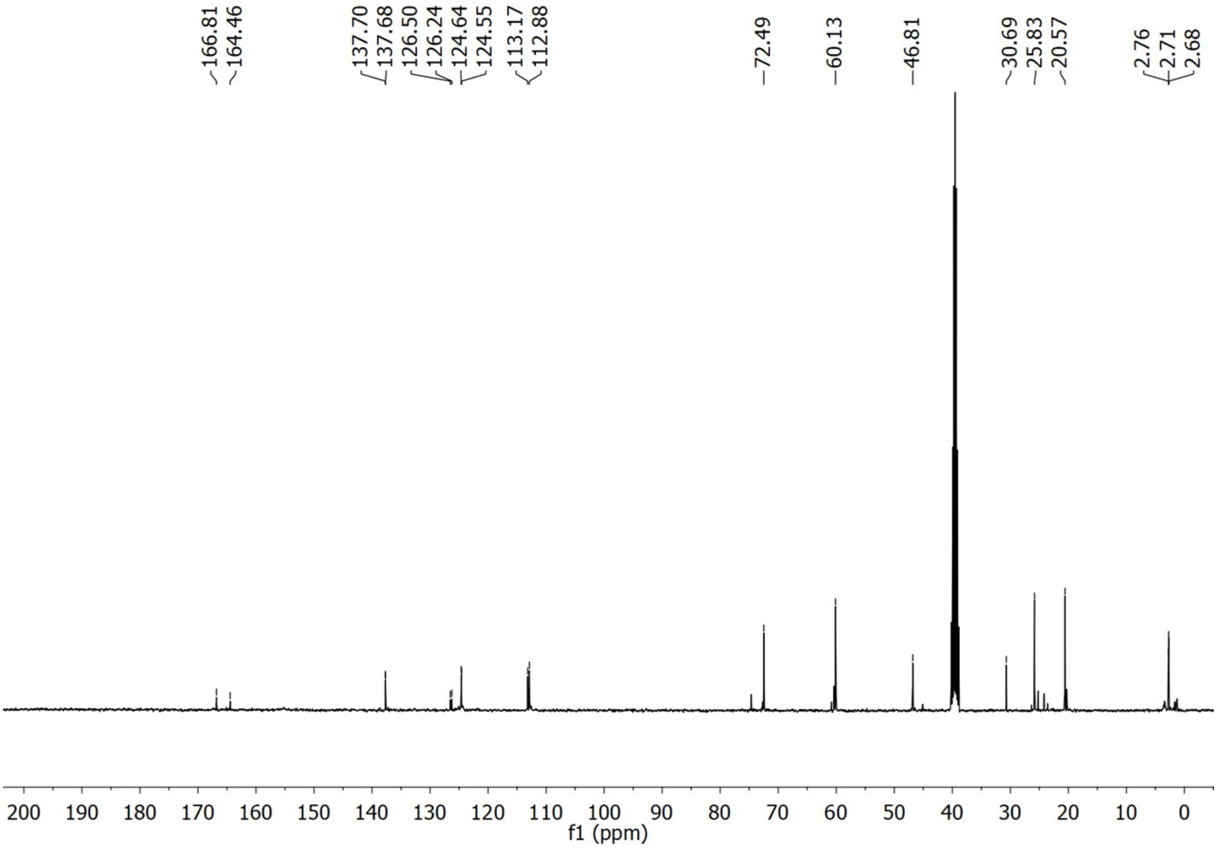

^{13}C NMR spectrum of **4c**.

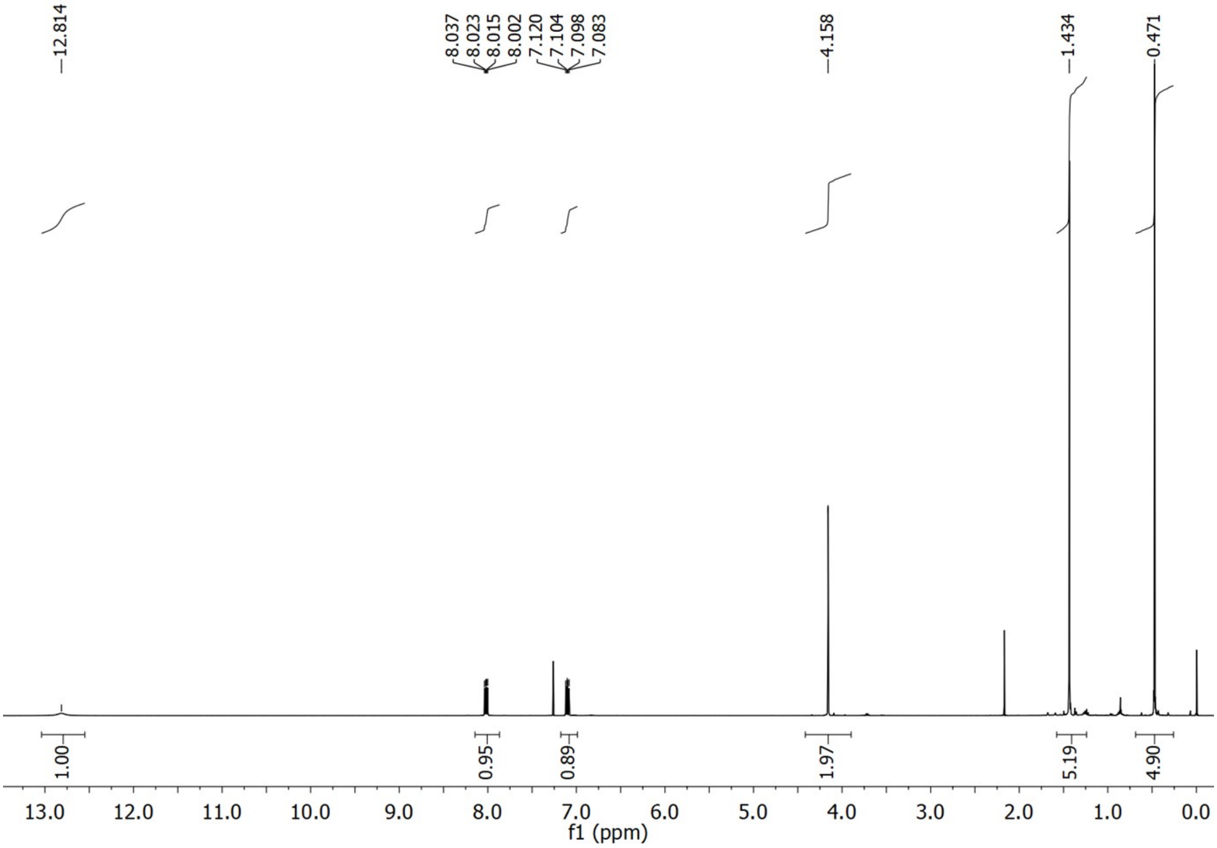

^{1}H NMR spectrum of **5a**.

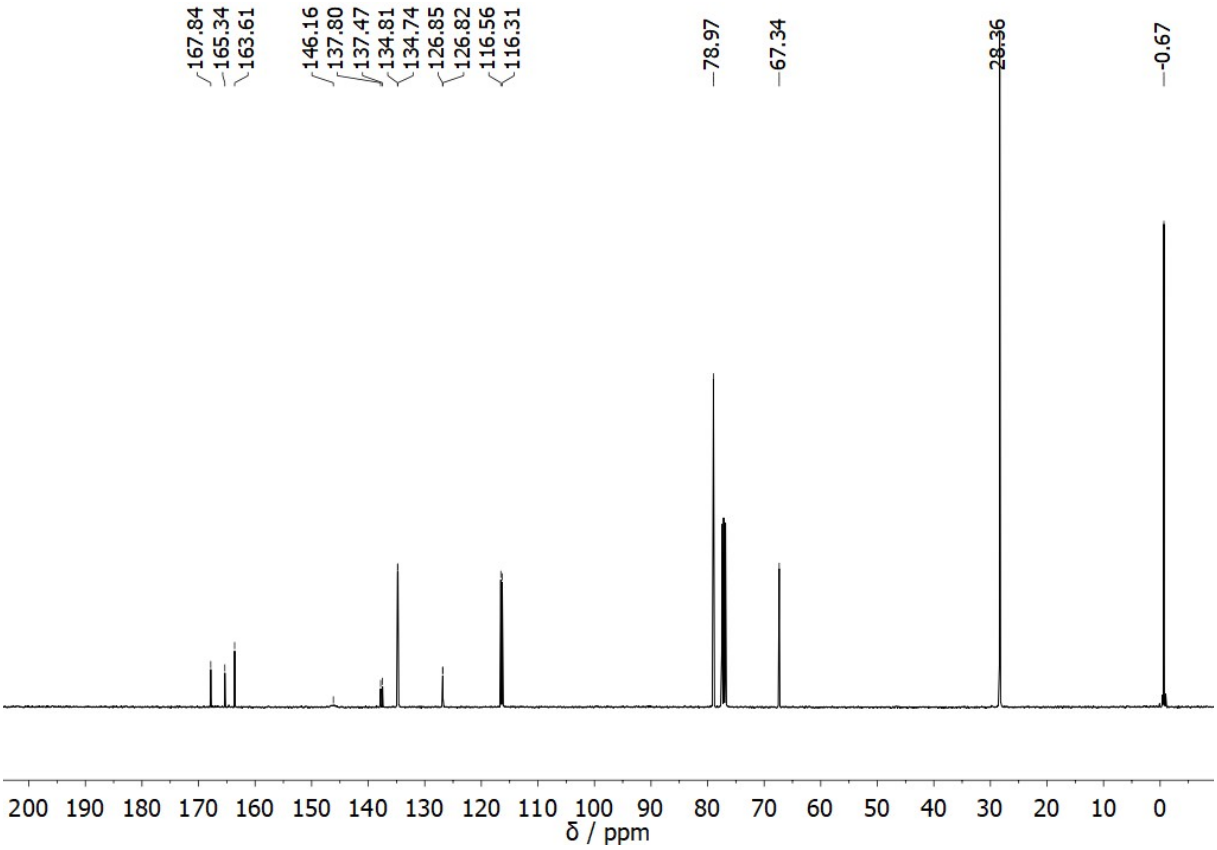

^{13}C NMR spectrum of **5a**.

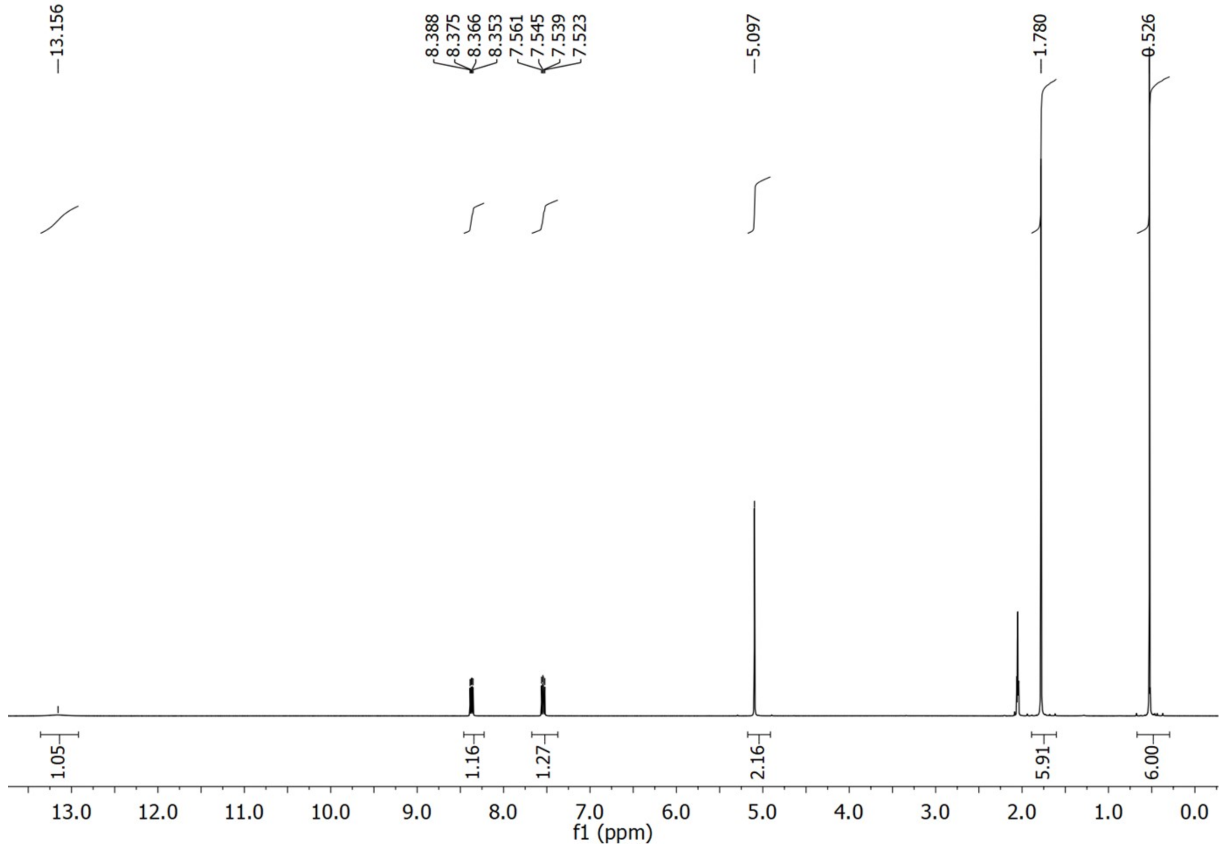

^{1}H NMR spectrum of **5b**.

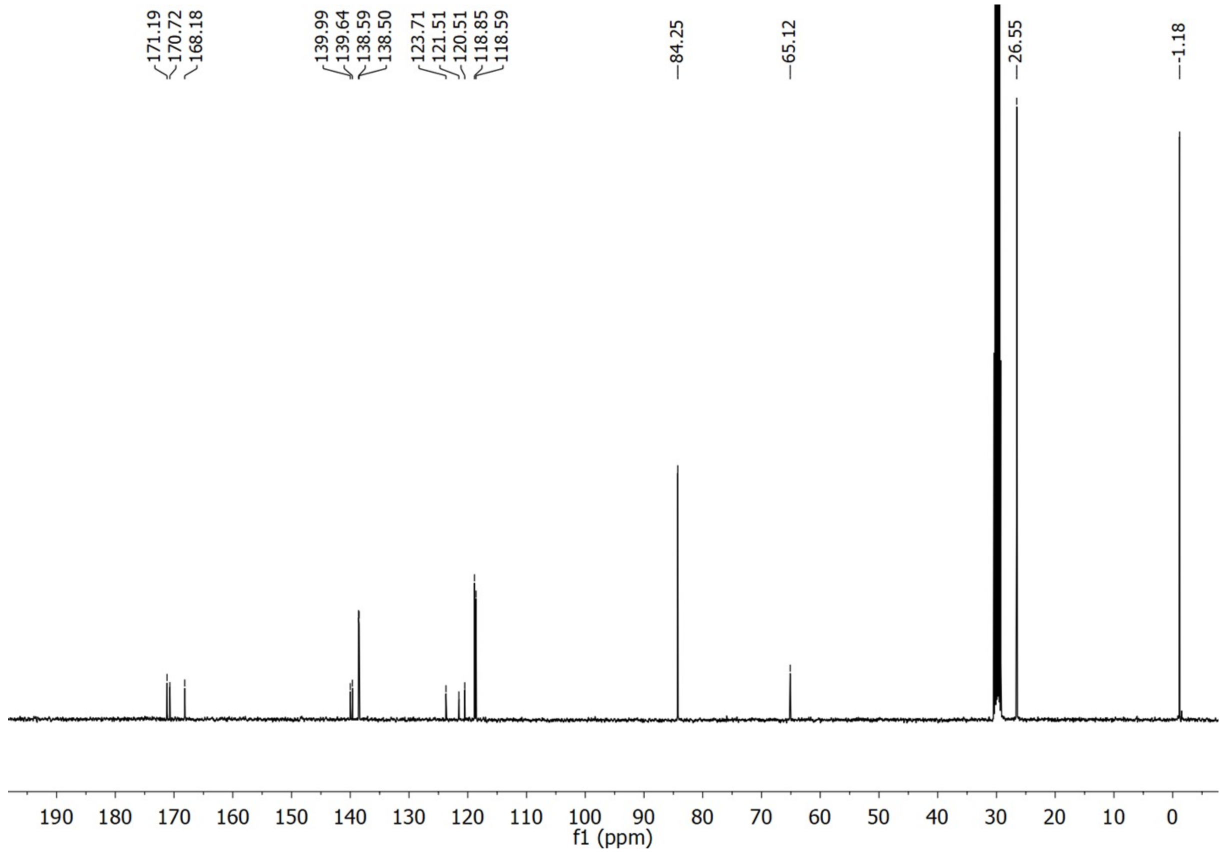

^{13}C NMR spectrum of **5b**.

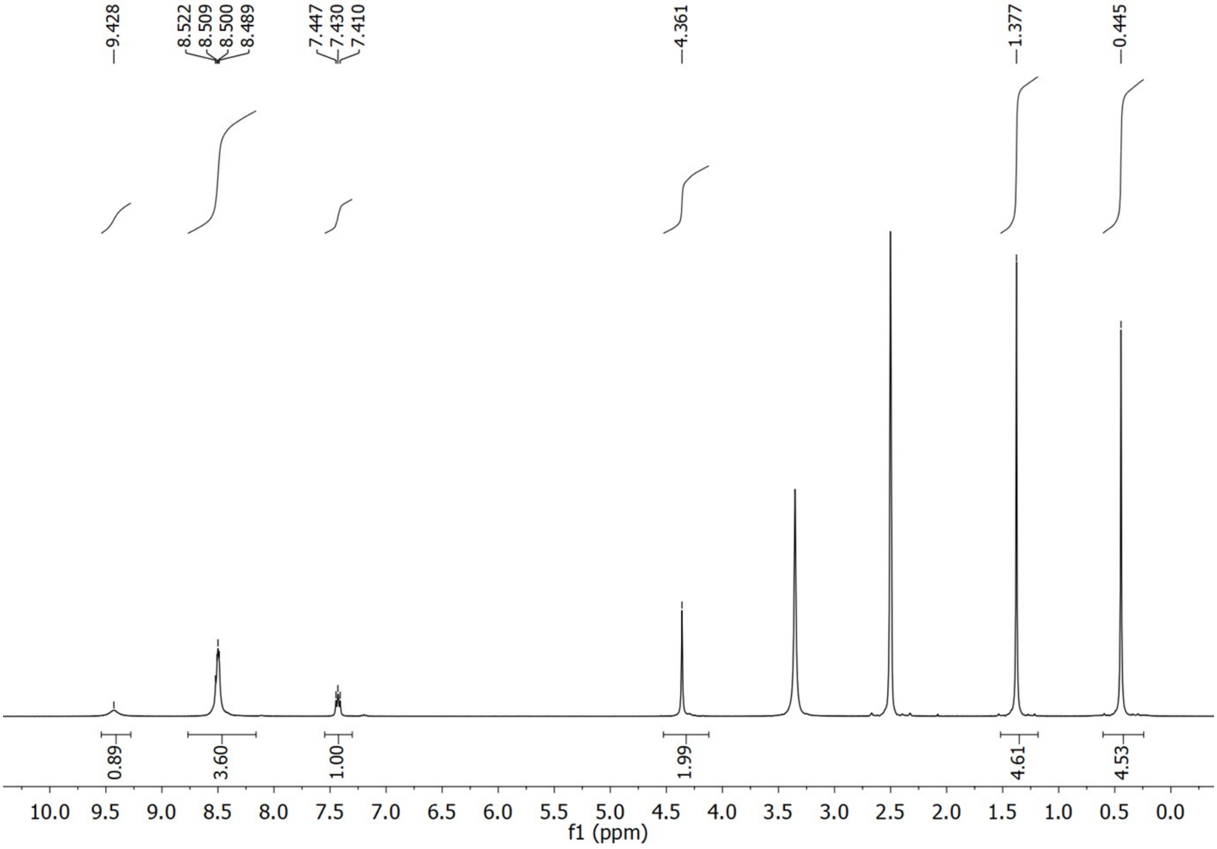

^{1}H NMR spectrum of **5c**.

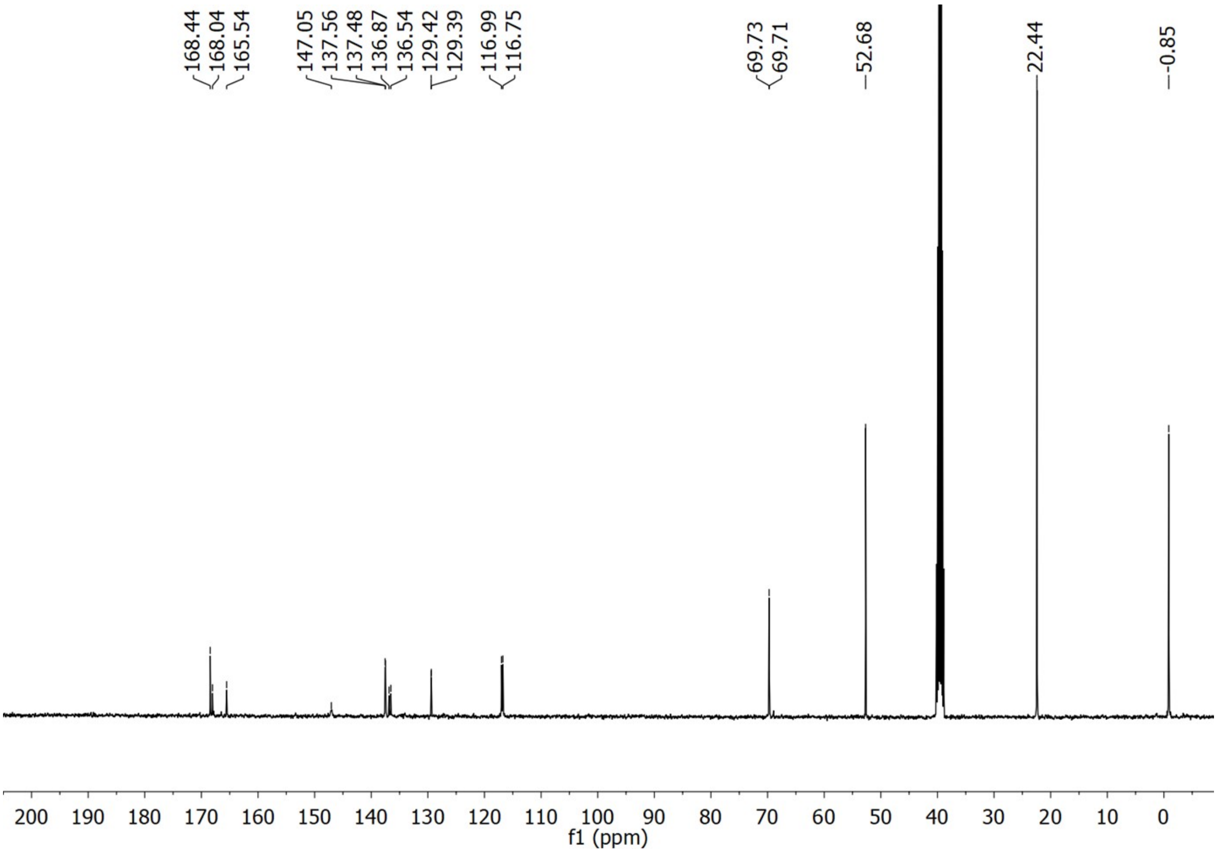

^{13}C NMR spectrum of **5c**.

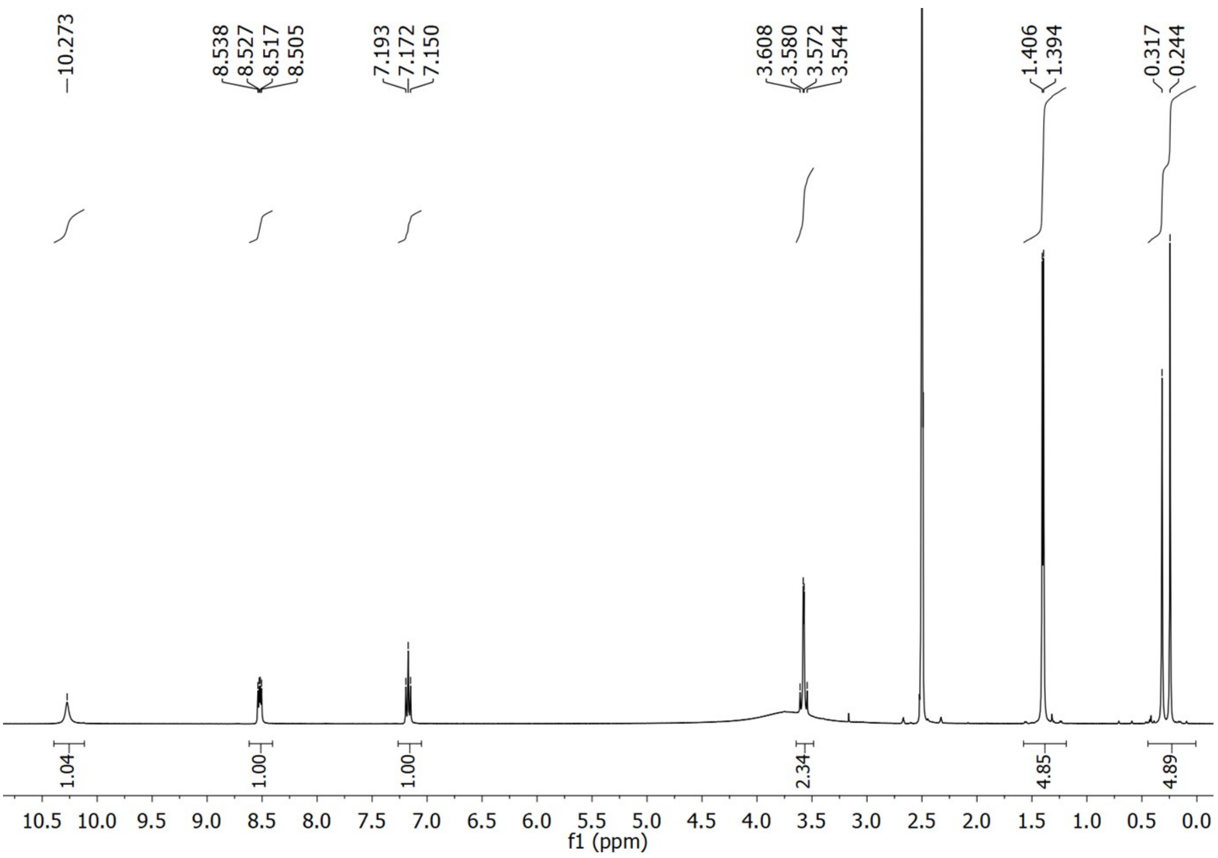

^{1}H NMR spectrum of **5d**.

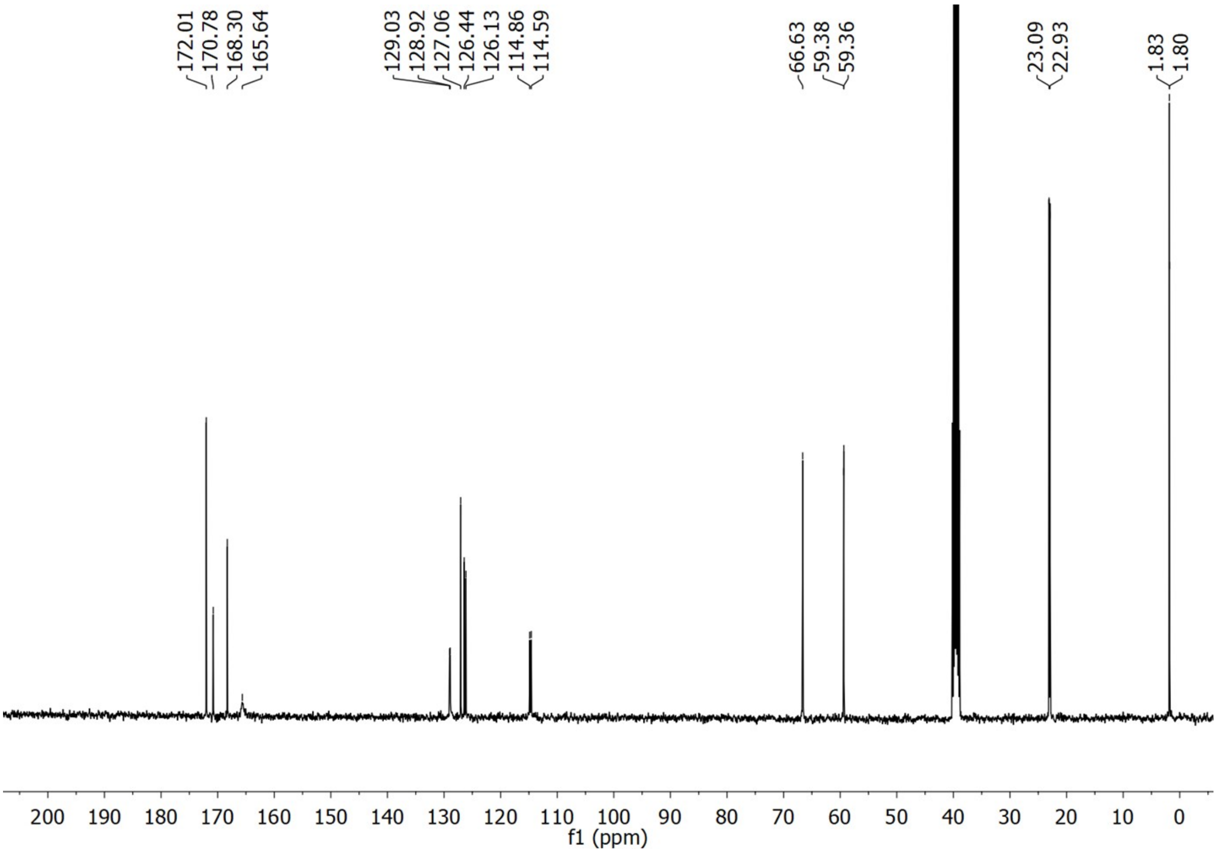

^{13}C NMR spectrum of **5d**.

References

1. Clinical and Laboratory Standards Institute (CLSI), Methods for dilution antimicrobial susceptibility tests for bacteria that grow aerobically, Approved Standard, Document M07-A9, 9th ed., CLSI, 940 West Valley Road, Wayne, PA, USA, 2012.
2. ETEST Application guide, BioMerieux. http://www.biomerieux-usa.com/clinical/etest.
3. ETEST for antifungal susceptibility testing – research gate, AB BIODISK. https://www.researchgate.net/...fungal.../Etest.pdf
4. M. A. Pfaller, M. Bale, B. Buschelman, M. Lancaster, A. Espinel-Ingroff, J. H. Rex and M. G. Rinaldi., *J. Clin. Microbiol.*, 1994, **32**, 1650–1653.
